# Supplementary material for: Students’ and staffs’ views and experiences of asymptomatic testing on a university campus during the COVID-19 pandemic in Scotland: a mixed methods study
Source: BMJ Open. 2023 Mar 20;13(3):e065021. doi: 10.1136/bmjopen-2022-065021 (PMC10030276; doi:10.1136/bmjopen-2022-065021)
Supplement: Supplementary data [file bmjopen-2022-065021supp001.pdf]

Supplementary File 1: TestEd Participant Pilot Survey

Notes for entry online:

BLUE = The question/variable name.
RED = Skip, display, or loop logic.
GREEN = New Block
PURPLE = Forced response

- -

INTRO BLOCK

INTRODUCTION PAGE

I This survey is about student and staff experiences of asymptomatic COVID-19 testing at the University, delivered via the TestEd programme. Before agreeing to take part and proceeding to answer the survey questions, we'd like to remind you of what the survey involves and how the responses you provide will be used.

This information can also be found in the Participant Information Sheet for TestEd, which is available at:

https://www.ed.ac.uk/files/atoms/files/participant_information_sheet_v3.0_28_january_2021_clean.pdf

The survey will ask about your experiences of participating in TestEd. Your responses will help improve the programme as it is rolled out. Participation is voluntary and the survey should take about 10-15 minutes to complete.

Your anonymous survey data will be imported into quantitative data analysis software for analysis by the research team. The survey data will be retained on our server for a minimum of 5 years after the end of the study.

The anonymised results of this survey may be quoted in reports and academic publications produced by the study team. Your name will never be used in any of these reports or publications and they will not include any personal identifiable information about you.

At the end of this survey we ask if you would be willing to be re-contacted to participate in a follow-up interview with a researcher if you receive a positive result for Covid-19 from the TestEd programme. This interview is voluntary.

TestEd Participant Pilot Survey. 15th of April 2021

CONSENT

In agreeing to participate in this survey, you confirm the following:

1. I confirm that I have read and understood the Participant Information Sheet for TestEd.

2. I understand that my participation is voluntary and that I can ask to withdraw at any time without giving a reason and without my legal rights being affected.

3. I understand that once the survey form is submitted it will not be possible to withdraw from the survey. This is because no identifiable information will be stored with the survey data.

4. I confirm that I am happy for my survey responses to be linked to anonymised demographic data (age, gender, ethnicity, staff or student, whether living in University owned accommodation or elsewhere) provided by me when I registered to participate in the TestEd programme

5. I confirm that I am happy for anonymised data from this survey to be published for research purposes.

6. I understand that my anonymised data will be stored for a minimum of 5 years and may be used in future ethically approved research.

Should you have any further questions about this survey or any element of TestEd please contact us via TestEd@ed.ac.uk

By ticking this box, I agree to the above consent points and to take part in the above study

TestEd Participant Pilot Survey. 15th of April 2021

Q8

How much time did you take out of your day to provide a TestEd sample (not including travel time, i.e. collecting a sample pack, providing the sample and dropping off your sample)?

a. 1-2 minutes
b. 2-5 minutes
c. 5-10 minutes
d. More than 10 minutes

- -

TEST TRUST BLOCK

Q15

Do you believe that the result you received from Test Ed was accurate?

a. Yes
b. No
c. Unsure

Q16

Why did you believe the result was accurate/inaccurate?

[free text box]

Q17

Does the availability of the TestEd programme make you feel reassured about working/studying on campus?

a. Yes
b. No
c. Unsure [skip to Q19]

Q18

Could you explain a bit more about why you felt reassured or not?

[free text box]

- -

POST-TEST ATTITUDES AND BEHAVIOUR BLOCK

Q20

Have you changed your approach to public health guidelines (i.e. social distancing, face coverings, hygiene)

since you joined TestEd?

a. Yes
b. No [skip to Q24]
c. I don't know [skip to Q24]

Q21

Can you tell us about how your approach to public health guidelines has changed since your joined TestEd?

[free text box]

Q27

Overall, how would you rate your experience of the TestEd programme?

a. Excellent
b. Good
c. Fair
d. Poor
e. Very poor

Q28

Is there anything else you would like to tell us about your testing experience?
[free text box]

PAGE BREAK

Bauld L, *et al. BMJ Open* 2023; 13:e065021. doi: 10.1136/bmjopen-2022-065021
