# Supplementary material for: Students’ and staffs’ views and experiences of asymptomatic testing on a university campus during the COVID-19 pandemic in Scotland: a mixed methods study
Source: BMJ Open. 2023 Mar 20;13(3):e065021. doi: 10.1136/bmjopen-2022-065021 (PMC10030276; doi:10.1136/bmjopen-2022-065021)
Supplement: Supplementary data [file bmjopen-2022-065021supp002.pdf]

Supplementary File 2: TestEd Participant Main Survey

Notes for entry online:

BLUE = The question/variable name.
RED = Skip, display, or loop logic.
GREEN = New Block
PURPLE = Forced response

- -

INTRO BLOCK

INTRODUCTION PAGE

Thank you very much for participating in TestEd!

Any feedback you are able to provide will help us to improve the system for you and for other users.

This survey is about student and staff experiences of the University of Edinburgh's COVID-19 testing project for people that are not showing any symptoms, TestEd. Before asking you to agree to take part, we'd like to remind you of what the survey involves and how the responses you provide will be used. Participation in this survey is entirely voluntary and the survey will take you about 10-15 minutes to complete.

This information can also be found in the Participant Information Sheet for TestEd, which is available at:

https://www.ed.ac.uk/files/atoms/files/participant_information_sheet_v5.0_01_september_2021.pdf

What will happen to my data?

The demographic data (age, gender, disability status, ethnicity, whether you are a student / staff, whether you live in university owned accommodation, and your department), that you provided when you joined TestEd and the dates when you provided a TestEd saliva sample will be linked to your survey responses using your TestEd barcode. Your anonymous survey data will then be imported into quantitative data analysis software for analysis by the research team. The survey data will be retained on our Sharepoint server for a minimum of 5 years after the end of the study.

Bauld L, *et al. BMJ Open* 2023; 13:e065021. doi: 10.1136/bmjopen-2022-065021

The anonymised results of this survey may be quoted in reports and academic publications produced by the study team, which will help others to learn from TestEd's experience.Your name will never be used in any of these reports or publications and they will not include any personal identifiable information about you.

At the end of this survey we ask if you would be willing to be re-contacted to participate in a follow-up interview with a researcher from the TestEd programme. This interview is also entirely voluntary. If you tell us that you are interested in taking part in an interview, your demographic data will be shared with the TestEd interviewer.

PAGE BREAK

CONSENT

Thank you to those who completed a TestEd survey in Apr-21. We really want to hear from you again. This new survey contains some of the same questions that we asked you the last time. We really appreciate you taking the time to answer these again.

In agreeing to participate in this survey, you confirm the following:

1. I confirm that I have read and understood the Participant Information Sheet for TestEd V5.0 01 September 2021.

2. I understand that my participation is voluntary and that I can ask to withdraw at any time without giving a reason and without my legal rights being affected.

3. I understand that my rights to access, change or move my information are limited once the survey form is submitted and that you will keep the information provided even if I decide to withdraw from the survey or the TestEd study at a later date.

4. I confirm that I am happy for my survey responses to be linked to my anonymised demographic data (age, gender, disability status, ethnicity, whether you are a student / staff, whether you live in university owned accommodation, and your department) and dates when I provided TestEd saliva samples collected as part of the TestEd programme.

5. I confirm that I am happy for anonymised data from this survey to be published for research purposes.

6. I understand that my anonymised data will be stored for a minimum of 5 years and may be used in future ethically approved research.

7. I agree to take part in this TestEd survey.

Should you have any further questions about this survey or any element of TestEd please

contact us via TestEd@ed.ac.uk .

By ticking this box, I agree to the above consent points.

Q12
Please rank from most to least important what you believe are the benefits of taking part in TestEd (you may drag and drop from most to least important)

______ To know own Covid-19 status in the absence of symptoms;
______ To prevent from passing on infection to other colleagues/students on campus if I am positive;
______ To prevent from passing on infection to family and friends outside the University if I am positive;
______ To contribute to scientific research on Covid-19;
______ Because other people are using TestEd, and I feel I should too.

PAGE BREAK

Q13
Are there any other benefit(s) to taking part in TestEd (optional)
[free text box]

PAGE BREAK

Q19
How much time did you take out of your day to provide a TestEd sample (i.e providing the sample and registering it on the system)?

a. 1-2 minutes
b. 2-5 minutes
c. 5-10 minutes
d. More than 10 minutes

PAGE BREAK

Q20
How convenient do you find it to provide a TestEd sample as part of your work/study schedule?

a. Very convenient [skip to Q22]
b. Convenient [skip to Q22]
c. Neutral [skip to Q23]
d. Inconvenient
e. Very inconvenient

PAGE BREAK

Q21

You have said that you find it inconvenient to provide a TestEd sample as part of your work/study schedule. Why is this? (optional)

[free text box]

PAGE BREAK

Q22

You have said that you find it convenient to provide a TestEd sample as part of your work/study schedule. Why is this? (optional)

[free text box]

PAGE BREAK

Q25

Prior to joining TestEd, how concerned were you about catching Covid-19 on campus?

a. Very concerned
b. Moderately concerned
c. Somewhat concerned
d. Slightly concerned
e. Not at all concerned [skip to Q27]

Q26

You have said that you had concerns about catching Covid-19 on campus prior to joining TestEd. Please briefly describe what were your main concerns (optional)

[free text box]

PAGE BREAK

Q27

You have said that you did not have concerns about catching Covid-19 on campus prior to joining TestEd why is this? (optional)

[free text box]

PAGE BREAK

Q28

Do you believe that the result(s) you received from Test Ed so far were accurate?

a. Yes
b. No
c. Unsure

PAGE BREAK

Q29

Why did you believe the result(s) were accurate/inaccurate?

[free text box]

PAGE BREAK

Q31

Does the availability of the TestEd programme make you feel reassured about working/studying on campus?

a. Yes
b. No
c. Unsure

- -

TEST TRUST BLOCK

Q32

Could you explain a bit more about why you felt reassured or not?

[free text box]

- -

POST-TEST ATTITUDES AND BEHAVIOUR BLOCK

Q33

Have you changed your approach to public health guidelines (i.e. social distancing, face coverings, hygiene) since you joined TestEd?

a. Yes [display 34 to Q35]
b. No [skip to Q35]
c. I don't know [skip to Q35]

Q34

Can you tell us about how your approach to public health guidelines (i.e face coverings, hygiene) has changed since your joined TestEd?

[free text box]

PAGE BREAK

Q35

Overall, how would you rate your experience of the TestEd programme?

a. Excellent
b. Good
c. Fair
d. Poor
e. Very poor

Bauld L, *et al. BMJ Open* 2023; 13:e065021. doi: 10.1136/bmjopen-2022-065021
