# Supplementary material for: Students’ and staffs’ views and experiences of asymptomatic testing on a university campus during the COVID-19 pandemic in Scotland: a mixed methods study
Source: BMJ Open. 2023 Mar 20;13(3):e065021. doi: 10.1136/bmjopen-2022-065021 (PMC10030276; doi:10.1136/bmjopen-2022-065021)
Supplement: Supplementary data [file bmjopen-2022-065021supp003.pdf]

Supplementary File 3 - Test-Ed COVID-19 Testing Project Interview Topic Guide

Round 2 Version 1.0 01 September 2021

1. Context – experience of the Covid-19 pandemic

Could you tell me a bit about your current role/work/study at the University of Edinburgh just for context? How long have you worked/studied here?

How has the Covid-19 pandemic affected your work/study at the University of Edinburgh since the beginning of 2020?

Prompt: change in working/study location/ change in routine/ contact with peers/colleagues

Prior to joining TestEd did you have any concerns about Covid-19 on campus? Why/What were these?

What kind of impact has the Covid-19 pandemic had on you personally over the past 20 months? [Follow up on leads from the answer to this question e.g. around travel to see family/friends; concerns about personal health/health of family and friends; personal experience of Covid infection prior to joining TestEd etc.]

And how would you describe the impact the pandemic is having on your life now? Do you feel that your life is back to normal and if not, what is different? Do you worry about Covid-19 in your everyday life right now? If so, in what way?

Before starting the TestEd programme, had you had any reason to get tested for Covid-19? Can you tell us about that experience?

Prompts: Why sought testing, experience of accessing a test, physical experience of undergoing testing, response to results

Before starting the TestEd programme, had you had any reason to isolate (prompt contacted by Test and Protect/ pinged by app)? Can you tell us about that experience?

Prompts: What did they find most difficult about isolating? Where they on their own? How did they get food? Was there any reason they had to leave the house?

And in terms of your personal circumstances, do you live with others at the moment?
Prompt: type of housing (i.e. for students if in halls or elsewhere, for all - living with children, older adults etc.); Do you have any caring responsibilities for others (either inside or outside your household)?

Interview Consent Script and Topic Guide Round 2 V1.0 01 September 2021

TestEd

THE UNIVERSITY of EDINBURGH

2. Experience of TestEd

I would now like to ask a few more general questions about your experience of the TestEd programme.

Could you tell me how you heard about the programme (Test-Ed)? When was this?

Could you tell me why you decided to join the testing programme? What did you see as the main benefits of the TestEd programme?

Did you have any hesitation in joining the programme - Why?

Prompt: Any concerns about privacy of data?

Did you know anyone else who was already a part of the TestEd programme when you joined? Did you discuss your decision to join the programme with them (what did you discuss)?

Can you tell us about the process you went through to be tested for the first time?

Prompt: Where did they get tested? Did they understand what was required of them? What was their physical experience of the sample collection process? Did they have concerns about privacy related to the sample booth? How did this experience compare to any other Covid-19 testing experiences you have had (lateral flow/NHS PCR?). How quickly got results? Method for receiving results straightforward?

How have you made use of the TestEd programme since that first test?

Prompt: How often do you provide a sample? How do you fit the testing into your work/study routine? Are there any reasons why you have missed a test?

Prompt: How has your use of TestEd changed over time?

Prompt: Have you ever given a TestEd sample when you had symptoms linked to Covid-19 e.g. a cough or fever?

One concern that is often expressed about asymptomatic testing is that people might not follow public health guidelines (i.e. wearing face coverings, hand and respiratory hygiene etc) if they are being regularly tested. What is your view on that, and did you feel like that at all while you've been taking part in the TestEd programme?

Prompt: If they didn't feel like that, why not. If they did feel like that, how did it affect their behaviour?

Have you been vaccinated?

Could you tell me a bit more about when you had your first and second dose and timing of those?

Has being vaccinated changed how you feel about working/studying on campus? In what ways?

Do you think you have changed your everyday behaviour or routines in any way since you were vaccinated?

Prompt: Do you feel more protected since you have been vaccinated?

Has participation in the TestEd programme made you feel safer on campus? (note for interviewer: for those who report currently working/studying on campus)

Prompt: if not, why? Did you have any specific concerns about safety on campus prior to joining TestEd?

3. Story of Positive Test Result [for participants who have tested positive]

Can you tell me about your experience of testing positive for Covid-19 with TestEd? It would be really helpful if you could take us through your experience chronologically, starting before you were tested.

Prompts: Try to find out a clear timeline of events

Did you notice any changes in how you were feeling before you got tested?

What day of the week did you get tested and what else were you doing that day? What did you do after you gave your sample?

Where were you when you received the test result? Were you with anyone else? How did you feel when you got the result?

Did you have any worries or concerns **for yourself** following your positive test result? If so, could you tell us about them?

Did you have any worries or concerns **for others** following your positive test result? If so, could you tell us about them?

Did you think the test result was accurate at this point? Why? Why not?

Do you think your vaccination status affected your response to the test result?

What was your first response to the message? What did you do next? Did you tell anyone else your result at this point?

What contact did you have with the TestEd team? Did they give you advice on what to do next? What was that advice? Did you have any trouble following it?

Did you have food in the house? Did you have any other reason you needed to leave the house that day?

Did you need to rearrange plans because of the positive test result?

When did you book your NHS test? What did you do while you were waiting for your test? Can you take us through your experience of the NHS test? e.g. where did you go to get tested? How did you get there? What was your physical experience of the test? How did you feel while you were getting the test done? What did you do while waiting for the result? When did you get the result? How did that make you feel?

Did you have lateral flow tests in the house at the point that you received the test result? When and why had you ordered these (if not already addressed in previous questions). Did you or anyone else in your household use a lateral flow test at any point after you received the TestEd result? Can you tell us about your experience of this? Did you think the result was accurate (why/why not)?

Can you tell us about your experience of self-isolation?

Prompt: Did you feel clear about the self-isolation guidelines at the point that you tested positive? Were there any guidelines you found difficult to follow? How did you organise food (can also follow up on whether they used the food voucher they would have been given)? What was your daily routine during self-isolation? Did you have any reason you needed to leave the house during that period? If in shared house how did you manage your contact with other household members? What did you find most challenging about this period? Is there any other support that might have helped you self-isolate? Who from?

Did you experience any symptoms after your positive test? Prompt: talk through any symptoms or not after the TestEd positive result and

while waiting for/after the confirmatory test

To what extent did you trust the test result from TestEd? Why? Why not?

4. Closing questions

Interview Consent Script and Topic Guide Round 2 V1.0 01 September 2021

How could the TestEd testing experience have been improved?

Do you intend to continue participating in the TestEd programme? How do you think you will use it in the future?

Has your vaccination status affected your interest in participating in the programme at all?

Would you encourage others to participate in the TestEd programme. Prompt: if yes, why, if no, why not

Is there anything else you'd like to tell us about your experience of participating in the programme?

Would you be willing to be contacted for a short follow-up interview in the future?

Thank you for taking time to take part in this interview. [ENDS]
