# Supplementary material for: Students’ and staffs’ views and experiences of asymptomatic testing on a university campus during the COVID-19 pandemic in Scotland: a mixed methods study
Source: BMJ Open. 2023 Mar 20;13(3):e065021. doi: 10.1136/bmjopen-2022-065021 (PMC10030276; doi:10.1136/bmjopen-2022-065021)
Supplement: Supplementary data [file bmjopen-2022-065021supp004.pdf]

## Supplementary File 4 – Survey Results (for both Pilot and Main Surveys) Tables

| Pilot survey | Q8 Time taken to take test? | | | | Q15 Belief in test result? | | | Q17 Does TestEd make you feel reassured? | | | Q20 Change in approach to public health guidelines? | | Q27 Experience of TestEd programme? | | | |
|---|---|---|---|---|---|---|---|---|---|---|---|---|---|---|---|---|
| Response options / Demographics | 1-2 min. | 2-5 min. | 5-10 min | >10 min | Yes | No | Unsure | Yes | No | Unsure | Yes | No | Excellent | Good | Fair | Poor |
| Total | 223(42.7) | 235(45.0) | 57(10.9) | 7(1.3) | 472(90.4) | 2(0.4) | 48(9.2) | 470(90.2) | 17(3.3) | 34(6.5) | 16(3.1) | 497(96.9) | 408(78.2) | 108)20.7) | 6(1.2) | 0(0.0) |
| Gender *n*(%) | | | | | | | | | | | | | | | | |
| Female | 151(45.6) | 140(42.3) | 37(11.2) | 3(0.9) | 300(90.6) | 1(0.3) | 30(9.1) | 300(90.9) | 8(2.4) | 22(6.7) | 9(2.8) | 317(97.2) | 252(76.1) | 75(22.7) | 4(1.2) | 0(0.0) |
| Male | 71(37.4) | 95(50.0) | 20(10.5) | 4(2.1) | 171(90.0) | 1(0.5) | 18(9.5) | 168(89.0) | 9(4.7) | 12(6.3) | 7(3.8) | 179(96.2) | 155(81.6) | 33(17.4) | 2(1.1) | 0(0.0) |
| Comparison (p-value, $\phi_c$) | FET = 0.128, $\phi_c$ = 0.103 | | | | FET = 0.942, $\phi_c$ = 0.021 | | | $\chi^2$ = 0.337, $\phi_c$ = 0.065 | | | $\chi^2$ = 0.502, $\phi_c$ = 0.030 | | FET = 0.400, $\phi_c$ = 0.059 | | | |
| Age (years) *n*(%) | | | | | | | | | | | | | | | | |
| ≤19 | 0(0.0) | 0(0.0) | 0(0.0) | 0(0.0) | 0(0.0) | 0(0.0) | 0(0.0) | 0(0.0) | 0(0.0) | 0(0.0) | 0(0.0) | 0(0.0) | 0(0.0) | 0(0.0) | 0(0.0) | 0(0.0) |
| 20-29 | 77(44.8) | 74(43.0) | 19(11.1) | 2(1.2) | 148(86.1) | 0(0.0) | 24(14.0) | 160(93.0) | 4(2.3) | 8(4.7) | 7(4.1) | 161(95.8) | 134(77.9) | 37(21.5) | 1(0.6) | 0(0.0) |
| 30-39 | 58(42.0) | 66(47.8) | 14(10.1) | 0(0.0) | 123(89.1) | 1(0.7) | 14(10.1) | 124(89.9) | 4(2.9) | 10(7.3) | 1(0.7) | 134(99.3) | 106(76.8) | 29(21.0) | 3(2.2) | 0(0.0) |
| 40-49 | 34(41.5) | 37(45.1) | 9(11.0) | 2(2.4) | 76(92.7) | 1(1.2) | 5(6.1) | 73(89.0) | 4(4.9) | 5(6.1) | 2(2.4) | 80(97.6) | 62(75.6) | 18(22.0) | 2(2.4) | 0(0.0) |
| 50-59 | 38(46.9) | 35(43.2) | 7(8.6) | 1(1.2) | 77(95.1) | 0(0.0) | 4(4.9) | 70(86.4) | 3(3.7) | 8(9.9) | 5(6.3) | 74(93.7) | 65(80.3) | 16(19.8) | 0(0.0) | 0(0.0) |
| ≥60 | 16(32.7) | 23(47.0) | 8(16.3) | 2(4.1) | 48(98.0) | 0(0.0) | 1(2.0) | 43(89.6) | 2(4.2) | 3(6.3) | 1(2.0) | 48(98.0) | 31(83.7 | 8(16.3) | 0(0.0) | 0(0.0) |
| Comparison (p-value, $\phi_c$) | $\chi^2$ = 0.672, $\phi_c$ = 0.077 | | | | FET = 0.044, $\phi_c$ = 0.114 | | | FET = 0.789, $\phi_c$ = 0.063 | | | FET = 0.159, $\phi_c$ = 0.110 | | FET = 0.825, $\phi_c$ = 0.072 | | | |
| Ethnicity *n*(%) | | | | | | | | | | | | | | | | |
| British/Irish/Other white | 203(42.8) | 213(44.9) | 51(10.8) | 7(1.5) | 432(91.1) | 2(0.4) | 40(8.4) | 424(89.6) | 16(3.4) | 33(7.0) | 11(2.4) | 456(97.6) | 369(77.9) | 100(21.1) | 5(1.1) | 0(0.0) |
| Asian/Indian/Pakistani/Bangladeshi/ Chinese/Other Asian | 12(48.0) | 10(40.0) | 3(12.0) | 0(0.0) | 21(84.0) | 0(0.0) | 4(16.0) | 23(92.0) | 1(4.0) | 1(4.0) | 2(8.7) | 21(91.3) | 19(76.0) | 5(20.0) | 1(4.0) | 0(0.0) |
| Mixed/Other ethnic/Other black/Caribbean African | 8(36.36) | 11(50.0) | 3(13.6) | 0(0.0) | 18(82.8) | 0(0.0) | 4(18.2) | 22(100.0) | 0(0.0) | 0(0.0) | 3(13.6) | 19(86.4) | 19(86.4) | 3(13.6) | 0(0.0) | 0(0.0) |
| Comparison (p-value, $\phi_c$) | FET = 0.950, $\phi_c$ = 0.038 | | | | FET = 0.264, $\phi_c$ = 0.062 | | | FET = 0.760, $\phi_c$ = 0.053 | | | $\chi^2$ = 0.010, $\phi_c$ = 0.149 | | FET = 0.499, $\phi_c$ = 0.052 | | | |
| Role in the university *n*(%) | | | | | | | | | | | | | | | | |
| Staff | 171(44.1) | 173(44.6) | 38(9.8) | 6(1.6) | 361(93.0) | 2(0.5) | 25(6.4) | 344(88.9) | 13(3.4) | 30(7.8) | 11(2.9) | 372(97.1) | 310(79.9) | 73(18.8) | 5(1.3) | 0(0.0) |
| Students | 52(38.8) | 62(46.3) | 19(14.2) | 1(0.8) | 111(82.8) | 0(0.0) | 23(17.2) | 126(94.0) | 4(3.0) | 4(3.0) | 5(3.9) | 125(96.2) | 98(73.1) | 35(26.1) | 1(0.8) | 0(0.0) |
| Comparison (p-value, $\phi_c$) | FET = 0.424, $\phi_c$ = 0.075 | | | | FET = 0.001, $\phi_c$ = 0.165 | | | FET = 0.160, $\phi_c$ = 0.085 | | | FET = 0.566, $\phi_c$ = -0.024 | | FET = 0.160, $\phi_c$ = 0.081 | | | |
| Disability *n*(%) | | | | | | | | | | | | | | | | |
| Yes | 3(37.5) | 4(50.0) | 1(12.5) | 0(0.0) | 8(100.0) | 0(0.0) | 0(0.0) | 8(100.0) | 0(0.0) | 0(0.0) | 1(12.5) | 7(87.5) | 7(87.5) | 1(12.5) | 0(0.0) | 0(0.0) |
| No | 215(42.7) | 226(44.9) | 56(11.1) | 6(1.2) | 453(90.3) | 2(0.4) | 47(9.3) | 453(90.2) | 17(3.4) | 32(6.4) | 14(2.8) | 480(97.2) | 393(78.1) | 104(20.7) | 6(1.2) | 0(0.0) |
| Comparison (p-value, $\phi_c$) | FET = 1.000, $\phi_c$ = 0.020 | | | | FET = 1.000, $\phi_c$ = 0.041 | | | FET = 1.000, $\phi_c$ = 0.041 | | | FET = 0.217, $\phi_c$ = 0.071 | | FET = 0.100, $\phi_c$ = 0.029 | | | |

FET = Fisher's exact test, $\chi^2$ = Chi Square test, $\phi_c$ = Cramer's V

| Main survey | Q12 Most important motivations for taking part in TestEd? | | | | |
|---|---|---|---|---|---|
| Response options / Demographics | To know Covid-19 status | To prevent infecting other colleagues/students on campus | To prevent infecting friends/family outside campus | To contribute to Covid-19 research | Because other people are using TestEd, so feel I should too |
| Total | 753(38.4) | 222(11.3) | 619(31.5) | 358(18.2) | 11(0.6) |
| Gender *n*(%) | | | | | |
| Female | 442(39.0) | 129(11.4) | 360(31.8) | 196(17.3) | 6(0.5) |
| Male | 298(37.5) | 87(11.0) | 247(31.1) | 157(19.8) | 5(0.6) |
| Comparison (p-value, $\phi_c$) | FET = 0.719, $\phi_c$ = 0.033 | | | | |
| Age (years) *n*(%) | | | | | |
| ≤19 | 16(39.0) | 11(26.8) | 9(22.0) | 5(12.2) | 0(0.0) |
| 20-29 | 232(40.5) | 59(10.3) | 181(31.6) | 95(16.6) | 6(1.1) |
| 30-39 | 184(40.2) | 49(10.7) | 157(34.3) | 67(14.6) | 1(0.2) |
| 40-49 | 134(35.5) | 47(12.5) | 124(32.9) | 71(18.8) | 1(0.3) |
| 50-59 | 132(35.6) | 37(10.0) | 117(31.5) | 84(22.6) | 1(0.3) |
| ≥60 | 55(38.5) | 19(13.3) | 31(21.7) | 36(25.2) | 2(1.4) |
| Comparison (p-value, $\phi_c$) | $\chi^2$ =0.005, $\phi_c$ = 0.071 | | | | |
| Ethnicity *n*(%) | | | | | |
| British/Irish/Other white | 678(38.4) | 196(11.1) | 563(31.8) | 324(18.3) | 7(0.4) |
| Asian/Indian/Pakistani/Bangladeshi/ Chinese/Other Asian | 46(43.8) | 11(10.5) | 28(26.7) | 17(16.2) | 3(2.9) |
| Mixed/Other ethnic/Other black/Caribbean African | 25(32.1) | 12(15.4) | 24(30.8) | 16(20.5) | 1(1.3) |
| Comparison (p-value, $\phi_c$) | FET = 0.092, $\phi_c$ = 0.063 | | | | |
| Role in the university *n*(%) | | | | | |
| Staff | 538(37.8) | 153(10.8) | 460(32.4) | 264(18.6) | 7(0.5) |
| Students | 208(40.0) | 67(12.9) | 151(29.0) | 90(17.3) | 4(0.8) |
| Comparison (p-value, $\phi_c$) | FET = 0.361, $\phi_c$ = 0.046 | | | | |
| Disability *n*(%) | | | | | |
| Yes | 22(43.1) | 5(9.8) | 14(27.5) | 10(19.6) | 0(0.0) |
| No | 710(38.3) | 204(11.0) | 593(32.0) | 334(18.0) | 11(0.6) |
| Comparison (p-value, $\phi_c$) | FET = 0.905, $\phi_c$ = 0.024 | | | | |
| Staff role *n*(%) | | | | | |
| Academic | 273(37.6) | 67(9.2) | 243(33.5) | 141(19.4) | 2(0.3) |
| Facilities and estates | 40(34.2) | 17(14.5) | 37(31.6) | 21(18.0) | 2(1.7) |
| Administration | 94(39.8) | 26(11.0) | 67(28.4) | 49(20.8) | 0(0.0) |
| IT services | 32(39.5) | 11(13.6) | 27(33.3) | 11(13.6) | 0(0.0) |
| Comparison (p-value, $\phi_c$) | $\chi^2$ = 0.231, $\phi_c$ = 0.066 | | | | |

FET = Fisher's exact test, $\chi^2$ = Chi Square test, $\phi_c$ = Cramer's V

| Main survey | Q19 Time taken to take test? | | | | Q20 Convenience to provide test? | | | | | Q25 Concern about catching Covid prior to joining TestEd? | | | | |
|---|---|---|---|---|---|---|---|---|---|---|---|---|---|---|
| Response options<br>Demographics | 1-2 min. | 2-5 min. | 5-10 min | >10 min | Very convenient | Convenient | Neutral | Inconvenient | Very inconvenient | Not at all concerned | Slightly concerned | Somewhat concerned | Moderately concerned | Very concerned |
| **Total** | 857(41.8) | 948(46.2) | 208(10.2) | 37(1.8) | 1389(67.8) | 524(25.6) | 105(5.1) | 31(1.5) | 1(0.1) | 131(6.4) | 345(16.8) | 458(22.3) | 680(33.2) | 436(21.3) |
| **Gender *n*(%)** | | | | | | | | | | | | | | |
| Female | 489(41.1) | 561(47.1) | 119(10.0) | 21(1.8) | 818 (68.7) | 291(24.5) | 60(5.0) | 20(1.7) | 1(0.1) | 57(6.9) | 149(18.1) | 191(23.2) | 270(32.8) | 157(19.1) |
| Male | 356(43.2) | 369(44.8) | 85(10.3) | 14(1.7) | 558(67.7) | 214 (26.0) | 43(5.0) | 9(1.1) | 0(0.0) | 71(6.0) | 190(16.0) | 257(21.6) | 402(33.8) | 270(22.7) |
| Comparison (p-value, $\phi_c$) | $\chi^2$ = 0.825, $\phi_c$ = 0.022 | | | | FET = 0.732, $\phi_c$ = 0.035 | | | | | $\chi^2$ = 0.470, $\phi_c$ = 0.043 | | | | |
| **Age (years) *n*(%)** | | | | | | | | | | | | | | |
| ≤19 | 23(56.1) | 16(39.0) | 2(4.9) | 0(0.0) | 19(46.3) | 15(36.6) | 7(17.1) | 0(0.0) | 0(0.0) | 4(9.8) | 9(22.0) | 9(22.0) | 14(34.2) | 5(12.2) |
| 20-29 | 269(45.7) | 266(45.2) | 49(8.3) | 5(0.9) | 360(61.1) | 186(31.6) | 31(5.3) | 12(2.0) | 0(0.0) | 30(5.1) | 94(16.0) | 160(27.2) | 202(34.3) | 103(17.5) |
| 30-39 | 191(40.1) | 222(46.6) | 56(11.8) | 7(1.5) | 305(64.1) | 131(27.5) | 30(6.3) | 10(2.1) | 0(0.0) | 27(5.7) | 72(15.1) | 101(21.2) | 165(34.7) | 111(23.3) |
| 40-49 | 152(39.0) | 193(49.5) | 38(9.7) | 7(1.8) | 282(72.3) | 88(22.6) | 16(4.1) | 4(1.0) | 0(0.0) | 340(7.7) | 74(19.0) | 82(21.0) | 110(28.2) | 94(24.1) |
| 50-59 | 166(42.0) | 177(44.8) | 42(10.6) | 10(2.5) | 298(75.4) | 79(20.0) | 14(3.5) | 31(0.8) | 1(0.3) | 28(7.1) | 65(16.5) | 85(21.5) | 132(33.4) | 85(21.5) |
| ≥60 | 56(35.2) | 74(46.5) | 21(13.2) | 8(5.0) | 125(78.6) | 25(15.7) | 7(4.4) | 2(1.3) | 0(0.0) | 12(7.6) | 31(19.5) | 21(13.2) | 57(35.9) | 38(23.9) |
| Comparison (p-value, $\phi_c$) | $\chi^2$ = 0.032, $\phi_c$ = 0.068 | | | | $\chi^2$ <0.001, $\phi_c$ = 0.088 | | | | | $\chi^2$ = 0.032, $\phi_c$ = 0.065 | | | | |
| **Ethnicity *n*(%)** | | | | | | | | | | | | | | |
| British/Irish/Other white | 774(42.0) | 852(46.2) | 186(10.1) | 33(1.8) | 1262(68.4) | 462(25.0) | 93(5.0) | 28(1.5) | 1(0.1) | 124(6.7) | 320(17.3) | 407(22.1) | 622(33.7) | 372(20.2) |
| Asian/Indian/Pakistani/Bangladeshi/ Chinese/Other Asian | 50(45.1) | 48(43.2) | 12(10.8) | 1(0.9) | 61(55.0) | 39(35.1) | 9(8.1) | 2(1.8) | 0(0.0) | 2(1.8) | 12(10.8) | 31(27.9) | 36(32.4) | 30(27.0) |
| Mixed/Other ethnic/Other black/Caribbean African | 27(32.9) | 44(53.7) | 9(11.0) | 2(2.4) | 62(75.6) | 17(20.7) | 2(2.4) | 1(1.2) | 0(0.0) | 5(6.1) | 13(15.9) | 15(18.3) | 21(25.6) | 28(34.2) |
| Comparison (p-value, $\phi_c$) | FET =0.692, $\phi_c$ = 0.031 | | | | FET =0.093, $\phi_c$ = 0.055 | | | | | $\chi^2$ = 0.001, $\phi_c$ = 0.081 | | | | |
| **Role in the university *n*(%)** | | | | | | | | | | | | | | |
| Staff | 599(40.0) | 695(46.5) | 170(11.4) | 31(2.1) | 1055(70.6) | 348(23.3) | 68(4.6) | 23(1.5) | 1(0.1) | 97(6.5) | 259(17.3) | 310(20.7) | 498(33.3) | 331(22.1) |
| Students | 242(45.3) | 248(46.4) | 38(7.1) | 6(1.1) | 316(59.2) | 173(32.4) | 37(7.0) | 8(1.5) | 0(0.0) | 31(5.8) | 79(14.8) | 143(26.8) | 179(33.5) | 102(19.1) |
| Comparison (p-value, $\phi_c$) | $\chi^2$ = 0.019, $\phi_c$ = 0.072 | | | | FET<0.001, $\phi_c$ = 0.112 | | | | | $\chi^2$ = 0.033, $\phi_c$ = 0.074 | | | | |
| **Disability *n*(%)** | | | | | | | | | | | | | | |
| Yes | 19(36.5) | 26(50.0) | 5(9.6) | 2(3.9) | 35(67.3) | 11(21.2) | 5(9.6) | 1(2.0) | 0(0.0) | 0(0.0) | 11(21.2) | 13(25.0) | 14(26.9) | 14(26.9) |
| No | 817(42.2) | 889(45.9) | 196(10.1) | 33(1.7) | 1318(68.1) | 492(25.4) | 96(5.0) | 28(1.5) | 1(0.1) | 129(6.7) | 327(16.9) | 434(22.4) | 646(33.4) | 399(20.6) |
| Comparison (p-value, $\phi_c$) | FET = 0.409, $\phi_c$ = 0.037 | | | | FET = 0.561, $\phi_c$ = 0.024 | | | | | FET = 0.180, $\phi_c$ = 0.058 | | | | |
| **Staff role *n*(%)** | | | | | | | | | | | | | | |
| Academic | 308(40.8) | 355(47.0) | 77(10.2) | 15(2.0) | 512(67.8) | 194(25.7) | 37(4.9) | 12(1.6) | 0(0.0) | 54(7.2) | 130(17.2) | 171(22.7) | 260(34.4) | 140(18.5) |
| Facilities and estates | 57(41.1) | 65(47.1) | 14(10.1) | 2(1.5) | 112(81.2) | 21(15.2) | 4(2.9) | 0(0.0) | 1(0.7) | 3(2.2) | 21(15.2) | 21(15.2) | 43(31.2) | 50(36.2) |
| Administration | 85(34.3) | 119(48.0) | 37(14.9) | 7(2.8) | 184(74.2) | 52(21.0) | 7(2.8) | 5(2.0) | 0(0.0) | 17(6.9) | 51(20.6) | 44(17.7) | 73(29.4) | 63(25.4) |
| IT services | 36(43.9) | 35(42.7) | 10(12.2) | 1(1.2) | 56(68.3) | 19(23.2) | 6(7.3) | 1(1.2) | 0(0.0) | 7(8.5) | 13(15.9) | 22(26.8) | 26(31.7) | 14(17.1) |
| Comparison (p-value, $\phi_c$) | $\chi^2$ = 0.586, $\phi_c$ = 0.046 | | | | $\chi^2$ = 0.023, $\phi_c$ = 0.082 | | | | | $\chi^2$ = 0.004, $\phi_c$ = 0.913 | | | | |

FET = Fisher's exact test, $\chi^2$ = Chi Square test, $\phi_c$ = Cramer's V

| Main survey | Q28 Belief in test result? | | | Q31 Does TestEd make you feel reassured? | | | Q33 Change in approach to public health guidelines? | | | Q35 Experience of TestEd programme? | | | |
|---|---|---|---|---|---|---|---|---|---|---|---|---|---|
| Response options / Demographics | Yes | No | Unsure | Yes | No | Unsure | Yes | No | I don't know | Excellent | Good | Fair | Poor |
| **Total** | 1892(92.3) | 4(0.2) | 154(7.5) | 1787(87.2) | 99(4.8) | 164(8.0) | 94(4.6) | 1922(93.3) | 44(2.2) | 1521(74.2) | 500(24.4) | 28(1.4) | 1(0.1) |
| **Gender** ***n*****(%)** | | | | | | | | | | | | | |
| Female | 1118(92.3) | 1(0.1) | 71(6.0) | 1048(88.1) | 46(3.9) | 96(3.9) | 49(4.1) | 1117(93.9) | 24(2.0) | 889(74.7) | 288(24.2) | 12(1.0) | 1(0.1) |
| Male | 741(89.9) | 2(0.2) | 81(9.8) | 708(85.9) | 53(6.4) | 63(7.7) | 44(5.4) | 761(92.5) | 18(2.2) | 611(74.2) | 199(24.2) | 14(1.7) | 0(0.0) |
| Comparison (p-value, $\phi_c$) | FET = 0.003, $\phi_c$ = 0.0735 | | | $\chi^2$ = 0.029, $\phi_c$ = 0.061 | | | $\chi^2$ = 0.244, $\phi_c$ = 0.038 | | | $\chi^2$ = 0.470, $\phi_c$ = 0.043 | | | |
| **Age (years)** ***n*****(%)** | | | | | | | | | | | | | |
| ≤19 | 39(95.1) | 0(0.0) | 2(4.9) | 36(87.8) | 0(0.0) | 5(12.2) | 2(5.0) | 36(90.0) | 2(5.0) | 22(52.7) | 17(41.5) | 2(4.9) | 0(0.0) |
| 20-29 | 536(91.0) | 2(0.3) | 51(8.7) | 528(89.6) | 22(3.7) | 39(6.6) | 29(4.9) | 539(91.5) | 21(3.6) | 398(67.6) | 179(30.4) | 11(1.9) | 1(0.2) |
| 30-39 | 437(91.8) | 0(0.0) | 39(8.2) | 406(85.3) | 28(5.9) | 42(8.8) | 25(5.2) | 441(92.7) | 10(2.1) | 328(68.9) | 140(29.4) | 8(1.7) | 0(0.0) |
| 40-49 | 362(92.8) | 1(0.3) | 27(7.0) | 340(87.2) | 22(5.6) | 28(7.2) | 15(3.9) | 372(95.4) | 3(0.8) | 308(79.0) | 80(20.5) | 2(0.5) | 0(0.0) |
| 50-59 | 366(92.7) | 1(0.3) | 28(7.1) | 338(85.6) | 19(4.8) | 38(9.6) | 7(1.8) | 383(97.0) | 5(1.3) | 333(84.3) | 61(15.4) | 1(0.3) | 0(0.0) |
| ≥60 | 152(95.6) | 0(0.0) | 7(4.4) | 139(87.4) | 8(5.0) | 12(7.6) | 16(10.1) | 140(88.1) | 3(1.9) | 132(83.0) | 23(14.5) | 4(2.5) | 0(0.0) |
| Comparison (p-value, $\phi_c$) | FET = 0.770, $\phi_c$ = 0.038 | | | $\chi^2$ = 0.326, $\phi_c$ = 0.054 | | | $\chi^2$ <0.001, $\phi_c$ = 0.092 | | | $\chi^2$ <0.001, $\phi_c$ = 0.126 | | | |
| **Ethnicity** ***n*****(%)** | | | | | | | | | | | | | |
| British/Irish/Other white | 1711(92.7) | 3(0.2) | 131(7.1) | 1604(86.9) | 89(4.8) | 152(8.2) | 80(4.3) | 1729(93.8) | 35(1.9) | 1390(75.3) | 429(23.3) | 25(1.4) | 1(0.1) |
| Asian/Indian/Pakistani/Bangladeshi/ Chinese/Other Asian | 94(84.7) | 0(0.0) | 17(15.3) | 102(92.0) | 3(2.7) | 6(5.4) | 8(7.2) | 96(86.5) | 7(6.3) | 70(63.1) | 41(37.0) | 0(0.0) | 0(0.0) |
| Mixed/Other ethnic/Other black/Caribbean African | 75(91.5) | 1(1.2) | 6(7.3) | 71(86.6) | 6(7.3) | 5(6.1) | 5(6.1) | 75(91.5) | 2(2.4) | 51(62.2) | 28(34.2) | 3(3.7) | 0(0.0) |
| Comparison (p-value, $\phi_c$) | FET = 0.005, $\phi_c$ = 0.069 | | | FET = 0.296, $\phi_c$ = 0.037 | | | FET = 0.058, $\phi_c$ = 0.048 | | | FET =0.001, $\phi_c$ = 0.070 | | | |
| **Role in the university** ***n*****(%)** | | | | | | | | | | | | | |
| Staff | 1382(92.4) | 2(0.1) | 111(7.4) | 1293(86.5) | 78(5.2) | 124(8.3) | 67(4.5) | 1398(93.5) | 30(2.0) | 1162(77.7) | 320(21.4) | 13(0.9) | 0(0.0) |
| Students | 491(92.0) | 2(0.4) | 41(7.7) | 474(88.8) | 21(3.9) | 39(7.3) | 27(5.1) | 492(92.3) | 14(2.6) | 341(63.9) | 177(33.2) | 15(2.8) | 1(0.2) |
| Comparison (p-value, $\phi_c$) | FET = 1.000, $\phi_c$ = 0.006 | | | $\chi^2$ = 0.188, $\phi_c$ = 0.042 | | | $\chi^2$ = 0.627, $\phi_c$ = 0.022 | | | $\chi^2$ <0.001, $\phi_c$ = 0.150 | | | |
| **Disability** ***n*****(%)** | | | | | | | | | | | | | |
| Yes | 51(98.1) | 0(0.0) | 1(1.9) | 47(90.4) | 1(1.9) | 4(7.7) | 4(7.7) | 46(88.5) | 2(3.9) | 34(65.4) | 18(34.6) | 0(0.0) | 0(0.0) |
| No | 1782(92.1) | 4(0.2) | 149(7.7) | 1690(87.3) | 94(4.9) | 151(7.8) | 86(4.5) | 1810(93.6) | 38(2.0) | 1449(74.9) | 460(23.8) | 25(1.3) | 1(0.1) |
| Comparison (p-value, $\phi_c$) | FET = 0.241, $\phi_c$ = 0.036 | | | FET = 0.757, $\phi_c$ = 0.022 | | | FET = 0.175, $\phi_c$ = 0.038 | | | FET = 0.130, $\phi_c$ = 0.049 | | | |
| **Staff role** ***n*****(%)** | | | | | | | | | | | | | |
| Academic | 690(91.4) | 1(0.1) | 64(8.5) | 657(87.0) | 41(5.4) | 57(7.6) | 22(2.9) | 721(95.5) | 12(1.6) | 593(78.5) | 157(20.8) | 5(0.7) | 0.(0.0) |
| Facilities and estates | 132(95.7) | 1(0.7) | 5(3.6) | 121(87.7) | 5(3.6) | 12(8.7) | 19(13.8) | 113(81.9) | 6(4.4) | 111(80.4) | 26(18.8) | 1(0.7) | 0.(0.0) |
| Administration | 230(92.7) | 0(0.0) | 18(7.3) | 215(86.7) | 9(3.6) | 24(9.7) | 11(4.4) | 235(94.8) | 2(0.8) | 193(77.8) | 54(21.8) | 1(0.4) | 0.(0.0) |
| IT services | 76(92.7) | 0(0.0) | 6(7.3) | 63(76.8) | 7(8.5) | 12(14.6) | 1(1.2) | 78(95.1) | 3(3.7) | 61(74.4) | 20(24.4) | 1(1.2) | 0.(0.0) |
| Comparison (p-value, $\phi_c$) | FET = 0.308, $\phi_c$ = 0.054 | | | FET = 0.105, $\phi_c$ = 0.067 | | | FET <0.001, $\phi_c$ = 0.137 | | | FET = 0.712, $\phi_c$ = 0.034 | | | |

FET = Fisher's exact test, $\chi^2$ = Chi Square test, $\phi_c$ = Cramer's V
