# Supplementary material for: Students’ and staffs’ views and experiences of asymptomatic testing on a university campus during the COVID-19 pandemic in Scotland: a mixed methods study
Source: BMJ Open. 2023 Mar 20;13(3):e065021. doi: 10.1136/bmjopen-2022-065021 (PMC10030276; doi:10.1136/bmjopen-2022-065021)
Supplement: Author's manuscript [file bmjopen-2022-065021.draft_revisions.pdf]

BMJ Open is committed to open peer review. As part of this commitment we make the peer review history of every article we publish publicly available.

When an article is published we post the peer reviewers' comments and the authors' responses online. We also post the versions of the paper that were used during peer review. These are the versions that the peer review comments apply to.

The versions of the paper that follow are the versions that were submitted during the peer review process. They are not the versions of record or the final published versions. They should not be cited or distributed as the published version of this manuscript.

BMJ Open is an open access journal and the full, final, typeset and author-corrected version of record of the manuscript is available on our site with no access controls, subscription charges or pay-per-view fees (http://bmjopen.bmj.com).

If you have any questions on BMJ Open's open peer review process please email info.bmjopen@bmj.com

BMJ Open

Student and staff views and experiences of asymptomatic testing on a university campus during the Covid-19 pandemic in Scotland: The TestEd study

Journal:	*BMJ Open*
Manuscript ID	bmjopen-2022-065021
Article Type:	Original research
Date Submitted by the Author:	24-May-2022

Complete List of Authors:	Bauld, Linda; The University of Edinburgh Usher Institute of Population Health Sciences and Informatics<br>Street, Alice; The University of Edinburgh School of Social and Political Science,<br>Connelly, Roxanne; The University of Edinburgh School of Social and Political Science<br>Bevan, Imogen; The University of Edinburgh School of Social and Political Science<br>Morlet Corti, Yazmin; The University of Edinburgh School of Social and Political Science<br>Baxter, Mats; The University of Edinburgh, Usher Institute<br>Stagg, Helen R.; The University of Edinburgh Usher Institute of Population Health Sciences and Informatics<br>Christison, Sarah; The University of Edinburgh School of Social and Political Science<br>Mulherin, Tamara; The University of Edinburgh Usher Institute of Population Health Sciences and Informatics<br>Sinclair, Lesley; University of York, Department of Health Sciences<br>Aitman, Tim; The University of Edinburgh MRC Institute of Genetics and Molecular Medicine, Institute of Genetics and Cancer

Keywords:	PUBLIC HEALTH, COVID-19, Health policy < HEALTH SERVICES ADMINISTRATION & MANAGEMENT, Infection control < INFECTIOUS DISEASES, QUALITATIVE RESEARCH

SCHOLARONE™
Manuscripts

I, the Submitting Author has the right to grant and does grant on behalf of all authors of the Work (as defined in the below author licence), an exclusive licence and/or a non-exclusive licence for contributions from authors who are: i) UK Crown employees; ii) where BMJ has agreed a CC-BY licence shall apply, and/or iii) in accordance with the terms applicable for US Federal Government officers or employees acting as part of their official duties; on a worldwide, perpetual, irrevocable, royalty-free basis to BMJ Publishing Group Ltd ("BMJ") its licensees and where the relevant Journal is co-owned by BMJ to the co-owners of the Journal, to publish the Work in this journal and any other BMJ products and to exploit all rights, as set out in our licence.

The Submitting Author accepts and understands that any supply made under these terms is made by BMJ to the Submitting Author unless you are acting as an employee on behalf of your employer or a postgraduate student of an affiliated institution which is paying any applicable article publishing charge ("APC") for Open Access articles. Where the Submitting Author wishes to make the Work available on an Open Access basis (and intends to pay the relevant APC), the terms of reuse of such Open Access shall be governed by a Creative Commons licence – details of these licences and which Creative Commons licence will apply to this Work are set out in our licence referred to above.

Other than as permitted in any relevant BMJ Author's Self Archiving Policies, I confirm this Work has not been accepted for publication elsewhere, is not being considered for publication elsewhere and does not duplicate material already published. I confirm all authors consent to publication of this Work and authorise the granting of this licence.

For peer review only

Title	**Student and staff views and experiences of asymptomatic testing on a university campus during the Covid-19 pandemic in Scotland: The TestEd study**
Author's names	Linda Bauld[1]<br>Alice Street[2]<br>Roxanne Connelly[2]<br>Imogen Bevan[1,2]<br>Yazmin Morlet Corti[1,2]<br>Mats Stage Baxter[1]<br>Helen R. Stagg[1]<br>Sarah Christison[2]<br>Tamara Mulherin[1]<br>Lesley Sinclair[3]<br>Tim Aitman[4]
Address for each author<br>Authors names and positions	[1]Usher Institute, College of Medicine and Veterinary Medicine, University of Edinburgh<br>[2]School of Social and Political Science, College of Arts and Humanities, University of Edinburgh<br>[3]Department of Health Sciences, University of York<br>[4] Institute of Genetics and Cancer, University of Edinburgh
Corresponding Author	Correspondence to: Linda.Bauld@ed.ac.uk<br>Usher Institute – University of Edinburgh<br>Doorway 1, Old Medical School<br>Teviot Place<br>Edinburgh EH8 9AG<br>Linda.Bauld@ed.ac.uk
Word Count	4600

For peer review only - http://bmjopen.bmj.com/site/about/guidelines.xhtml

Abstract

Objectives - To explore the acceptability of regular asymptomatic testing for SARS-CoV-2 on a university campus using saliva sampling for polymerase chain reaction (PCR) analysis, and the barriers and facilitators to participation.

Design - Cross-sectional surveys and qualitative semi-structured interviews.

Setting - City of Edinburgh, Scotland.

Participants - University staff and students who had registered for the testing programme (TestEd) and provided at least one sample.

Results - 522 participants completed a pilot survey in April 2021 and 1,750 completed the main survey (November 2021). 48 staff and students who consented to be contacted for interview took part in the qualitative research. Participants were positive about their experience with TestEd with 94% describing it as 'excellent' or 'good'. Facilitators to participation included multiple testing sites on campus, ease of providing saliva samples compared to nasopharyngeal swabs, perceived accuracy compared to lateral flow devices (LFDs) and reassurance of test availability while working or studying on campus. Barriers included concerns about privacy while testing, time to and methods of receiving results compared to LFDs, and concerns about insufficient uptake in the university community. There was little evidence that availability of testing on campus changed the behaviour of participants during a period when Covid-19 restrictions were in place.

Conclusions - Provision of free asymptomatic testing for Covid-19 on a university campus was welcomed by participants and the use of saliva-based PCR testing was regarded as more comfortable and accurate than LFDs. Convenience is a key facilitator of participation in regular asymptomatic testing programmes. Availability of testing did not appear to undermine engagement with public health guidelines.

Strengths and limitations of this study

- First study to explore perceptions and experiences of a novel saliva-based PCR asymptomatic testing programme for Covid-19 that is designed to improve on LFDs in a screening context.
- Mixed methods research including two surveys six months apart and in-depth semi-structured interviews with a subsample of participants.
- Limited to the views and experiences of those who chose to take part in a voluntary testing programme and could not explore reasons for non-participation or compare the characteristics of participants with the university population as a whole.
- Findings may be transferable to other asymptomatic testing programmes for SARS-CoV-2 or other viruses on university campuses or in other educational settings and workplaces.

For peer review only - http://bmjopen.bmj.com/site/about/guidelines.xhtml

Introduction

The extent to which universities played a role in community transmission of SARS-CoV-2 was heavily debated in the UK in the early stages of the COVID-19 pandemic.[1,2] As in many other countries, higher and further education institutions had to pause non-essential teaching and research activities on several occasions, leading to long periods of online learning and many staff working from home. Essential campus activities continued throughout, however, and students moved between their term-time accommodation and other locations. In order to improve the safety of on-campus activities and reduce the risks of outbreaks, some experts recommended regular asymptomatic testing of students and staff alongside other public health measures.[3–5]

A few UK universities were early adopters of this approach, establishing their own pilot asymptomatic testing programmes involving either polymerase chain reaction (PCR)[6,7] or lateral flow devices (LFDs).[8,9] Early studies of these programmes found acceptability of asymptomatic testing among students to be high. However, uptake and adherence were found to be affected by anxiety[6] and concerns about the accuracy of tests, especially LFDs,[8,9] raising questions about students' long-term willingness to engage with regular testing. Government-funded asymptomatic COVID-19 testing in the form of LFDs first became available to all UK universities in December 2020 following concerns that a mass 'migration' of students over the winter break might lead to a rapid rise in cases.[10] This was offered to all students leaving and returning to campus. Evaluations of this testing programme found uptake among students to be low[11] and that concerns about accuracy were a prominent barrier to participation.[11,12] LFDs were rolled out to the general public from April 2021. Students and staff were then encouraged to test twice a week using LFDs. However, given their low sensitivity, several experts have queried the benefits and cost-effectiveness of mass asymptomatic LFD testing, especially during periods of lower viral prevalence in the community.[13–15]

The University of Edinburgh established an asymptomatic testing research programme, TestEd (www.ed.ac.uk/tested-covid), in January 2021. This aimed to improve on existing approaches to PCR testing in terms of acceptability and cost, and also provide a more accurate alternative to LFDs. TestEd involves a novel testing platform that uses pooled saliva-based testing by PCR, with a protocol adapted from an approach for nasopharyngeal swab testing.[16]

TestEd included surveys and interviews with participating staff and students to explore: the acceptability of regular PCR testing among students and staff, particularly involving an approach that was less invasive than nasopharyngeal swabbing; barriers and facilitators to participating in a regular university testing programme, including in the context of other testing methods being available; and whether participation in such a programme changed adherence to public health guidelines. We suggest that understanding staff and students' perceptions and experiences of TestEd's novel testing system can help to inform the design of effective regular asymptomatic testing programmes for Covid-19 or other disease outbreaks in educational and workplace settings in the future.

Methods

The TestEd programme

For peer review only - http://bmjopen.bmj.com/site/about/guidelines.xhtml

All University of Edinburgh students and staff were eligible to take part in TestEd on a voluntary basis and could sign up at any time. After joining, they were encouraged to provide twice-weekly saliva samples at one of the thirty testing centres located throughout the university. This involved spitting into a plastic cup, transferring the saliva to a tube and scanning their participant identifier and a barcode on the tube to register their sample. Samples were then collected from test sites and transferred to a university lab for PCR analysis. Participants normally received their test results within 24 hours by logging onto a secure portal with their university username and password. Between January 2021 and February 2022, 3,895 staff and 3,106 students registered and consented to participate. The programme tested just over 100,000 samples with more than 170 positive results during that period. A supermarket shopping voucher was provided to those who tested positive and sought a confirmatory PCR test from the NHS to assist with self-isolation. Participants were asked not to travel to campus to access testing, but instead to use TestEd while already there to study/work.

Design

Participants who consented to taking part in TestEd and who had provided at least one saliva sample were invited by email to participate in two online surveys using the Qualtrics tool, one (a pilot) carried out between 15 April and 30 April 2021 and the main survey between 8 November and 21 November 2021. The pilot and main surveys consisted of closed-ended and open-ended questions (See Supplementary File (SF) 1 and 2). No questions were compulsory. The number of eligible TestEd participants increased between the pilot and the main survey when students and more staff returned to campus for the 2021/22 academic year.

Semi-structured online interviews with participants were conducted between May 2021 and February 2022 (see Supplementary File 3). We were particularly interested in the views of participants who tested positive and aimed to interview more of this group than those who tested negative. We used purposive sampling to recruit participants from across the university and a wide range of demographic groups (university role, age, gender, ethnicity and disability) in order to ensure a diverse range of views and experiences were represented.

Patient and Public Involvement

Volunteer students and staff were involved in contributing to the survey design and testing the questionnaire before the survey launched.

Analysis

Data from both surveys were extracted from Qualtrics and exported to Stata. Variable recoding was undertaken to enable appropriate cell sizes for statistical analysis and to avoid statistical disclosure (>15). Variables indicating gender, age, ethnicity, disability and university role were recorded. Recoding as missing values was applied for all variables with ‘not applicable’ and ‘prefer not to say’ responses. Due to small numbers, the ‘non-binary’ category of gender was recoded as missing, and the categories of ethnicity were grouped as shown in Table 1. Responses to the survey questions were examined using descriptive statistics (e.g., frequencies and percentages). We conducted chi square tests and Fisher’s exact tests where appropriate in order to investigate patterns between sociodemographic

For peer review only - http://bmjopen.bmj.com/site/about/guidelines.xhtml

characteristics and responses to the survey. While some of these tests were statistically significant, effect sizes were very low (Cramér's ≤ 0.1) indicating only very weak patterns of association. These results are not presented in the main text and are available instead in a supplementary file (see Supplementary File 4). For questions that were duplicated in the two surveys, where participants had responded to these both times it was possible to analyse changes in attitudes and experiences between the two time points.

Qualitative data from open-ended survey questions and semi-structured interviews were analysed through a thematic coding approach by SC, IB and AS using NVivo software (versions 1.3 and 1.6.1). The content of the survey questions provided an initial coding structure, which was revised during analysis to reflect additional issues and topics raised in the results. Coding of semi-structured interviews was inductive, reflecting the more open-ended nature of the interviews. The interviews addressed a wide range of topics and for this article we only analysed a subset of results related to acceptability, perceptions and experiences of the TestEd programme. Initial coding was carried out by SC (survey) and IB (interviews) and quality checked by AS who read all results and interview transcripts. Coding categories were collectively reviewed, discussed and revised as a team before a final coding structure was agreed for each dataset and a common set of themes arrived at, as reflected in the subheadings below.

Results

Out of 760 eligible participants who had provided at least one saliva sample when the pilot survey was distributed, 548 responses were received (72%), 522 of which were complete (69%). For the main survey, out of 4,512 eligible participants, 2,995 responses were received (66%), 1,750 of which were complete (58%). 300 participants responded to both surveys. 70 participants were invited for interview, 48 of whom were successfully contacted and took part.

Participant characteristics

Participant characteristics are shown in Table 1. When compared to TestEd participants overall, the survey population included more staff members and participants identifying as female (data not shown).

Table 1: Participant characteristics

Participant characteristics	Main survey n (%)	Pilot n (%)	Interviews
**Overall**	**1750**	**300**	**48**
**Gender**			
Female	996 (58%)	194 (65%)	26
Male	721 (41%)	103 (34%)	21
Non-binary	21 (1%)	2 (<1%)	1
Other	1 (<1%)	0 (<1%)	0
Preferred not to disclose	33 (<1%)	3 (<1%)	0
**Age (years)**			
≤19	41 (2%)	0 (0%)	4

For peer review only - http://bmjopen.bmj.com/site/about/guidelines.xhtml

20–29	512 (29%)	77 (26%)	13
30–39	403 (23%)	73 (24%)	11
40–49	336 (19%)	54 (18%)	9
50–59	335 (19%)	60 (20%)	5
≥60	123 (7%)	36 (12%)	6
**Ethnicity**			
British/Irish/Other white	1570 (90%)	272 (92%)	33
Asian/Indian/Pakistani/Bangladeshi/Chinese/Other Asian	98 (6%)	13 (4%)	9
Mixed/Other ethnic/Other black/Caribbean African	71 (4%)	11 (4%)	6
Preferred not to disclose	11 (<1%)	1 (<1%)	0
**Role in the university**			
Staff	1247 (72%)	248 (83%)	28
Student	482 (28%)	52 (17%)	20
Preferred not to disclose	21 (<1%)	0(<1%)	0
**Disability**			
Yes	46 (3%)	6 (2%)	5
No	1651 (97%)	284 (98%)	40
Preferred not to disclose	53(<1%)	10 (<1%)	3

*Original values are retained; the analysis groups responses <15 into categories.
** Sociodemographic characteristics were collected at TestEd registration.

Reasons for participating

Overall, survey participants were positive about their experience with TestEd. 74% rated the experience as 'excellent' and 24% as 'good' in the main survey (see SF4). Those who participated in both the pilot and main surveys maintained enthusiasm for the programme over time, with little change in responses.

Survey responses indicated that 'knowing [their own] Covid-19 status in the absence of symptoms' was the most important reason for participation (38%), followed by prevention of 'passing on infection to family and friends' (32%). For 18% of respondents the most important reason was 'to contribute to scientific research on Covid-19', and for 11% this was 'to prevent passing on infection to other colleagues/students on campus if I am positive'.

Interview participants similarly emphasised their desire to protect family and friends beyond the university community as being a primary motivation for joining the programme. While knowing their own Covid-19 status was considered important, this was often linked to the benefit of protecting others inside or outside the university, rather than viewing these as separate benefits of testing. The rationale of contributing to scientific research often emerged as an additional but secondary concern for interviewees. Other factors that interviewees suggested motivated them to join TestEd included the perceived need to follow government or institutional guidance; support and encouragement from the institution to take part; influence from peers; perceptions of risk; and, in a few select cases, the experience of Covid-like symptoms.

Testing method

For peer review only - http://bmjopen.bmj.com/site/about/guidelines.xhtml

Survey participants found the simple spit test easy to administer and less invasive compared to standard PCR or LFD swab-based tests. They found the process of providing a saliva sample to be fairly quick: for 42% of respondents, it took only 2–5 minutes out of their day; 41% indicated that it took just 1–2 minutes.

However, the saliva testing was not without problems. A few participants indicated that it could be difficult to produce enough saliva to provide a viable sample. This was also raised in interviews. Staff and students who signed up to TestEd were asked not to eat or drink for 30 minutes before testing. Some survey participants described this as a limitation, indicating that they would find it more convenient to provide a sample during their lunch or coffee breaks. There were also some issues with the privacy of sample collection booths, with some people feeling uncomfortable spitting into a cup when they could be observed. The booths did have sides but were located in public venues on campus. Table 2 reports a selection of participant views on the testing method.

Table 2: Views on the TestEd testing method

Facilitators	Barriers
'It's non-invasive, simple, and involves no discomfort whatsoever. This is a huge benefit in making a testing regime attractive to its users.'<br><br>*'A much less invasive form of testing compared to lateral flow tests! Given how invasive they are, I also doubt many are correctly using other lateral flow tests, rendering the results inaccurate.'*<br><br>*'Saliva samples are very easy to provide (and non-intrusive) and I was concerned that I may not have been doing the lateral flow nasal and throat swab correctly hence my preference for saliva sampling.'*<br><br>*'It is very convenient and much more accessible than doing a tonsil/nostril swab. Saliva spit tests increase my motivation to test.'*	*'If the spit sample is not of a high enough volume it will not work. So sometimes my results may have been invalid. I have to work up spit in my mouth for a couple of minutes prior.'*<br><br>*'Sample can be given easily on the way to school. The only inconvenience comes from the time taken to collect enough saliva for the sample and finding a time where I have not eaten or drank in the past 30 minutes.'*<br><br>*'I'd prefer a privacy curtain that I could pull behind me when I'm in the booth. I feel very exposed when spitting in the cup in the middle of the library, especially if things get messy!'*<br><br>*'I felt very much under pressure to do this spitting thing, and I couldn't perform basically, so I just took everything with me in the office and I was like, "I'm nice and safe here." There was nobody around, but still it felt very weird to have to spit.'*

Convenience

The majority of survey participants also indicated that it was either 'very convenient' (68%) or 'convenient' (26%) to provide a sample as part of their work/study schedules (see SF4). Participants touched on issues of convenience at multiple points in the testing process, from experiences of sample collection, to navigating the TestEd IT systems, to the receipt of results.

Participating in TestEd was reported to be convenient due to the number and location of the

For peer review only - http://bmjopen.bmj.com/site/about/guidelines.xhtml

test centres, which were in many cases located within buildings where participants worked. Participants also described how the drop-in element made participating easier as tests could be taken at any time without appointment or prior booking. Interview responses revealed that perceptions of convenience were often linked to individual work/study patterns. Interviewees with a regular on-campus working schedule, and particularly those with a testing site inside the same building as their office or laboratory, reported developing a routine testing schedule (e.g., on the way to work or during a morning break) by comparison with students and staff with more varying schedules and who worked across multiple locations or between home and campus, who found it harder to establish a testing routine.

Some expressed a desire for longer opening hours, better communication of opening hours, or complained that some centres were not open as advertised or could not be accessed without the correct security clearance on their staff/student card. Many participants also found the testing programme's IT systems cumbersome. In a few cases people reported that the bar code on the test tube did not work. Many participants described the process for logging in and accessing results through the online system to be inconvenient and expressed a preference for the NHS system of sending results directly via SMS and email. While some found the turnaround times to be 'quick', other participants described turnaround times to be inconsistent or too long. Further probing in interviews revealed that perceptions of test turnaround times as either quick or slow were often shaped by comparison with another form of testing (e.g., LFD, NHS-administered PCR test), and by specific time-sensitive motivations for testing on that occasion. Table 3 presents some of these views about perceived convenience.

Table 3: Convenience of TestEd

Facilitators	Barriers
'There is a testing station at my university accommodation so it is very easy to get to and provide a sample.'<br><br>*'The booths are close to my work area. The process is quick, so you can easily fit in your schedule. Also it's self-administered and open all the time, so you can test anytime.'*<br><br>*'Station is set up throughout working hours, drop-in nature means can give a sample at a time that suits in my clinical day.'*	*'I'm either out on site so I'm at [campus site 1], or I'm at [site 2] – it then becomes a question of, "Do I have the time to drive from those locations back to [site 3] for a ten-minute spit test?" So at times you just have to sacrifice the test and not go.'*<br><br>*'It seems unnecessary to have to log in to get my results once notified. The NHS system doesn't require this: the text message and email both contain the test result.'*<br><br>*'Sometimes the results take longer to come through than other times – it can be hard to know how long to expect to wait for results.'*

Concerns about Covid-19 on campus

For peer review only - http://bmjopen.bmj.com/site/about/guidelines.xhtml

Most survey participants indicated some level of concern about catching Covid-19 on campus: 21% were ‘very concerned’; 33% were ‘moderately concerned’; and 22% ‘somewhat concerned’. Many expressed concerns about the return of students and the re-introduction of in-person teaching, which were perceived to have led to increased mixing on campus. A common concern was the lack of adequate ventilation in teaching rooms and the ability to maintain social distancing in shared spaces:

‘I slightly worry that I may catch Covid-19 from a student in class, as I spend a good amount of time with my students and not all our rooms are as well ventilated as I'd like them to be.’

‘There is obviously some increased risk due to meeting more people and using more shared facilities than if working at home.’

Despite these concerns, many survey participants perceived the likelihood of infection on campus to be lower than elsewhere. While some felt that there was low compliance with safety measures, others believed that the university’s infection control measures were robust and effective. Some of these different perspectives of safety on campus may be related to a participant’s position or role within the university – for example, working alone in single-occupancy offices vs roles that involved more contact with others at work or while studying:

‘I felt that the safety precautions in operation at work (mask-wearing, handwashing, social distancing) were adequate.’

‘[I am] usually based in my office which is single occupancy – risk here is less than going to the shops.’

Reassurance

The majority of survey participants (87%) indicated that the availability of the TestEd programme made them feel reassured about working or studying on campus. Levels of reassurance increased over time among participants who took part in both the pilot and main surveys, rising from 90% to 94%.

In some cases, TestEd provided reassurance about participants’ own health, but it was more common for participants to connect that reassurance to their sense of personal responsibility for the wellbeing of others. Responses to open-ended survey questions and interviews indicated that perceived levels of participation among others influenced how reassured individuals felt. Those who felt that there were high levels of participation, particularly among close colleagues, indicated that this made them feel reassured, while those who thought those around them on campus were not taking part had more concerns. One factor that influenced how participants perceived participation was the number of samples which they noticed had been provided at test sites. Concerns about low participation led some participants and interviewees to reflect on the efficacy of a workplace testing programme that relied on voluntary participation. Table 4 highlights a number of these responses.

Table 4: Reassurance

Facilitators	Barriers
‘[I] am severely immunocompromised so worried about all contact. Knowing the	*‘It's unclear to me how many staff and students are taking part and how*

For peer review only - http://bmjopen.bmj.com/site/about/guidelines.xhtml

majority are being tested regularly has eased these concerns.'<br><br>*'I don't think it makes a significant difference to my risk of catching Covid on campus, but it reduces the risk that I might unknowingly pass on Covid.'*<br><br>*'It's good that my colleagues and I have access to a free and accurate testing service, so I am confident that I am not unwittingly spreading COVID.'*<br><br>*'Most of my direct colleagues are using TestEd as well. Reassuring when working in the same room.'*<br><br>*'Knowing that colleagues were also participating in the programme provided a certain level of reassurance, along with my own results of course.'*<br><br>*'Because as the staffing levels have increased, I see an increasing amount of provided samples in the collection trays so I am confident people are getting regularly tested.'*	*regular[ly] they are testing, so it doesn't necessarily make me feel more reassured about catching Covid-19 while at work.'*<br>*'On one hand it is definitely a positive, but on the other I often see how few samples have been submitted when I go to drop off my own. It doesn't seem like as many people have taken advantage of the availability of the system as could have.'*<br><br>*'There seems to be very little take up on it – maybe only 15–20 samples when I go so [I am] concerned a lot of people, especially students, aren't doing it. I'm aware of outbreaks on campus but we're not officially informed of that – I think we should be.'*<br><br>*'If everyone on campus was required to enrol in TestEd to work/study on campus, I would feel safer. Voluntary enrolment is not good enough to ensure safety.'*<br><br>*'I'd feel more assured if it was compulsory for all who use campus. Some of my students think they are immune and are less risk-averse as a result.'*

Accuracy

The vast majority of survey participants (92%) agreed that the results they received from TestEd were accurate. Among those who completed both the pilot and the main survey, 93% indicated in both surveys that they believed their results from TestEd were accurate.

In open-ended survey responses and interviews, participants emphasised their trust in the scientists involved in developing the TestEd programme as a basis for their belief in the accuracy of the test results. Participants also described how they had more faith in the PCR testing used for TestEd compared to LFDs. Some also reported that they felt that the saliva-based tests were likely to be more accurate, as the sample collection process was less prone to user error compared with self-administered swabs.

While participating in TestEd, many people were also using other testing methods, most commonly LFDs that were freely available in a variety of venues, including on campus. In the case of a positive TestEd result, all participants interviewed carried out a confirmatory PCR test through the NHS so that a positive test picked up in the study could be formally reported, allowing for contact tracing by the NHS. Testing positive via this confirmatory NHS test also confirmed for many that TestEd's methods were accurate. Interviewees also

For peer review only - http://bmjopen.bmj.com/site/about/guidelines.xhtml

reported using LFDs either to confirm a positive TestEd result, or to check the accuracy of LFDs compared to PCR.

For the small proportion of participants who were unsure about the accuracy of TestEd results, open-ended responses indicated that more information regarding the effectiveness of saliva-based testing could provide reassurance. Some of this concern over accuracy was linked to the novel nature of the approach, with several participants stating that they felt there was a lack of knowledge regarding the effectiveness of saliva-based testing, or that the programme was an experimental study to trial this type of testing methodology (see Table 5).

Table 5: Accuracy

Facilitators	Barriers
'Because I trust the science behind it and I don't believe that it would have been rolled out university-wide if the university and the people behind TestEd were not confident that it would work.'<br><br>*'I understand TestEd used a PCR test which the NHS says is more accurate than a lateral flow test.'*<br><br>*'The quality of the sample provided is independently verified by the TestEd research team. Providing a saliva sample is also more straightforward and likely more error-free.'*<br><br>*'I mean the PCR test from the NHS was positive as well so I'm pretty sure it [TestEd] was [accurate]. With not having any symptoms, and then I got a positive, I might have been, "Oh, I'm not 100% sure." But having both tests positive, I'm pretty sure it has been accurate.'*	*'I am unsure about the effectiveness of the saliva as compared to the nasal swab, and have not seen data to show that. I also don't know if there are therefore not a lot of false negatives.'*<br><br>*'Haven't heard of a positive result yet, I haven't seen any information of a direct comparison of this test and the [nasal] swab test so I would trust a swab test more.'*<br><br>*'PCR tests are the most reliable – although the saliva samples are obviously part of a trial so a bit of an unknown, but still feel confident it will pick up most positives, and probably more accurately than a lateral flow.'*

Compliance with public health guidelines

Respondents were asked whether they had changed their approach to the public health guidelines that were in place at the time of the study (i.e., social distancing and face coverings) at work or study since they joined TestEd. The majority (93%) indicated that they had not changed their approach. Only 5% reported that they had, and 2% did not know. Responses to this question were similar between the pilot and main survey.

Among the small number of participants who indicated that they had changed their behaviour, some participants reported feeling more relaxed with regard to guidelines. In some cases this made them less adherent and in other cases it made them more confident to mix with others within the guidelines. Others who reported changing their behaviour following participation in TestEd explained that the testing programme had resulted in them following guidelines more stringently, for example with reference to wearing face masks:

For peer review only - http://bmjopen.bmj.com/site/about/guidelines.xhtml

'I was careful before as I wore FFP2 masks when in enclosed spaces. I am more reluctant to visit crowded public spaces as I worry that I could then test positive.'

'Am less worried about interacting with friends and family given negative tests, so I see more people if I've been regularly testing.'

'I confess I am a little less strict than before in following the guidelines. I sometimes forgot I do not wear a mask. This may be due to the fact that I feel less worried about catching it.'

In interviews, all participants who had tested positive reported having booked a confirmatory test through the NHS, to have informed their workplace, and to have fully complied with self-isolation guidelines. However, some also indicated challenges, including the effects on others of their decision to self-isolate, financial consequences, impacts on personal wellbeing, and a reliance on their own social networks for emotional support and provisions during the isolation period. Some interviewees highlighted issues such as taking out the rubbish, accessing meals, and negotiating spaces with other members of a household who had not tested positive:

'I was kind of really bored in my room, because in my flat there's one other person so I tried my best not to go in the kitchen or the living room. The only true place I can go is my bedroom and the bathroom. So it was quite difficult because I felt like I was also inconveniencing her; if I wanted water or food or something she had to bring it to my door. Although I am more than capable of making myself a cup of tea, I didn't want to go into the kitchen and accidentally contaminate things.'

'I didn't want to trouble other people to carry all my groceries for me. And it's not enough to stack up to the minimum delivery. So I just ended up trying to make do, asking people if they could just buy one or two things for me and stuff. So that's one very big inconvenience.'

Discussion

This study adds to evidence from previous research that routine asymptomatic testing for SARS-CoV-2 can be introduced on university campuses in a way that is accessible and acceptable to staff and students. Although TestEd was used by a minority of students and university employees during the study period, the programme was introduced at a time when working from home guidance was in place and footfall on campus was low.[2] For those that regularly participated, enthusiasm for the availability of free asymptomatic testing was maintained over time.

Reasons for taking part included participants wanting to know their own Covid status and avoiding passing the virus onto others, which confirms findings on attitudes to Covid-19 testing from studies in multiple countries.[17] Despite TestEd being a workplace programme, concern for others was not necessarily limited to colleagues and instead also related to protecting vulnerable friends and relatives off-campus. Early in the pandemic it was suggested that highly interconnected social networks inside and outside university make it a high-risk environment.[18] Our findings suggest that university staff and students are aware of these risks and are willing to take active measures to reduce them.

Previous research has shown that concerns about physical discomfort and the capacity to perform nasopharyngeal swab-based sample collection are barriers to participation in testing.[19,20] TestEd involved a novel saliva-based sampling method for PCR testing, avoiding nasal pharyngeal swabs. Participants reported that this was a more comfortable

For peer review only - http://bmjopen.bmj.com/site/about/guidelines.xhtml

form of testing. However, there were some concerns about producing enough saliva and around privacy while spitting into a cup at testing sites. Other university-based studies have found similar concerns among participants about their ability to perform saliva-based testing.[21,22] One study that compared saliva- and swab-based testing methods found no consensus among participants on the preferred method.[6] While saliva-based testing has some advantages over swab testing in terms of physical comfort, our findings show that it can also introduce new challenges and concerns for participants.

The convenience of testing was something participants valued, confirming findings from other studies that have found convenience to be a key facilitator for Covid-19 testing uptake.[8,9,22,23] Aspects of the TestEd programme that were found to be convenient included the sample collection method and the quantity and accessibility of sample collection points across campus. However, in some instances negative experiences of IT systems used to sign up, submit samples and access results negatively affected perceptions of convenience. Having to wait for results (compared with the quick turnaround time for LFDs) was also a disadvantage. Our findings show that the perceived convenience of a particular testing method varied in relation to the context for and purpose of testing. Because TestEd was in place when other forms of free testing were available (via the NHS for those with symptoms and LFDs for asymptomatic testing in wider society) it is unsurprising that participants combined different kinds of tests according to which was deemed most convenient at a particular moment.

Participation in TestEd was reported as being reassuring for participants, consistent with previous research on Covid-19 testing in education settings[6,8,22] and workplaces.[24,25] Our results found that this reassurance was, however, mediated by perceptions about levels of participation in the testing programme by others. Participants were sensitive to the question of whether they were part of a larger testing community, in part because they understood that the effectiveness of the programme as a public health screening tool depended on others also taking part.

Previous studies have found that concerns about the accuracy of LFDs can be a barrier to participation in testing.[7,9,11] We found that survey and interview participants were aware of differences in the sensitivity of PCR compared to LFDs, and perceived PCR to be a more accurate testing method. Saliva-based self-testing was also perceived to be more accurate than self-testing with a nasopharyngeal swab. Participating in a programme developed by university scientists provided some reassurance that testing results were likely to be accurate.

There was limited evidence that testing resulted in changes in behaviour among those who participated, for example leading to increased confidence to socialise, both within and outside existing guidelines. Similar findings have been reported for other university-based studies[6,8] and for workplace studies of antibody testing.[25] In line with findings from previous studies[6,26,27] participants experienced daily challenges during self-isolation, such as when isolating from other members of the household,[28] but this did not affect self-reported compliance with guidelines.

We collected information about participant characteristics but did not identify any significant differences in survey responses between groups, although our samples may have been too small to examine relevant characteristics (such as disabilities or ethnicity) in detail.

For peer review only - http://bmjopen.bmj.com/site/about/guidelines.xhtml

A limitation of this study is that it did not include the views of staff or students who did not participate in TestEd, despite visiting campus during the study period. There were varying public health regulations and guidance in place over the period of the research[2] and limited available information about footfall on campus, given the size and complexity of a large university. It is therefore difficult to assess how many staff and students would have used the programme if everyone eligible to do so had signed up. In order to begin to understand reasons for non-participation in TestEd we have recently engaged with the University of Edinburgh student panel, a group of 250 students designed to be representative of the student population. While almost all of those who responded to our brief online questionnaire to the panel (n=76, 30% response rate) had heard of TestEd, most chose not to participate because they didn't get round to registering, preferred not to know if they had Covid, or used LFDs instead.

Engagement with TestEd is voluntary, meaning that the participant population may differ from the student and staff population as a whole. We could not explore further differences between the TestEd population and the university population due to a lack of available data. Survey participants may also differ from the wider population of TestEd programme participants. The survey response rates were reasonably high (72% for the pilot survey and 66% for the main survey). However, when comparing the characteristics of the survey respondents to all TestEd participants we noted some differences, for example that there were more women amongst the survey respondents. There may therefore be biases in the survey responses due to the nature of the survey sample.

Conclusion

Despite alternative testing options being available in the community at the time of the research, our results indicate that an asymptomatic SARS-CoV-2 testing programme designed specifically for university staff and students was acceptable and was positively received by those who took part. Provision of multiple testing sites across campus and the ease of saliva sampling compared to swabs were facilitators to participation, as were perceptions about the accuracy of results from PCR testing compared with LFDs. Potential barriers to participation included concerns about privacy when providing a sample; difficulty in accessing and using IT systems; time to receiving results; and concerns about the extent to which the testing would reduce the risk of outbreaks on campus in the case of low levels of participation in the programme. Perceptions of convenience shaped facilitators and barriers to participation at every stage of the testing process. Availability of testing did not appear to undermine protective behaviours among participants to follow Covid-19 guidelines. These findings suggest that saliva-based PCR asymptomatic testing offers an acceptable and alternative and/or complement to LFD asymptomatic testing on university campuses. Future studies should explore reasons for non-participation in testing programmes in similar workplace or educational settings.

Summary

Section 1: What is already known on this topic

- Pilot studies have found high levels of acceptability of asymptomatic Covid-19 testing programmes among UK university staff and students but have not established whether or not acceptability can be sustained over longer time-periods.
- User concerns about the accuracy and discomfort of self-testing with LFDs are known barriers to participation in Covid-19 testing programmes.
- There is limited evidence as to whether regular asymptomatic testing encourages behavioural change in relation to Covid-19 protective behaviours and compliance with guidelines.

Section 2: What this study adds

- Regular asymptomatic saliva-based PCR testing is acceptable to university staff and students.
- The acceptability of voluntary asymptomatic testing programmes depends on participant perceptions of test accuracy and overall participation levels, and on experiences of testing convenience and comfort.
- We did not find evidence in our survey or interview data to suggest that participation in asymptomatic testing leads to non-compliance with public health guidelines for protective behaviours.

Data availability statement

Survey data relevant to the study is accessible from the University of Edinburgh DataShare, DOI: [to be made available prior to publication]

Ethics Statements

Patient consent for publication
Not applicable.

Ethics approval
This study involves human participants and approval was obtained from the University of Edinburgh's Medical School Research Ethics Committee (Ref: 20-EMREC-023_SA03). Participants gave informed consent to participate in the study before taking part.

Acknowledgements

We would like to thank Professors Neil Turok and Nick Gilbert (TestEd co-investigators) for their support for the survey and qualitative research elements. In addition, Professor Jonathan Seckl, Senior Vice Principal at the University of Edinburgh, who facilitated University pump priming funding for setting up the programme and assisted with liaising with key University staff and student networks. Kathryn Carruthers was TestEd project manager when the study took place and her assistance was invaluable. Finally, we thank the University of Edinburgh staff and students who participated in the research.

Contributor Statement

For peer review only - http://bmjopen.bmj.com/site/about/guidelines.xhtml

LB, AS, HS, RC and TA contributed to the design of this study. LB and TA initiated the project. LS and TS project managed the research. Statistical analysis was done by YM and MB supported by RC and HS. Qualitative analysis was conducted by IS and SC supported by AS. All authors contributed to the manuscript and read and approved the final manuscript. They have all read and approved the final manuscript.

Competing Interests

LB is Chief Social Policy Adviser to the Scottish government (part-time secondment) and chairs the Universities and Colleges Advisory Group, a subgroup of the Chief Medical Officer of Scotland's Advisory Group on Covid-19.

HS has received payment from the Scottish Parliament for advising the COVID and COVID recovery committees.

AS receives funding from the European Research Council (Grant Number 715450) for Investigating the Design and Use of Diagnostic Devices in Global Health, and holds positions on the Royal Anthropological Institute Medical Committee (unpaid), and the Wellcome Trust Career Development Committee (paid).

TA receives internal support from the University of Edinburgh. As the founder and director of BioCaptiva (a liquid biopsy company unrelated to the present study), he receives consulting fees. Additionally, he has received travel expenses for the Biomarkers UK Congress, Oxford Global, November 2021, and Liquid Biopsies, Global Engage conference, December 2021. TA is the Regional Champion for Scotland for the Academy of Medical Sciences, and sits on the Genomics England Scientific Advisory Committee, European Research Council advanced grant panel for genetics. He is also a Fellow of the Royal College of Physicians of London and Edinburgh, and trustee and director of the PHG Foundation.

Funding statement

This work was supported by UKRI Research Grant MR/W006243/1 and the University of Edinburgh. Additional support for manuscript preparation was from the DiaDev project funded by the European Research Council (ERC) European Union's Horizon 2020 Research and Innovation Programme, grant agreement 71540.

Data sharing statement

Survey data relevant to the study is accessible from the University of Edinburgh's open access DataShare, DOI: [to be made available prior to publication]

References

1 SAGE. SAGE Paper on Further and Higher Education. *Sage* 2020;**46**. https://assets.publishing.service.gov.uk/government/uploads/system/uploads/attachment_data/file/903321/S0599_SAGE_Paper_on_Further_and_Higher_Education_ORIGINAL.pdf (accessed 26 Apr 2022).

For peer review only - http://bmjopen.bmj.com/site/about/guidelines.xhtml

2 Scottish Government. Coronavirus (COVID-19): universities, colleges and community learning and development providers. 2022. https://www.gov.scot/publications/coronavirus-covid-19-universities-colleges-and-community-learning-and-development-providers/ (accessed 26 Apr 2022).

3 Yamey G, Walensky RP. Covid-19: Re-opening universities is high risk. *BMJ* 2020;**370**.https://doi.org/10.1136/BMJ.M3365.

4 Independent SAGE. Independent SAGE Statement on Universities in the Context of SARS-CoV-2. The Independent SAGE Report 11. Published Online First: 2020. www.independentSAGE.org (accessed 30 Mar 2022).

5 SAGE. Multidisciplinary Task and Finish Group on Mass Testing: Consensus Statement for SAGE. *Sage* 2020;**53**. https://assets.publishing.service.gov.uk/government/uploads/system/uploads/attachment_data/file/914931/s0712-tfms-consensus-statement-sage.pdf.

6 Blake H, Corner J, Cirelli C, *et al.* Perceptions and Experiences of the University of Nottingham Pilot SARS-CoV-2 Asymptomatic Testing Service: A Mixed-Methods Study. *Int J Environ Res Public Health* 2021;**18**:1–26. https://doi.org/10.3390/ijerph18010188.

7 Gillam TB, Cole J, Gharbi K, *et al.* Norwich COVID-19 testing initiative pilot: evaluating the feasibility of asymptomatic testing on a university campus. *J Public Health (Oxf)* 2021;**43**:82–8. https://doi.org/10.1093/pubmed/fdaa194.

8 Wanat M, Logan M, Hirst JA, *et al.* Perceptions on undertaking regular asymptomatic self-testing for COVID-19 using lateral flow tests: A qualitative study of university students and staff. *BMJ Open* 2021;**11**. https://doi.org/10.1136/bmjopen-2021-053850.

9 Hirst J, Logan M, Fanshawe TR, *et al.* Feasibility and Acceptability of Community COVID-19 Testing Strategies (FACTS) in a University Setting. *SSRN Electronic Journal* 2021;1–8. https://doi.org/10.2139/ssrn.3840101.

10 Department for Education. Christmas guidance set out for university students. 2020. https://www.gov.uk/government/news/christmasguidance-set-out-for-university-students.

11 French CE, Denford S, Brooks-Pollock E, *et al.* Low uptake of COVID-19 lateral flow testing among university students: a mixed methods evaluation. *Public Health* 2022;**204**:54–62. https://doi.org/10.1016/j.puhe.2022.01.002.

12 Jones LF, Batteux E, Bonfield S, *et al.* Durham University students' experiences of asymptomatic COVID-19 testing: a qualitative study. *BMJ Open* 2021;**11**:e055644. https://doi.org/10.1136/bmjopen-2021-055644.

For peer review only

For peer review only - http://bmjopen.bmj.com/site/about/guidelines.xhtml

13 Raffle AE, Pollock AM, Harding-Edgar L. Covid-19 mass testing programmes Should be modelled on successful screening programmes. *BMJ* 2020;**370**:10–1. https://doi.org/10.1136/bmj.m3262.

14 Wise J. Covid-19: Lateral flow tests miss over half of cases, Liverpool pilot data show. *BMJ* Published Online First: 2020. https://doi.org/10.1136/bmj.m4744.

15 Mahase E. Covid-19: Universities roll out pooled testing of students in bid to keep campuses open. *BMJ* 2020;**370**:m3789. https://doi.org/10.1136/bmj.m3789.

16 Reijns MAM, Thompson L, Acosta JC, *et al.* A sensitive and affordable multiplex RT-qPCR assay for SARS-CoV-2 detection. *PLoS Biol* 2020;**18**:1–20. https://doi.org/10.1371/journal.pbio.3001030.

17 Bevan I, Stage Baxter M, Stagg HR, *et al.* Knowledge, attitudes, and behavior related to covid-19 testing: A rapid scoping review. *Diagnostics (Basel)*. 2021;**11**. https://doi.org/10.3390/diagnostics11091685.

18 Nixon E, Trickey A, Christensen H, *et al.* Contacts and behaviours of university students during the COVID-19 pandemic at the start of the 2020/2021 academic year. *Sci Rep* 2021;**11**:1–13. https://doi.org/10.1038/s41598-021-91156-9.

19 Zimba R, Kulkarni S, Berry A, *et al.* SARS-CoV-2 testing service preferences of adults in the United States: Discrete choice experiment. *JMIR Public Health Surveill* 2020;**6**:1–7. https://doi.org/10.2196/25546.

20 Kernberg A, Kelly J, Nazeer S, *et al.* Universal Severe Acute Respiratory Syndrome Coronavirus 2 (SARS-COV-2) Testing Uptake in the Labor and Delivery Unit: Implications for Health Equity. *Obstet Gynecol* 2020;**136**:1103–8. https://doi.org/10.1097/AOG.0000000000004127.

21 Ehrenberg AJ, Moehle EA, Brook CE, *et al.* Launching a saliva-based SARS-CoV-2 surveillance testing program on a university campus. *PLoS ONE* 2021;**16**:1–20. https://doi.org/10.1371/journal.pone.0251296.

22 Watson D, Baralle NL, Alagil J, *et al.* How do we engage people in testing for COVID-19? A rapid qualitative evaluation of a testing programme in schools, GP surgeries and a university. *BMC Public Health* 2022;**22**:1–11. https://doi.org/10.1186/s12889-022-12657-4.

23 Blake H, Knight H, Jia R, *et al.* Students' views towards Sars-Cov-2 mass asymptomatic testing, social distancing and self-isolation in a university setting during the COVID-19 pandemic: A qualitative study. *Int J Enviro Res Public Health* 2021;**18**. https://doi.org/10.3390/ijerph18084182.

24 de Camargo C. 'It's tough shit, basically, that you're all gonna get it': UK virus testing and police officer anxieties of contracting COVID-19.' *Policing Soc* 2021;**0**:1–17. https://doi.org/10.1080/10439463.2021.1883609.

For peer review only - http://bmjopen.bmj.com/site/about/guidelines.xhtml

25 Ljubić T, Banovac A, Buljan I, *et al.* Effect of SARS-CoV-2 antibody screening on participants' attitudes and behaviour: a study of industry workers in Split, Croatia. *Public Health* 2021;**191**:11–6. https://doi.org/10.1016/j.puhe.2020.12.001.

26 Missel M, Bernild C, Dagyaran I, *et al.* A stoic and altruistic orientation towards their work: a qualitative study of healthcare professionals' experiences of awaiting a COVID-19 test result. *BMC Health Serv Res* 2020;**20**:1–9. https://doi.org/10.1186/s12913-020-05904-0.

27 Allen WE, Altae-Tran H, Briggs J, *et al.* Population-scale longitudinal mapping of COVID-19 symptoms, behaviour and testing. *Nat Hum Behav* 2020;**4**:972–82. https://doi.org/10.1038/s41562-020-00944-2.

28 Wallis G, Siracusa F, Blank M, *et al.* Experience of a novel community testing programme for COVID-19 in London: Lessons learnt. *Clin Med (Lond)* 2020;**20**:E165–9. https://doi.org/10.7861/CLINMED.2020-0436.

For peer review only

For peer review only - http://bmjopen.bmj.com/site/about/guidelines.xhtml

Supplementary File 1: TestEd Participant Pilot Survey

Notes for entry online:

BLUE = The question/variable name.
RED = Skip, display, or loop logic.
GREEN = New Block
PURPLE = Forced response
- -

INTRO BLOCK

INTRODUCTION PAGE

I This survey is about student and staff experiences of asymptomatic COVID-19 testing at the University, delivered via the TestEd programme. Before agreeing to take part and proceeding to answer the survey questions, we'd like to remind you of what the survey involves and how the responses you provide will be used.

This information can also be found in the Participant Information Sheet for TestEd, which is available at:

https://www.ed.ac.uk/files/atoms/files/participant_information_sheet_v3.0_28_january_2021_clean.pdf

The survey will ask about your experiences of participating in TestEd. Your responses will help improve the programme as it is rolled out. Participation is voluntary and the survey should take about 10-15 minutes to complete.

Your anonymous survey data will be imported into quantitative data analysis software for analysis by the research team. The survey data will be retained on our server for a minimum of 5 years after the end of the study.

The anonymised results of this survey may be quoted in reports and academic publications produced by the study team. Your name will never be used in any of these reports or publications and they will not include any personal identifiable information about you.

At the end of this survey we ask if you would be willing to be re-contacted to participate in a follow-up interview with a researcher if you receive a positive result for Covid-19 from the TestEd programme. This interview is voluntary.

TestEd Participant Pilot Survey. 15th of April 2021

For peer review only

CONSENT

In agreeing to participate in this survey, you confirm the following:

1. I confirm that I have read and understood the Participant Information Sheet for TestEd.

2. I understand that my participation is voluntary and that I can ask to withdraw at any time without giving a reason and without my legal rights being affected.

3. I understand that once the survey form is submitted it will not be possible to withdraw from the survey. This is because no identifiable information will be stored with the survey data.

4. I confirm that I am happy for my survey responses to be linked to anonymised demographic data (age, gender, ethnicity, staff or student, whether living in University owned accommodation or elsewhere) provided by me when I registered to participate in the TestEd programme

5. I confirm that I am happy for anonymised data from this survey to be published for research purposes.

6. I understand that my anonymised data will be stored for a minimum of 5 years and may be used in future ethically approved research.

Should you have any further questions about this survey or any element of TestEd please contact us via TestEd@ed.ac.uk

By ticking this box, I agree to the above consent points and to take part in the above study

TestEd Participant Pilot Survey. 15th of April 2021

For peer review only

Q8

How much time did you take out of your day to provide a TestEd sample (not including travel time, i.e. collecting a sample pack, providing the sample and dropping off your sample)?

a. 1-2 minutes
b. 2-5 minutes
c. 5-10 minutes
d. More than 10 minutes

- -

TEST TRUST BLOCK

Q15

Do you believe that the result you received from Test Ed was accurate?

a. Yes
b. No
c. Unsure

Q16

Why did you believe the result was accurate/inaccurate?

[free text box]

Q17

Does the availability of the TestEd programme make you feel reassured about working/studying on campus?

a. Yes
b. No
c. Unsure [skip to Q19]

Q18

Could you explain a bit more about why you felt reassured or not?

[free text box]

- -

POST-TEST ATTITUDES AND BEHAVIOUR BLOCK

For peer review only

Q20

Have you changed your approach to public health guidelines (i.e. social distancing, face coverings, hygiene)

since you joined TestEd?

a. Yes
b. No [skip to Q24]
c. I don't know [skip to Q24]

Q21

Can you tell us about how your approach to public health guidelines has changed since your joined TestEd?

[free text box]

Q27

Overall, how would you rate your experience of the TestEd programme?

a. Excellent
b. Good
c. Fair
d. Poor
e. Very poor

Q28

Is there anything else you would like to tell us about your testing experience?
[free text box]

PAGE BREAK

For peer review only

Supplementary File 2: TestEd Participant Main Survey

Notes for entry online:

BLUE = The question/variable name.
RED = Skip, display, or loop logic.
GREEN = New Block
PURPLE = Forced response

- -

INTRO BLOCK

INTRODUCTION PAGE

Thank you very much for participating in TestEd!

Any feedback you are able to provide will help us to improve the system for you and for other users.

This survey is about student and staff experiences of the University of Edinburgh's COVID-19 testing project for people that are not showing any symptoms, TestEd. Before asking you to agree to take part, we'd like to remind you of what the survey involves and how the responses you provide will be used. Participation in this survey is entirely voluntary and the survey will take you about 10-15 minutes to complete.

This information can also be found in the Participant Information Sheet for TestEd, which is available at:

https://www.ed.ac.uk/files/atoms/files/participant_information_sheet_v5.0_01_september_2021.pdf

What will happen to my data?

The demographic data (age, gender, disability status, ethnicity, whether you are a student / staff, whether you live in university owned accommodation, and your department), that you provided when you joined TestEd and the dates when you provided a TestEd saliva sample will be linked to your survey responses using your TestEd barcode. Your anonymous survey data will then be imported into quantitative data analysis software for analysis by the research team. The survey data will be retained on our Sharepoint server for a minimum of 5 years after the end of the study.

For peer review only - http://bmjopen.bmj.com/site/about/guidelines.xhtml

The anonymised results of this survey may be quoted in reports and academic publications produced by the study team, which will help others to learn from TestEd's experience.Your name will never be used in any of these reports or publications and they will not include any personal identifiable information about you.

At the end of this survey we ask if you would be willing to be re-contacted to participate in a follow-up interview with a researcher from the TestEd programme. This interview is also entirely voluntary. If you tell us that you are interested in taking part in an interview, your demographic data will be shared with the TestEd interviewer.

PAGE BREAK

CONSENT

Thank you to those who completed a TestEd survey in Apr-21. We really want to hear from you again. This new survey contains some of the same questions that we asked you the last time. We really appreciate you taking the time to answer these again.

In agreeing to participate in this survey, you confirm the following:

1. I confirm that I have read and understood the Participant Information Sheet for TestEd V5.0 01 September 2021.

2. I understand that my participation is voluntary and that I can ask to withdraw at any time without giving a reason and without my legal rights being affected.

3. I understand that my rights to access, change or move my information are limited once the survey form is submitted and that you will keep the information provided even if I decide to withdraw from the survey or the TestEd study at a later date.

4. I confirm that I am happy for my survey responses to be linked to my anonymised demographic data (age, gender, disability status, ethnicity, whether you are a student / staff, whether you live in university owned accommodation, and your department) and dates when I provided TestEd saliva samples collected as part of the TestEd programme.

5. I confirm that I am happy for anonymised data from this survey to be published for research purposes.

6. I understand that my anonymised data will be stored for a minimum of 5 years and may be used in future ethically approved research.

7. I agree to take part in this TestEd survey.

Should you have any further questions about this survey or any element of TestEd please

For peer review only - http://bmjopen.bmj.com/site/about/guidelines.xhtml

contact us via TestEd@ed.ac.uk .

By ticking this box, I agree to the above consent points.

Q12

Please rank from most to least important what you believe are the benefits of taking part in TestEd (you may drag and drop from most to least important)

_______ To know own Covid-19 status in the absence of symptoms;

_______ To prevent from passing on infection to other colleagues/students on campus if I am positive;

_______ To prevent from passing on infection to family and friends outside the University if I am positive;

_______ To contribute to scientific research on Covid-19;

_______ Because other people are using TestEd, and I feel I should too.

PAGE BREAK

Q13

Are there any other benefit(s) to taking part in TestEd (optional)

[free text box]

PAGE BREAK

Q19

How much time did you take out of your day to provide a TestEd sample (i.e providing the sample and registering it on the system)?

a. 1-2 minutes
b. 2-5 minutes
c. 5-10 minutes
d. More than 10 minutes

PAGE BREAK

Q20

How convenient do you find it to provide a TestEd sample as part of your work/study schedule?

a. Very convenient [skip to Q22]
b. Convenient [skip to Q22]
c. Neutral [skip to Q23]
d. Inconvenient
e. Very inconvenient

PAGE BREAK

For peer review only

Q21
You have said that you find it inconvenient to provide a TestEd sample as part of your work/study schedule. Why is this? (optional)
[free text box]

PAGE BREAK

Q22
You have said that you find it convenient to provide a TestEd sample as part of your work/study schedule. Why is this? (optional)
[free text box]

PAGE BREAK

Q25
Prior to joining TestEd, how concerned were you about catching Covid-19 on campus?

a. Very concerned
b. Moderately concerned
c. Somewhat concerned
d. Slightly concerned
e. Not at all concerned [skip to Q27]

Q26
You have said that you had concerns about catching Covid-19 on campus prior to joining TestEd. Please briefly describe what were your main concerns (optional)
[free text box]

PAGE BREAK

Q27
You have said that you did not have concerns about catching Covid-19 on campus prior to joining TestEd why is this? (optional)
[free text box]

PAGE BREAK

Q28
Do you believe that the result(s) you received from Test Ed so far were accurate?

a. Yes
b. No
c. Unsure

For peer review only

For peer review only - http://bmjopen.bmj.com/site/about/guidelines.xhtml

PAGE BREAK

Q29

Why did you believe the result(s) were accurate/inaccurate?

[free text box]

PAGE BREAK

Q31

Does the availability of the TestEd programme make you feel reassured about working/studying on campus?

a. Yes
b. No
c. Unsure

- -

TEST TRUST BLOCK

Q32

Could you explain a bit more about why you felt reassured or not?

[free text box]

- -

POST-TEST ATTITUDES AND BEHAVIOUR BLOCK

Q33

Have you changed your approach to public health guidelines (i.e. social distancing, face coverings, hygiene) since you joined TestEd?

a. Yes [display 34 to Q35]
b. No [skip to Q35]
c. I don't know [skip to Q35]

Q34

Can you tell us about how your approach to public health guidelines (i.e face coverings, hygiene) has changed since your joined TestEd?

For peer review only - http://bmjopen.bmj.com/site/about/guidelines.xhtml

[free text box]

PAGE BREAK

Q35

Overall, how would you rate your experience of the TestEd programme?

a. Excellent

b. Good

c. Fair

d. Poor

e. Very poor

For peer review only

For peer review only - http://bmjopen.bmj.com/site/about/guidelines.xhtml

Supplementary File 3 - Test-Ed COVID-19 Testing Project Interview Topic Guide

Round 2 Version 1.0 01 September 2021

1. Context – experience of the Covid-19 pandemic

Could you tell me a bit about your current role/work/study at the University of Edinburgh just for context? How long have you worked/studied here?

How has the Covid-19 pandemic affected your work/study at the University of Edinburgh since the beginning of 2020?

Prompt: change in working/study location/ change in routine/ contact with peers/colleagues

Prior to joining TestEd did you have any concerns about Covid-19 on campus? Why/What were these?

What kind of impact has the Covid-19 pandemic had on you personally over the past 20 months? [Follow up on leads from the answer to this question e.g. around travel to see family/friends; concerns about personal health/health of family and friends; personal experience of Covid infection prior to joining TestEd etc.]

And how would you describe the impact the pandemic is having on your life now? Do you feel that your life is back to normal and if not, what is different? Do you worry about Covid-19 in your everyday life right now? If so, in what way?

Before starting the TestEd programme, had you had any reason to get tested for Covid-19? Can you tell us about that experience?

Prompts: Why sought testing, experience of accessing a test, physical experience of undergoing testing, response to results

Before starting the TestEd programme, had you had any reason to isolate (prompt contacted by Test and Protect/ pinged by app)? Can you tell us about that experience?

Prompts: What did they find most difficult about isolating? Where they on their own? How did they get food? Was there any reason they had to leave the house?

And in terms of your personal circumstances, do you live with others at the moment? Prompt: type of housing (i.e. for students if in halls or elsewhere, for all - living with children, older adults etc.); Do you have any caring responsibilities for others (either inside or outside your household)?

For peer review only

Interview Consent Script and Topic Guide Round 2 V1.0 01 September 2021

2. Experience of TestEd

I would now like to ask a few more general questions about your experience of the TestEd programme.

Could you tell me how you heard about the programme (Test-Ed)? When was this?

Could you tell me why you decided to join the testing programme? What did you see as the main benefits of the TestEd programme?

Did you have any hesitation in joining the programme - Why?

Prompt: Any concerns about privacy of data?

Did you know anyone else who was already a part of the TestEd programme when you joined? Did you discuss your decision to join the programme with them (what did you discuss)?

Can you tell us about the process you went through to be tested for the first time?

Prompt: Where did they get tested? Did they understand what was required of them? What was their physical experience of the sample collection process? Did they have concerns about privacy related to the sample booth? How did this experience compare to any other Covid-19 testing experiences you have had (lateral flow/NHS PCR?). How quickly got results? Method for receiving results straightforward?

How have you made use of the TestEd programme since that first test?

Prompt: How often do you provide a sample? How do you fit the testing into your work/study routine? Are there any reasons why you have missed a test?

Prompt: How has your use of TestEd changed over time?

Prompt: Have you ever given a TestEd sample when you had symptoms linked to Covid-19 e.g. a cough or fever?

One concern that is often expressed about asymptomatic testing is that people might not follow public health guidelines (i.e. wearing face coverings, hand and respiratory hygiene etc) if they are being regularly tested. What is your view on that, and did you feel like that at all while you've been taking part in the TestEd programme?

Prompt: If they didn't feel like that, why not. If they did feel like that, how did it affect their behaviour?

Have you been vaccinated?

Could you tell me a bit more about when you had your first and second dose and timing of those?

Has being vaccinated changed how you feel about working/studying on campus? In what ways?

Do you think you have changed your everyday behaviour or routines in any way since you were vaccinated?

Prompt: Do you feel more protected since you have been vaccinated?

Has participation in the TestEd programme made you feel safer on campus? (note for interviewer: for those who report currently working/studying on campus)

Prompt: if not, why? Did you have any specific concerns about safety on campus prior to joining TestEd?

3. Story of Positive Test Result [for participants who have tested positive]

Can you tell me about your experience of testing positive for Covid-19 with TestEd? It would be really helpful if you could take us through your experience chronologically, starting before you were tested.

Prompts: Try to find out a clear timeline of events

Did you notice any changes in how you were feeling before you got tested?

What day of the week did you get tested and what else were you doing that day? What did you do after you gave your sample?

Where were you when you received the test result? Were you with anyone else? How did you feel when you got the result?

Did you have any worries or concerns **for yourself** following your positive test result? If so, could you tell us about them?

Did you have any worries or concerns **for others** following your positive test result? If so, could you tell us about them?

Did you think the test result was accurate at this point? Why? Why not?

Do you think your vaccination status affected your response to the test result?

What was your first response to the message? What did you do next? Did you tell anyone else your result at this point?

What contact did you have with the TestEd team? Did they give you advice on what to do next? What was that advice? Did you have any trouble following it?

Did you have food in the house? Did you have any other reason you needed to leave the house that day?

Did you need to rearrange plans because of the positive test result?

When did you book your NHS test? What did you do while you were waiting for your test? Can you take us through your experience of the NHS test? e.g. where did you go to get tested? How did you get there? What was your physical experience of the test? How did you feel while you were getting the test done? What did you do while waiting for the result? When did you get the result? How did that make you feel?

Did you have lateral flow tests in the house at the point that you received the test result? When and why had you ordered these (if not already addressed in previous questions). Did you or anyone else in your household use a lateral flow test at any point after you received the TestEd result? Can you tell us about your experience of this? Did you think the result was accurate (why/why not)?

Can you tell us about your experience of self-isolation?

Prompt: Did you feel clear about the self-isolation guidelines at the point that you tested positive? Were there any guidelines you found difficult to follow? How did you organise food (can also follow up on whether they used the food voucher they would have been given)? What was your daily routine during self-isolation? Did you have any reason you needed to leave the house during that period? If in shared house how did you manage your contact with other household members? What did you find most challenging about this period? Is there any other support that might have helped you self-isolate? Who from?

Did you experience any symptoms after your positive test? Prompt: talk through any symptoms or not after the TestEd positive result and

while waiting for/after the confirmatory test

To what extent did you trust the test result from TestEd? Why? Why not?

4. Closing questions

For peer review only

How could the TestEd testing experience have been improved?

Do you intend to continue participating in the TestEd programme? How do you think you will use it in the future?

Has your vaccination status affected your interest in participating in the programme at all?

Would you encourage others to participate in the TestEd programme. Prompt: if yes, why, if no, why not

Is there anything else you'd like to tell us about your experience of participating in the programme?

Would you be willing to be contacted for a short follow-up interview in the future?

Thank you for taking time to take part in this interview. [ENDS]

For peer review only

Supplementary File 4 – Survey Results (for both Pilot and Main Surveys) Tables

Pilot survey	Q8 Time taken to take test?				Q15 Belief in test result?			Q17 Does TestEd make you feel reassured?			Q20 Change in approach to public health guidelines?		Q27 Experience of TestEd programme?			
Response options<br>Demographics	1-2 min.	2-5 min.	5-10 min	>10 min	Yes	No	Unsure	Yes	No	Unsure	Yes	No	Excellent	Good	Fair	Poor
**Total**	223(42.7)	235(45.0)	57(10.9)	7(1.3)	472(90.4)	2(0.4)	48(9.2)	470(90.2)	17(3.3)	34(6.5)	16(3.1)	497(96.9)	408(78.2)	108)20.7)	6(1.2)	0(0.0)
**Gender** *n*(%)																
Female	151(45.6)	140(42.3)	37(11.2)	3(0.9)	300(90.6)	1(0.3)	30(9.1)	300(90.9)	8(2.4)	22(6.7)	9(2.8)	317(97.2)	252(76.1)	75(22.7)	4(1.2)	0(0.0)
Male	71(37.4)	95(50.0)	20(10.5)	4(2.1)	171(90.0)	1(0.5)	18(9.5)	168(89.0)	9(4.7)	12(6.3)	7(3.8)	179(96.2)	155(81.6)	33(17.4)	2(1.1)	0(0.0)
Comparison (p-value, ϕ_c)	FET = 0.128, ϕ_c = 0.103				FET = 0.942, ϕ_c = 0.021			χ^2 = 0.337, ϕ_c = 0.065			χ^2 = 0.502, ϕ_c = 0.030		FET = 0.400, ϕ_c = 0.059			
**Age (years)** *n*(%)																
≤19	0(0.0)	0(0.0)	0(0.0)	0(0.0)	0(0.0)	0(0.0)	0(0.0)	0(0.0)	0(0.0)	0(0.0)	0(0.0)	0(0.0)	0(0.0)	0(0.0)	0(0.0)	0(0.0)
20-29	77(44.8)	74(43.0)	19(11.1)	2(1.2)	148(86.1)	0(0.0)	24(14.0)	160(93.0)	4(2.3)	8(4.7)	7(4.1)	161(95.8)	134(77.9)	37(21.5)	1(0.6)	0(0.0)
30-39	58(42.0)	66(47.8)	14(10.1)	0(0.0)	123(89.1)	1(0.7)	14(10.1)	124(89.9)	4(2.9)	10(7.3)	1(0.7)	134(99.3)	106(76.8)	29(21.0)	3(2.2)	0(0.0)
40-49	34(41.5)	37(45.1)	9(11.0)	2(2.4)	76(92.7)	1(1.2)	5(6.1)	73(89.0)	4(4.9)	5(6.1)	2(2.4)	80(97.6)	62(75.6)	18(22.0)	2(2.4)	0(0.0)
50-59	38(46.9)	35(43.2)	7(8.6)	1(1.2)	77(95.1)	0(0.0)	4(4.9)	70(86.4)	3(3.7)	8(9.9)	5(6.3)	74(93.7)	65(80.3)	16(19.8)	0(0.0)	0(0.0)
≥60	16(32.7)	23(47.0)	8(16.3)	2(4.1)	48(98.0)	0(0.0)	1(2.0)	43(89.6)	2(4.2)	3(6.3)	1(2.0)	48(98.0)	31(83.7	8(16.3)	0(0.0)	0(0.0)
Comparison (p-value, ϕ_c)	χ^2 = 0.672, ϕ_c = 0.077				FET = 0.044, ϕ_c = 0.114			FET = 0.789, ϕ_c = 0.063			FET = 0.159, ϕ_c = 0.110		FET = 0.825, ϕ_c = 0.072			
**Ethnicity** *n*(%)																
British/Irish/Other white	203(42.8)	213(44.9)	51(10.8)	7(1.5)	432(91.1)	2(0.4)	40(8.4)	424(89.6)	16(3.4)	33(7.0)	11(2.4)	456(97.6)	369(77.9)	100(21.1)	5(1.1)	0(0.0)
Asian/Indian/Pakistani/Bangladeshi/ Chinese/Other Asian	12(48.0)	10(40.0)	3(12.0)	0(0.0)	21(84.0)	0(0.0)	4(16.0)	23(92.0)	1(4.0)	1(4.0)	2(8.7)	21(91.3)	19(76.0)	5(20.0)	1(4.0)	0(0.0)
Mixed/Other ethnic/Other black/Caribbean African	8(36.36)	11(50.0)	3(13.6)	0(0.0)	18(82.8)	0(0.0)	4(18.2)	22(100.0)	0(0.0)	0(0.0)	3(13.6)	19(86.4)	19(86.4)	3(13.6)	0(0.0)	0(0.0)
Comparison (p-value, ϕ_c)	FET = 0.950, ϕ_c = 0.038				FET = 0.264, ϕ_c = 0.062			FET = 0.760, ϕ_c = 0.053			χ^2 = 0.010, ϕ_c = 0.149		FET = 0.499, ϕ_c = 0.052			
**Role in the university** *n*(%)																
Staff	171(44.1)	173(44.6)	38(9.8)	6(1.6)	361(93.0)	2(0.5)	25(6.4)	344(88.9)	13(3.4)	30(7.8)	11(2.9)	372(97.1)	310(79.9)	73(18.8)	5(1.3)	0(0.0)
Students	52(38.8)	62(46.3)	19(14.2)	1(0.8)	111(82.8)	0(0.0)	23(17.2)	126(94.0)	4(3.0)	4(3.0)	5(3.9)	125(96.2)	98(73.1)	35(26.1)	1(0.8)	0(0.0)
Comparison (p-value, ϕ_c)	FET = 0.424, ϕ_c = 0.075				FET = 0.001, ϕ_c = 0.165			FET = 0.160, ϕ_c = 0.085			FET = 0.566, ϕ_c = -0.024		FET = 0.160, ϕ_c = 0.081			
**Disability** *n*(%)																
Yes	3(37.5)	4(50.0)	1(12.5)	0(0.0)	8(100.0)	0(0.0)	0(0.0)	8(100.0)	0(0.0)	0(0.0)	1(12.5)	7(87.5)	7(87.5)	1(12.5)	0(0.0)	0(0.0)
No	215(42.7)	226(44.9)	56(11.1)	6(1.2)	453(90.3)	2(0.4)	47(9.3)	453(90.2)	17(3.4)	32(6.4)	14(2.8)	480(97.2)	393(78.1)	104(20.7)	6(1.2)	0(0.0)
Comparison (p-value, ϕ_c)	FET = 1.000, ϕ_c = 0.020				FET = 1.000, ϕ_c = 0.041			FET = 1.000, ϕ_c = 0.041			FET = 0.217, ϕ_c = 0.071		FET = 0.100, ϕ_c = 0.029			

FET = Fisher's exact test, χ^2 = Chi Square test, ϕ_c = Cramer's V

For peer review only

Main survey	Q12 Most important motivations for taking part in TestEd?				
Response options / Demographics	To know Covid-19 status	To prevent infecting other colleagues/students on campus	To prevent infecting friends/family outside campus	To contribute to Covid-19 research	Because other people are using TestEd, so feel I should too
**Total**	753(38.4)	222(11.3)	619(31.5)	358(18.2)	11(0.6)
**Gender** ***n*****(%)**					
Female	442(39.0)	129(11.4)	360(31.8)	196(17.3)	6(0.5)
Male	298(37.5)	87(11.0)	247(31.1)	157(19.8)	5(0.6)
Comparison (p-value, ϕ_c)	FET = 0.719, ϕ_c = 0.033				
**Age (years)** ***n*****(%)**					
≤19	16(39.0)	11(26.8)	9(22.0)	5(12.2)	0(0.0)
20-29	232(40.5)	59(10.3)	181(31.6)	95(16.6)	6(1.1)
30-39	184(40.2)	49(10.7)	157(34.3)	67(14.6)	1(0.2)
40-49	134(35.5)	47(12.5)	124(32.9)	71(18.8)	1(0.3)
50-59	132(35.6)	37(10.0)	117(31.5)	84(22.6)	1(0.3)
≥60	55(38.5)	19(13.3)	31(21.7)	36(25.2)	2(1.4)
Comparison (p-value, ϕ_c)	χ^2 =0.005, ϕ_c = 0.071				
**Ethnicity** ***n*****(%)**					
British/Irish/Other white	678(38.4)	196(11.1)	563(31.8)	324(18.3)	7(0.4)
Asian/Indian/Pakistani/Bangladeshi/ Chinese/Other Asian	46(43.8)	11(10.5)	28(26.7)	17(16.2)	3(2.9)
Mixed/Other ethnic/Other black/Caribbean African	25(32.1)	12(15.4)	24(30.8)	16(20.5)	1(1.3)
Comparison (p-value, ϕ_c)	FET = 0.092, ϕ_c = 0.063				
**Role in the university** ***n*****(%)**					
Staff	538(37.8)	153(10.8)	460(32.4)	264(18.6)	7(0.5)
Students	208(40.0)	67(12.9)	151(29.0)	90(17.3)	4(0.8)
Comparison (p-value, ϕ_c)	FET = 0.361, ϕ_c = 0.046				
**Disability** ***n*****(%)**					
Yes	22(43.1)	5(9.8)	14(27.5)	10(19.6)	0(0.0)
No	710(38.3)	204(11.0)	593(32.0)	334(18.0)	11(0.6)
Comparison (p-value, ϕ_c)	FET = 0.905, ϕ_c = 0.024				
**Staff role** ***n*****(%)**					
Academic	273(37.6)	67(9.2)	243(33.5)	141(19.4)	2(0.3)
Facilities and estates	40(34.2)	17(14.5)	37(31.6)	21(18.0)	2(1.7)
Administration	94(39.8)	26(11.0)	67(28.4)	49(20.8)	0(0.0)
IT services	32(39.5)	11(13.6)	27(33.3)	11(13.6)	0(0.0)
Comparison (p-value, ϕ_c)	χ^2 = 0.231, ϕ_c = 0.066				

FET = Fisher's exact test, χ^2 = Chi Square test, ϕ_c = Cramer's V

For peer review only

Main survey	Q19 Time taken to take test?				Q20 Convenience to provide test?					Q25 Concern about catching Covid prior to joining TestEd?				
Response options / Demographics	1-2 min.	2-5 min.	5-10 min	>10 min	Very convenient	Convenient	Neutral	Inconvenient	Very inconvenient	Not at all concerned	Slightly concerned	Somewhat concerned	Moderately concerned	Very concerned
**Total**	857(41.8)	948(46.2)	208(10.2)	37(1.8)	1389(67.8)	524(25.6)	105(5.1)	31(1.5)	1(0.1)	131(6.4)	345(16.8)	458(22.3)	680(33.2)	436(21.3)
**Gender *n*(%)**														
Female	489(41.1)	561(47.1)	119(10.0)	21(1.8)	818 (68.7)	291(24.5)	60(5.0)	20(1.7)	1(0.1)	57(6.9)	149(18.1)	191(23.2)	270(32.8)	157(19.1)
Male	356(43.2)	369(44.8)	85(10.3)	14(1.7)	558(67.7)	214 (26.0)	43(5.0)	9(1.1)	0(0.0)	71(6.0)	190(16.0)	257(21.6)	402(33.8)	270(22.7)
Comparison (p-value, ϕ_c)	χ^2 = 0.825, ϕ_c = 0.022				FET = 0.732, ϕ_c = 0.035					χ^2 = 0.470, ϕ_c = 0.043				
**Age (years) *n*(%)**														
≤19	23(56.1)	16(39.0)	2(4.9)	0(0.0)	19(46.3)	15(36.6)	7(17.1)	0(0.0)	0(0.0)	4(9.8)	9(22.0)	9(22.0)	14(34.2)	5(12.2)
20-29	269(45.7)	266(45.2)	49(8.3)	5(0.9)	360(61.1)	186(31.6)	31(5.3)	12(2.0)	0(0.0)	30(5.1)	94(16.0)	160(27.2)	202(34.3)	103(17.5)
30-39	191(40.1)	222(46.6)	56(11.8)	7(1.5)	305(64.1)	131(27.5)	30(6.3)	10(2.1)	0(0.0)	27(5.7)	72(15.1)	101(21.2)	165(34.7)	111(23.3)
40-49	152(39.0)	193(49.5)	38(9.7)	7(1.8)	282(72.3)	88(22.6)	16(4.1)	4(1.0)	0(0.0)	340(7.7)	74(19.0)	82(21.0)	110(28.2)	94(24.1)
50-59	166(42.0)	177(44.8)	42(10.6)	10(2.5)	298(75.4)	79(20.0)	14(3.5)	31(0.8)	1(0.3)	28(7.1)	65(16.5)	85(21.5)	132(33.4)	85(21.5)
≥60	56(35.2)	74(46.5)	21(13.2)	8(5.0)	125(78.6)	25(15.7)	7(4.4)	2(1.3)	0(0.0)	12(7.6)	31(19.5)	21(13.2)	57(35.9)	38(23.9)
Comparison (p-value, ϕ_c)	χ^2 = 0.032, ϕ_c = 0.068				χ^2 <0.001, ϕ_c = 0.088					χ^2 = 0.032, ϕ_c = 0.065				
**Ethnicity *n*(%)**														
British/Irish/Other white	774(42.0)	852(46.2)	186(10.1)	33(1.8)	1262(68.4)	462(25.0)	93(5.0)	28(1.5)	1(0.1)	124(6.7)	320(17.3)	407(22.1)	622(33.7)	372(20.2)
Asian/Indian/Pakistani/Bangladeshi/ Chinese/Other Asian	50(45.1)	48(43.2)	12(10.8)	1(0.9)	61(55.0)	39(35.1)	9(8.1)	2(1.8)	0(0.0)	2(1.8)	12(10.8)	31(27.9)	36(32.4)	30(27.0)
Mixed/Other ethnic/Other black/Caribbean African	27(32.9)	44(53.7)	9(11.0)	2(2.4)	62(75.6)	17(20.7)	2(2.4)	1(1.2)	0(0.0)	5(6.1)	13(15.9)	15(18.3)	21(25.6)	28(34.2)
Comparison (p-value, ϕ_c)	FET =0.692, ϕ_c = 0.031				FET =0.093, ϕ_c = 0.055					χ^2 = 0.001, ϕ_c = 0.081				
**Role in the university *n*(%)**														
Staff	599(40.0)	695(46.5)	170(11.4)	31(2.1)	1055(70.6)	348(23.3)	68(4.6)	23(1.5)	1(0.1)	97(6.5)	259(17.3)	310(20.7)	498(33.3)	331(22.1)
Students	242(45.3)	248(46.4)	38(7.1)	6(1.1)	316(59.2)	173(32.4)	37(7.0)	8(1.5)	0(0.0)	31(5.8)	79(14.8)	143(26.8)	179(33.5)	102(19.1)
Comparison (p-value, ϕ_c)	χ^2 = 0.019, ϕ_c = 0.072				FET<0.001, ϕ_c = 0.112					χ^2 = 0.033, ϕ_c = 0.074				
**Disability *n*(%)**														
Yes	19(36.5)	26(50.0)	5(9.6)	2(3.9)	35(67.3)	11(21.2)	5(9.6)	1(2.0)	0(0.0)	0(0.0)	11(21.2)	13(25.0)	14(26.9)	14(26.9)
No	817(42.2)	889(45.9)	196(10.1)	33(1.7)	1318(68.1)	492(25.4)	96(5.0)	28(1.5)	1(0.1)	129(6.7)	327(16.9)	434(22.4)	646(33.4)	399(20.6)
Comparison (p-value, ϕ_c)	FET = 0.409, ϕ_c = 0.037				FET = 0.561, ϕ_c = 0.024					FET = 0.180, ϕ_c = 0.058				
**Staff role *n*(%)**														
Academic	308(40.8)	355(47.0)	77(10.2)	15(2.0)	512(67.8)	194(25.7)	37(4.9)	12(1.6)	0(0.0)	54(7.2)	130(17.2)	171(22.7)	260(34.4)	140(18.5)
Facilities and estates	57(41.1)	65(47.1)	14(10.1)	2(1.5)	112(81.2)	21(15.2)	4(2.9)	0(0.0)	1(0.7)	3(2.2)	21(15.2)	21(15.2)	43(31.2)	50(36.2)
Administration	85(34.3)	119(48.0)	37(14.9)	7(2.8)	184(74.2)	52(21.0)	7(2.8)	5(2.0)	0(0.0)	17(6.9)	51(20.6)	44(17.7)	73(29.4)	63(25.4)
IT services	36(43.9)	35(42.7)	10(12.2)	1(1.2)	56(68.3)	19(23.2)	6(7.3)	1(1.2)	0(0.0)	7(8.5)	13(15.9)	22(26.8)	26(31.7)	14(17.1)
Comparison (p-value, ϕ_c)	χ^2 = 0.586, ϕ_c = 0.046				χ^2 = 0.023, ϕ_c = 0.082					χ^2 = 0.004, ϕ_c = 0.913				

FET = Fisher's exact test, χ^2 = Chi Square test, ϕ_c = Cramer's V

For peer review only

Main survey	Q28 Belief in test result?			Q31 Does TestEd make you feel reassured?			Q33 Change in approach to public health guidelines?			Q35 Experience of TestEd programme?			
Response options / Demographics	Yes	No	Unsure	Yes	No	Unsure	Yes	No	I don't know	Excellent	Good	Fair	Poor
**Total**	1892(92.3)	4(0.2)	154(7.5)	1787(87.2)	99(4.8)	164(8.0)	94(4.6)	1922(93.3)	44(2.2)	1521(74.2)	500(24.4)	28(1.4)	1(0.1)
**Gender *n*(%)**													
Female	1118(92.3)	1(0.1)	71(6.0)	1048(88.1)	46(3.9)	96(3.9)	49(4.1)	1117(93.9)	24(2.0)	889(74.7)	288(24.2)	12(1.0)	1(0.1)
Male	741(89.9)	2(0.2)	81(9.8)	708(85.9)	53(6.4)	63(7.7)	44(5.4)	761(92.5)	18(2.2)	611(74.2)	199(24.2)	14(1.7)	0(0.0)
Comparison (p-value, ϕ_c)	FET = 0.003, ϕ_c = 0.0735			χ^2 = 0.029, ϕ_c = 0.061			χ^2 = 0.244, ϕ_c = 0.038			χ^2 = 0.470, ϕ_c = 0.043			
**Age (years) *n*(%)**													
≤19	39(95.1)	0(0.0)	2(4.9)	36(87.8)	0(0.0)	5(12.2)	2(5.0)	36(90.0)	2(5.0)	22(52.7)	17(41.5)	2(4.9)	0(0.0)
20-29	536(91.0)	2(0.3)	51(8.7)	528(89.6)	22(3.7)	39(6.6)	29(4.9)	539(91.5)	21(3.6)	398(67.6)	179(30.4)	11(1.9)	1(0.2)
30-39	437(91.8)	0(0.0)	39(8.2)	406(85.3)	28(5.9)	42(8.8)	25(5.2)	441(92.7)	10(2.1)	328(68.9)	140(29.4)	8(1.7)	0(0.0)
40-49	362(92.8)	1(0.3)	27(7.0)	340(87.2)	22(5.6)	28(7.2)	15(3.9)	372(95.4)	3(0.8)	308(79.0)	80(20.5)	2(0.5)	0(0.0)
50-59	366(92.7)	1(0.3)	28(7.1)	338(85.6)	19(4.8)	38(9.6)	7(1.8)	383(97.0)	5(1.3)	333(84.3)	61(15.4)	1(0.3)	0(0.0)
≥60	152(95.6)	0(0.0)	7(4.4)	139(87.4)	8(5.0)	12(7.6)	16(10.1)	140(88.1)	3(1.9)	132(83.0)	23(14.5)	4(2.5)	0(0.0)
Comparison (p-value, ϕ_c)	FET = 0.770, ϕ_c = 0.038			χ^2 = 0.326, ϕ_c = 0.054			χ^2 <0.001, ϕ_c = 0.092			χ^2 <0.001, ϕ_c = 0.126			
**Ethnicity *n*(%)**													
British/Irish/Other white	1711(92.7)	3(0.2)	131(7.1)	1604(86.9)	89(4.8)	152(8.2)	80(4.3)	1729(93.8)	35(1.9)	1390(75.3)	429(23.3)	25(1.4)	1(0.1)
Asian/Indian/Pakistani/Bangladeshi/ Chinese/Other Asian	94(84.7)	0(0.0)	17(15.3)	102(92.0)	3(2.7)	6(5.4)	8(7.2)	96(86.5)	7(6.3)	70(63.1)	41(37.0)	0(0.0)	0(0.0)
Mixed/Other ethnic/Other black/Caribbean African	75(91.5)	1(1.2)	6(7.3)	71(86.6)	6(7.3)	5(6.1)	5(6.1)	75(91.5)	2(2.4)	51(62.2)	28(34.2)	3(3.7)	0(0.0)
Comparison (p-value, ϕ_c)	FET = 0.005, ϕ_c = 0.069			FET = 0.296, ϕ_c = 0.037			FET = 0.058, ϕ_c = 0.048			FET =0.001, ϕ_c = 0.070			
**Role in the university *n*(%)**													
Staff	1382(92.4)	2(0.1)	111(7.4)	1293(86.5)	78(5.2)	124(8.3)	67(4.5)	1398(93.5)	30(2.0)	1162(77.7)	320(21.4)	13(0.9)	0(0.0)
Students	491(92.0)	2(0.4)	41(7.7)	474(88.8)	21(3.9)	39(7.3)	27(5.1)	492(92.3)	14(2.6)	341(63.9)	177(33.2)	15(2.8)	1(0.2)
Comparison (p-value, ϕ_c)	FET = 1.000, ϕ_c = 0.006			χ^2 = 0.188, ϕ_c = 0.042			χ^2 = 0.627, ϕ_c = 0.022			χ^2 <0.001, ϕ_c = 0.150			
**Disability *n*(%)**													
Yes	51(98.1)	0(0.0)	1(1.9)	47(90.4)	1(1.9)	4(7.7)	4(7.7)	46(88.5)	2(3.9)	34(65.4)	18(34.6)	0(0.0)	0(0.0)
No	1782(92.1)	4(0.2)	149(7.7)	1690(87.3)	94(4.9)	151(7.8)	86(4.5)	1810(93.6)	38(2.0)	1449(74.9)	460(23.8)	25(1.3)	1(0.1)
Comparison (p-value, ϕ_c)	FET = 0.241, ϕ_c = 0.036			FET = 0.757, ϕ_c = 0.022			FET = 0.175, ϕ_c = 0.038			FET = 0.130, ϕ_c = 0.049			
**Staff role *n*(%)**													
Academic	690(91.4)	1(0.1)	64(8.5)	657(87.0)	41(5.4)	57(7.6)	22(2.9)	721(95.5)	12(1.6)	593(78.5)	157(20.8)	5(0.7)	0.(0.0)
Facilities and estates	132(95.7)	1(0.7)	5(3.6)	121(87.7)	5(3.6)	12(8.7)	19(13.8)	113(81.9)	6(4.4)	111(80.4)	26(18.8)	1(0.7)	0.(0.0)
Administration	230(92.7)	0(0.0)	18(7.3)	215(86.7)	9(3.6)	24(9.7)	11(4.4)	235(94.8)	2(0.8)	193(77.8)	54(21.8)	1(0.4)	0.(0.0)
IT services	76(92.7)	0(0.0)	6(7.3)	63(76.8)	7(8.5)	12(14.6)	1(1.2)	78(95.1)	3(3.7)	61(74.4)	20(24.4)	1(1.2)	0.(0.0)
Comparison (p-value, ϕ_c)	FET = 0.308, ϕ_c = 0.054			FET = 0.105, ϕ_c = 0.067			FET <0.001, ϕ_c = 0.137			FET = 0.712, ϕ_c = 0.034			

FET = Fisher's exact test, χ^2 = Chi Square test, ϕ_c = Cramer's V

For peer review only

For peer review only - http://bmjopen.bmj.com/site/about/guidelines.xhtml

For peer review only

STROBE Statement—Checklist of items that should be included in reports of ***cross-sectional studies***

	Item No	Recommendation	Page No
**Title and abstract**	1	(*a*) Indicate the study's design with a commonly used term in the title or the abstract	2
		(*b*) Provide in the abstract an informative and balanced summary of what was done and what was found	2
**Introduction**			
Background/rationale	2	Explain the scientific background and rationale for the investigation being reported	3
Objectives	3	State specific objectives, including any prespecified hypotheses	3
**Methods**			
Study design	4	Present key elements of study design early in the paper	4
Setting	5	Describe the setting, locations, and relevant dates, including periods of recruitment, exposure, follow-up, and data collection	4
Participants	6	(*a*) Give the eligibility criteria, and the sources and methods of selection of participants	4
Variables	7	Clearly define all outcomes, exposures, predictors, potential confounders, and effect modifiers. Give diagnostic criteria, if applicable	4
Data sources/ measurement	8*	For each variable of interest, give sources of data and details of methods of assessment (measurement). Describe comparability of assessment methods if there is more than one group	4
Bias	9	Describe any efforts to address potential sources of bias	4
Study size	10	Explain how the study size was arrived at	4
Quantitative variables	11	Explain how quantitative variables were handled in the analyses. If applicable, describe which groupings were chosen and why	4
Statistical methods	12	(*a*) Describe all statistical methods, including those used to control for confounding	4
		(*b*) Describe any methods used to examine subgroups and interactions	4
		(*c*) Explain how missing data were addressed	4
		(*d*) If applicable, describe analytical methods taking account of sampling strategy	n/a
		(*e*) Describe any sensitivity analyses	n/a
**Results**			
Participants	13*	(a) Report numbers of individuals at each stage of study—eg numbers potentially eligible, examined for eligibility, confirmed eligible, included in the study, completing follow-up, and analysed	5
		(b) Give reasons for non-participation at each stage	5
		(c) Consider use of a flow diagram	n/a
Descriptive data	14*	(a) Give characteristics of study participants (eg demographic, clinical, social) and information on exposures and potential confounders	5-6
		(b) Indicate number of participants with missing data for each variable of interest	5
Outcome data	15*	Report numbers of outcome events or summary measures	n/a
Main results	16	(*a*) Give unadjusted estimates and, if applicable, confounder-adjusted estimates and their precision (eg, 95% confidence interval). Make clear which confounders were adjusted for and why they were included	SF4

For peer review only - http://bmjopen.bmj.com/site/about/guidelines.xhtml

		(*b*) Report category boundaries when continuous variables were categorized	n/a
		(*c*) If relevant, consider translating estimates of relative risk into absolute risk for a meaningful time period	n/a
Other analyses	17	Report other analyses done—eg analyses of subgroups and interactions, and sensitivity analyses	n/a
**Discussion**			
Key results	18	Summarise key results with reference to study objectives	5-12
Limitations	19	Discuss limitations of the study, taking into account sources of potential bias or imprecision. Discuss both direction and magnitude of any potential bias	13
Interpretation	20	Give a cautious overall interpretation of results considering objectives, limitations, multiplicity of analyses, results from similar studies, and other relevant evidence	12-14
Generalisability	21	Discuss the generalisability (external validity) of the study results	12-14
**Other information**			
Funding	22	Give the source of funding and the role of the funders for the present study and, if applicable, for the original study on which the present article is based	18

*Give information separately for exposed and unexposed groups.

Note: An Explanation and Elaboration article discusses each checklist item and gives methodological background and published examples of transparent reporting. The STROBE checklist is best used in conjunction with this article (freely available on the Web sites of PLoS Medicine at http://www.plosmedicine.org/, Annals of Internal Medicine at http://www.annals.org/, and Epidemiology at http://www.epidem.com/). Information on the STROBE Initiative is available at www.strobe-statement.org.

For peer review only

BMJ Open

Student and staff views and experiences of asymptomatic testing on a university campus during the Covid-19 pandemic in Scotland: A mixed methods study

Journal:	*BMJ Open*
Manuscript ID	bmjopen-2022-065021.R1
Article Type:	Original research
Date Submitted by the Author:	23-Feb-2023
Complete List of Authors:	Bauld, Linda; The University of Edinburgh Usher Institute of Population Health Sciences and Informatics<br>Street, Alice; The University of Edinburgh School of Social and Political Science,<br>Connelly, Roxanne; The University of Edinburgh School of Social and Political Science<br>Bevan, Imogen; The University of Edinburgh School of Social and Political Science<br>Morlet Corti, Yazmin; The University of Edinburgh School of Social and Political Science<br>Baxter, Mats; The University of Edinburgh, Usher Institute<br>Stagg, Helen R.; The University of Edinburgh Usher Institute of Population Health Sciences and Informatics<br>Christison, Sarah; The University of Edinburgh School of Social and Political Science<br>Mulherin, Tamara; The University of Edinburgh Usher Institute of Population Health Sciences and Informatics<br>Sinclair, Lesley; University of York, Department of Health Sciences<br>Aitman, Tim; The University of Edinburgh MRC Institute of Genetics and Molecular Medicine, Institute of Genetics and Cancer
**Primary Subject Heading**:	Public health
Secondary Subject Heading:	Infectious diseases, Qualitative research, Health policy
Keywords:	PUBLIC HEALTH, COVID-19, Health policy < HEALTH SERVICES ADMINISTRATION & MANAGEMENT, Infection control < INFECTIOUS DISEASES, QUALITATIVE RESEARCH

SCHOLARONE™
Manuscripts

I, the Submitting Author has the right to grant and does grant on behalf of all authors of the Work (as defined in the below author licence), an exclusive licence and/or a non-exclusive licence for contributions from authors who are: i) UK Crown employees; ii) where BMJ has agreed a CC-BY licence shall apply, and/or iii) in accordance with the terms applicable for US Federal Government officers or employees acting as part of their official duties; on a worldwide, perpetual, irrevocable, royalty-free basis to BMJ Publishing Group Ltd ("BMJ") its licensees and where the relevant Journal is co-owned by BMJ to the co-owners of the Journal, to publish the Work in this journal and any other BMJ products and to exploit all rights, as set out in our licence.

The Submitting Author accepts and understands that any supply made under these terms is made by BMJ to the Submitting Author unless you are acting as an employee on behalf of your employer or a postgraduate student of an affiliated institution which is paying any applicable article publishing charge ("APC") for Open Access articles. Where the Submitting Author wishes to make the Work available on an Open Access basis (and intends to pay the relevant APC), the terms of reuse of such Open Access shall be governed by a Creative Commons licence – details of these licences and which Creative Commons licence will apply to this Work are set out in our licence referred to above.

Other than as permitted in any relevant BMJ Author's Self Archiving Policies, I confirm this Work has not been accepted for publication elsewhere, is not being considered for publication elsewhere and does not duplicate material already published. I confirm all authors consent to publication of this Work and authorise the granting of this licence.

For peer review only

Title	**Student and staff views and experiences of asymptomatic testing on a university campus during the Covid-19 pandemic in Scotland: A mixed methods study**

Author's names	Linda Bauld[1]<br>Alice Street[2]<br>Roxanne Connelly[2]<br>Imogen Bevan[1,2]<br>Yazmin Morlet Corti[1,2]<br>Mats Stage Baxter[1]<br>Helen R. Stagg[1]<br>Sarah Christison[2]<br>Tamara Mulherin[1]<br>Lesley Sinclair[3]<br>Tim Aitman[4]
Address for each author<br>Authors names and positions	[1]Usher Institute, College of Medicine and Veterinary Medicine, University of Edinburgh<br>[2]School of Social and Political Science, College of Arts and Humanities, University of Edinburgh<br>[3]Department of Health Sciences, University of York<br>[4] Institute of Genetics and Cancer, University of Edinburgh
Corresponding Author	Correspondence to: Linda.Bauld@ed.ac.uk<br>Usher Institute – University of Edinburgh<br>Doorway 1, Old Medical School<br>Teviot Place<br>Edinburgh EH8 9AG<br>Linda.Bauld@ed.ac.uk

Word Count	4600

For peer review only

For peer review only - http://bmjopen.bmj.com/site/about/guidelines.xhtml

Abstract

Objectives - To explore the acceptability of regular asymptomatic testing for SARS-CoV-2 on a university campus using saliva sampling for polymerase chain reaction (PCR) analysis, and the barriers and facilitators to participation.

Design - Cross-sectional surveys and qualitative semi-structured interviews.

Setting - City of Edinburgh, Scotland.

Participants - University staff and students who had registered for the testing programme (TestEd) and provided at least one sample.

Results - 522 participants completed a pilot survey in April 2021 and 1,750 completed the main survey (November 2021). 48 staff and students who consented to be contacted for interview took part in the qualitative research. Participants were positive about their experience with TestEd with 94% describing it as 'excellent' or 'good'. Facilitators to participation included multiple testing sites on campus, ease of providing saliva samples compared to nasopharyngeal swabs, perceived accuracy compared to lateral flow devices (LFDs) and reassurance of test availability while working or studying on campus. Barriers included concerns about privacy while testing, time to and methods of receiving results compared to LFDs, and concerns about insufficient uptake in the university community. There was little evidence that availability of testing on campus changed the behaviour of participants during a period when Covid-19 restrictions were in place.

Conclusions - Provision of free asymptomatic testing for Covid-19 on a university campus was welcomed by participants and the use of saliva-based PCR testing was regarded as more comfortable and accurate than LFDs. Convenience is a key facilitator of participation in regular asymptomatic testing programmes. Availability of testing did not appear to undermine engagement with public health guidelines.

Strengths and limitations of this study

- Mixed methods study to explore perceptions of a novel saliva-based PCR asymptomatic testing programme for Covid-19 designed to improve on LFDs in a screening context.
- Included two surveys six months apart and in-depth semi-structured interviews with a subsample of participants.
- Limited to the views and experiences of those who chose to take part and could not explore reasons for non-participation or compare the characteristics of participants with the university population as a whole.
- Findings may be transferable to other asymptomatic testing programmes for SARS-CoV-2 or other viruses on university campuses or in other educational settings and workplaces.

For peer review only - http://bmjopen.bmj.com/site/about/guidelines.xhtml

For peer review only

Introduction

The extent to which universities played a role in community transmission of SARS-CoV-2 was heavily debated in the UK in the early stages of the COVID-19 pandemic.[1,2] As in many other countries, higher and further education institutions had to pause non-essential teaching and research activities on several occasions, leading to long periods of online learning and many staff working from home. Essential campus activities continued throughout, however, and students moved between their term-time accommodation and other locations. In order to improve the safety of on-campus activities and reduce the risks of outbreaks, some experts recommended regular asymptomatic testing of students and staff alongside other public health measures.[3–5]

A few UK universities were early adopters of this approach, establishing their own pilot asymptomatic testing programmes involving either polymerase chain reaction (PCR)[6,7] or lateral flow devices (LFDs).[8,9] Early studies of these programmes found acceptability of asymptomatic testing among students to be high. However, uptake and adherence were found to be affected by anxiety[6] and concerns about the accuracy of tests, especially LFDs,[8,9] raising questions about students' long-term willingness to engage with regular testing. Government-funded asymptomatic COVID-19 testing in the form of LFDs first became available to all UK universities in December 2020 following concerns that a mass 'migration' of students over the winter break might lead to a rapid rise in cases.[10] This was offered to all students leaving and returning to campus. Evaluations of this testing programme found uptake among students to be low[11] and that concerns about accuracy were a prominent barrier to participation.[11,12] LFDs were rolled out to the general public from April 2021. Students and staff were then encouraged to test twice a week using LFDs. However, given their low sensitivity, several experts have queried the benefits and cost-effectiveness of mass asymptomatic LFD testing, especially during periods of lower viral prevalence in the community.[13–15]

The University of Edinburgh established an asymptomatic testing research programme, TestEd (www.ed.ac.uk/tested-covid), in January 2021. This aimed to improve on existing approaches to PCR testing in terms of acceptability and cost, and also provide a more accurate alternative to LFDs. TestEd involves a novel testing platform that uses pooled saliva-based testing by PCR, with a protocol adapted from an approach for nasopharyngeal swab testing.[16]

TestEd included surveys and interviews with participating staff and students to explore: the acceptability of regular PCR testing among students and staff, particularly involving an approach that was less invasive than nasopharyngeal swabbing; barriers and facilitators to participating in a regular university testing programme, including in the context of other testing methods being available; and whether participation in such a programme changed adherence to public health guidelines. We suggest that understanding staff and students' perceptions and experiences of TestEd's novel testing system can help to inform the design of effective regular asymptomatic testing programmes for Covid-19 or other disease outbreaks in educational and workplace settings in the future.

Methods

The TestEd programme

For peer review only - http://bmjopen.bmj.com/site/about/guidelines.xhtml

All University of Edinburgh students and staff who were coming onto campus were eligible to take part in TestEd on a voluntary basis and could sign up at any time. After joining, they were encouraged to provide twice-weekly saliva samples at one of the thirty testing centres located throughout the university. This involved spitting into a plastic cup, transferring the saliva to a tube and scanning their participant identifier and a barcode on the tube to register their sample. Samples were then collected from test sites and transferred to a university lab for PCR analysis. Participants normally received their test results within 24 hours by logging onto a secure portal with their university username and password. Between January 2021 and February 2022, 3,895 staff and 3,106 students registered and consented to participate. The programme tested just over 100,000 samples with more than 170 positive results during that period. A supermarket shopping voucher was provided to those who tested positive and sought a confirmatory PCR test from the NHS to assist with self-isolation. Participants were asked not to travel to campus to access testing, but instead to use TestEd while already there to study/work.

Design

Participants who consented to taking part in TestEd and who had provided at least one saliva sample were invited by email to participate in two online surveys using the Qualtrics tool, one (a pilot) carried out between 15 April and 30 April 2021 and the main survey between 8 November and 21 November 2021. The pilot and main surveys consisted of closed-ended and open-ended questions (See Supplementary File (SF) 1 and 2). The pilot survey was tested with three post-graduate students and amended following their feedback prior to distribution. No questions were compulsory. The number of eligible TestEd participants increased between the pilot and the main survey when students and more staff returned to campus for the 2021/22 academic year.

Semi-structured online interviews with participants were conducted between May 2021 and February 2022 (see Supplementary File 3). We were particularly interested in the views of participants who tested positive and aimed to interview more of this group than those who tested negative. We used purposive sampling to recruit participants from across the university and a wide range of demographic groups (university role, age, gender, ethnicity and disability) in order to ensure a diverse range of views and experiences were represented.

Ethical approval for both the surveys and interviews was received from the University of Edinburgh's Medical School Ethics Committee (EMREC) on April 1st 2021 Rec Ref: 20-EMREC-023_SA03.

Patient and Public Involvement

Volunteer students and staff were involved in contributing to the survey design and testing the questionnaire before the survey launched.

Analysis

Data from both surveys were extracted from Qualtrics and exported to Stata. Variable recoding was undertaken to enable appropriate cell sizes for statistical analysis and to avoid statistical disclosure (>15). Variables indicating gender, age, ethnicity, disability and university role were recorded. Recoding as missing values was applied for all variables with 'not applicable' and 'prefer not to say' responses. Due to small numbers, the 'non-binary'

For peer review only - http://bmjopen.bmj.com/site/about/guidelines.xhtml

category of gender was recoded as missing, and the categories of ethnicity were grouped as shown in Table 1. Responses to the survey questions were examined using descriptive statistics (e.g., frequencies and percentages). We conducted chi square tests and Fisher's exact tests where appropriate in order to investigate patterns between sociodemographic characteristics and responses to the survey. While some of these tests were statistically significant, effect sizes were very low (Cramér's ≤ 0.1) indicating only very weak patterns of association. These results are not presented in the main text and are available instead in a supplementary file (see Supplementary File 4). For questions that were duplicated in the two surveys, where participants had responded to these both times it was possible to analyse changes in attitudes and experiences between the two time points.

Qualitative data from open-ended survey questions and semi-structured interviews were analysed through a thematic coding approach by SC, IB and AS using NVivo software (versions 1.3 and 1.6.1). The content of the survey questions provided an initial coding structure, which was revised during analysis to reflect additional issues and topics raised in the results. Coding of semi-structured interviews was inductive, reflecting the more open-ended nature of the interviews. The interviews addressed a wide range of topics and for this article we only analysed a subset of results related to acceptability, perceptions and experiences of the TestEd programme. Initial coding was carried out by SC (survey) and IB (interviews) and quality checked by AS who read all results and interview transcripts. Coding categories were collectively reviewed, discussed and revised as a team before a final coding structure was agreed for each dataset. Codes were collectively organised into themes by SC, IB and AS during team analysis meetings. The team discussed and analysed commonalities, overlaps, and differences between the codes to derive common themes. A shared table on Microsoft Sharepoint was used to visualise relationships between example data extracts, codes and themes to ensure that clear connections could be drawn between the analysis and the data and to check where thematic categories were too narrow or broad. Themes were collectively reviewed against a sample of the surveys to check for coherence and areas of overlap between themes, with iterative changes made to the thematic scheme. The agreed set of themes are reflected in the subheadings below.

Results

Out of 760 eligible participants who had provided at least one saliva sample when the pilot survey was distributed, 548 responses were received (72%), 522 of which were complete (69%). For the main survey, out of 4,512 eligible participants, 2,995 responses were received (66%), 1,750 of which were complete (58%). 300 participants responded to both surveys. 70 participants were invited for interview, 48 of whom were successfully contacted and took part.

Participant characteristics

Participant characteristics are shown in Table 1. When compared to TestEd participants overall, the survey population included more staff members and participants identifying as female (data not shown).

Table 1: Participant characteristics

For peer review only - http://bmjopen.bmj.com/site/about/guidelines.xhtml

Participant characteristics	Main survey n (%)	Pilot n (%)	Interviews
**Overall**	**1750**	**300**	**48**
**Gender**			
Female	996 (58%)	194 (65%)	26
Male	721 (41%)	103 (34%)	21
Non-binary	21 (1%)	2 (<1%)	1
Other	1 (<1%)	0 (<1%)	0
Preferred not to disclose	33 (<1%)	3 (<1%)	0
**Age (years)**			
≤19	41 (2%)	0 (0%)	4
20–29	512 (29%)	77 (26%)	13
30–39	403 (23%)	73 (24%)	11
40–49	336 (19%)	54 (18%)	9
50–59	335 (19%)	60 (20%)	5
≥60	123 (7%)	36 (12%)	6
**Ethnicity**			
British/Irish/Other white	1570 (90%)	272 (92%)	33
Asian/Indian/Pakistani/Bangladeshi/Chinese/Other Asian	98 (6%)	13 (4%)	9
Mixed/Other ethnic/Other black/Caribbean African	71 (4%)	11 (4%)	6
Preferred not to disclose	11 (<1%)	1 (<1%)	0
**Role in the university**			
Staff	1247 (72%)	248 (83%)	28
Student	482 (28%)	52 (17%)	20
Preferred not to disclose	21 (<1%)	0(<1%)	0
**Disability**			
Yes	46 (3%)	6 (2%)	5
No	1651 (97%)	284 (98%)	40
Preferred not to disclose	53(<1%)	10 (<1%)	3

*Original values are retained; the analysis groups responses <15 into categories.
** Sociodemographic characteristics were collected at TestEd registration.

For peer review only

Reasons for participating

Overall, survey participants were positive about their experience with TestEd. 74% rated the experience as 'excellent' and 24% as 'good' in the main survey (see SF4). Those who participated in both the pilot and main surveys maintained enthusiasm for the programme over time, with little change in responses.

Survey responses indicated that 'knowing [their own] Covid-19 status in the absence of symptoms' was the most important reason for participation (38%), followed by prevention of 'passing on infection to family and friends' (32%). For 18% of respondents the most important reason was 'to contribute to scientific research on Covid-19', and for 11% this was 'to prevent passing on infection to other colleagues/students on campus if I am positive'.

Interview participants similarly emphasised their desire to protect family and friends beyond the university community as being a primary motivation for joining the programme. While knowing their own Covid-19 status was considered important, this was often linked to the benefit of protecting others inside or outside the university, rather than viewing these as separate benefits of testing. Some interview participants described previous negative personal experiences of Covid or their witnessing of Covid or Long Covid symptoms in friends and family as a motivation to test, to prevent passing on the infection to others. Some interview participants also emphasised the heightened need for testing post-vaccination, when symptoms might be mitigated but one might still be infectious to others. The rationale of contributing to scientific research often emerged as an additional but secondary concern for interviewees. Other factors that interviewees suggested motivated them to join TestEd included the perceived need to follow government or institutional guidance; support and encouragement from the institution to take part; influence from peers; perceptions of risk; and, in a few select cases, the experience of Covid-like symptoms.

Testing method

Survey participants found the simple spit test easy to administer and less invasive compared to standard PCR or LFD swab-based tests. They found the process of providing a saliva sample to be fairly quick: for 42% of respondents, it took only 2–5 minutes out of their day; 41% indicated that it took just 1–2 minutes.

However, the saliva testing was not without problems. A few participants indicated that it could be difficult to produce enough saliva to provide a viable sample. This was also raised in interviews. Staff and students who signed up to TestEd were asked not to eat or drink for 30 minutes before testing. Some survey participants described this as a limitation, indicating that they would find it more convenient to provide a sample during their lunch or coffee breaks. There were also some issues with the privacy of sample collection booths, with some people feeling uncomfortable spitting into a cup when they could be observed. The booths did have sides but were located in public venues on campus. Table 2 reports a selection of participant views on the testing method.

Table 2: Views on the TestEd testing method

Facilitators	Barriers
'It's non-invasive, simple, and involves no discomfort whatsoever. This is a huge benefit in making a testing regime attractive to its users.'<br><br>*'A much less invasive form of testing compared to lateral flow tests! Given how invasive they are, I also doubt many are correctly using other lateral flow tests, rendering the results inaccurate.'*<br><br>*'Saliva samples are very easy to provide (and non-intrusive) and I was concerned that I may not have been doing the lateral flow nasal and throat swab correctly hence my preference for saliva sampling.'*	*'If the spit sample is not of a high enough volume it will not work. So sometimes my results may have been invalid. I have to work up spit in my mouth for a couple of minutes prior.'*<br><br>*'Sample can be given easily on the way to school. The only inconvenience comes from the time taken to collect enough saliva for the sample and finding a time where I have not eaten or drank in the past 30 minutes.'*<br><br>*'I'd prefer a privacy curtain that I could pull behind me when I'm in the booth. I feel very exposed when spitting in the cup in the middle of the library, especially if things get*

For peer review only - http://bmjopen.bmj.com/site/about/guidelines.xhtml

'It is very convenient and much more accessible than doing a tonsil/nostril swab. Saliva spit tests increase my motivation to test.'	*messy!'*<br><br>*'I felt very much under pressure to do this spitting thing, and I couldn't perform basically, so I just took everything with me in the office and I was like, "I'm nice and safe here." There was nobody around, but still it felt very weird to have to spit.'*

Convenience

The majority of survey participants also indicated that it was either 'very convenient' (68%) or 'convenient' (26%) to provide a sample as part of their work/study schedules (see SF4). Participants touched on issues of convenience at multiple points in the testing process, from experiences of sample collection, to navigating the TestEd IT systems, to the receipt of results.

Participating in TestEd was reported to be convenient due to the number and location of the test centres, which were in many cases located within buildings where participants worked. Participants also described how the drop-in element made participating easier as tests could be taken at any time without appointment or prior booking. Interview responses revealed that perceptions of convenience were often linked to individual work/study patterns. Interviewees with a regular on-campus working schedule, and particularly those with a testing site inside the same building as their office or laboratory, reported developing a routine testing schedule (e.g., on the way to work or during a morning break) by comparison with students and staff with more varying schedules and who worked across multiple locations or between home and campus, who found it harder to establish a testing routine.

Some expressed a desire for longer opening hours, better communication of opening hours, or complained that some centres were not open as advertised or could not be accessed without the correct security clearance on their staff/student card. Many participants also found the testing programme's IT systems cumbersome. In a few cases people reported that the bar code on the test tube did not work. Many participants described the process for logging in and accessing results through the online system to be inconvenient and expressed a preference for the NHS system of sending results directly via SMS and email. While some found the turnaround times to be 'quick', other participants described turnaround times to be inconsistent or too long. Further probing in interviews revealed that perceptions of test turnaround times as either quick or slow were often shaped by comparison with another form of testing (e.g., LFD, NHS-administered PCR test), and by specific time-sensitive motivations for testing on that occasion. Table 3 presents some of these views about perceived convenience.

Table 3: Convenience of TestEd

Facilitators	Barriers
'There is a testing station at my university accommodation so it is very easy to get to and provide a sample.'<br><br>*'The booths are close to my work area. The*	*'I'm either out on site so I'm at [campus site 1], or I'm at [site 2] – it then becomes a question of, "Do I have the time to drive from those locations back to [site 3] for a ten-minute spit test?" So at times you just*

For peer review only

process is quick, so you can easily fit in your schedule. Also it's self-administered and open all the time, so you can test anytime.'<br><br>*'Station is set up throughout working hours, drop-in nature means can give a sample at a time that suits in my clinical day.'*	*have to sacrifice the test and not go.'*<br><br>*'It seems unnecessary to have to log in to get my results once notified. The NHS system doesn't require this: the text message and email both contain the test result.'*<br><br>*'Sometimes the results take longer to come through than other times – it can be hard to know how long to expect to wait for results.'*

Concerns about Covid-19 on campus

Most survey participants indicated some level of concern about catching Covid-19 on campus: 21% were 'very concerned'; 33% were 'moderately concerned'; and 22% 'somewhat concerned'. Many expressed concerns about the return of students and the re-introduction of in-person teaching, which were perceived to have led to increased mixing on campus. A common concern was the lack of adequate ventilation in teaching rooms and the ability to maintain social distancing in shared spaces:

'I slightly worry that I may catch Covid-19 from a student in class, as I spend a good amount of time with my students and not all our rooms are as well ventilated as I'd like them to be.'

'There is obviously some increased risk due to meeting more people and using more shared facilities than if working at home.'

Despite these concerns, many survey participants perceived the likelihood of infection on campus to be lower than elsewhere. While some felt that there was low compliance with safety measures, others believed that the university's infection control measures were robust and effective. Some of these different perspectives of safety on campus may be related to a participant's position or role within the university – for example, working alone in single-occupancy offices vs roles that involved more contact with others at work or while studying:

'I felt that the safety precautions in operation at work (mask-wearing, handwashing, social distancing) were adequate.'

'[I am] usually based in my office which is single occupancy – risk here is less than going to the shops.'

Reassurance

The majority of survey participants (87%) indicated that the availability of the TestEd programme made them feel reassured about working or studying on campus. Levels of reassurance increased over time among participants who took part in both the pilot and main surveys, rising from 90% to 94%.

In some cases, TestEd provided reassurance about participants' own health, but it was more common for participants to connect that reassurance to their sense of personal

For peer review only - http://bmjopen.bmj.com/site/about/guidelines.xhtml

responsibility for the wellbeing of others. Responses to open-ended survey questions and interviews indicated that perceived levels of participation among others influenced how reassured individuals felt. Those who felt that there were high levels of participation, particularly among close colleagues, indicated that this made them feel reassured, while those who thought those around them on campus were not taking part had more concerns. One factor that influenced how participants perceived participation was the number of samples which they noticed had been provided at test sites. Concerns about low participation led some participants and interviewees to reflect on the efficacy of a workplace testing programme that relied on voluntary participation. Table 4 highlights a number of these responses.

Table 4: Reassurance

Facilitators	Barriers
'[I] am severely immunocompromised so worried about all contact. Knowing the majority are being tested regularly has eased these concerns.'<br><br>*'I don't think it makes a significant difference to my risk of catching Covid on campus, but it reduces the risk that I might unknowingly pass on Covid.'*<br><br>*'It's good that my colleagues and I have access to a free and accurate testing service, so I am confident that I am not unwittingly spreading COVID.'*<br><br>*'Most of my direct colleagues are using TestEd as well. Reassuring when working in the same room.'*<br><br>*'Knowing that colleagues were also participating in the programme provided a certain level of reassurance, along with my own results of course.'*<br><br>*'Because as the staffing levels have increased, I see an increasing amount of provided samples in the collection trays so I am confident people are getting regularly tested.'*	*'It's unclear to me how many staff and students are taking part and how regular[ly] they are testing, so it doesn't necessarily make me feel more reassured about catching Covid-19 while at work.'*<br>*'On one hand it is definitely a positive, but on the other I often see how few samples have been submitted when I go to drop off my own. It doesn't seem like as many people have taken advantage of the availability of the system as could have.'*<br><br>*'There seems to be very little take up on it – maybe only 15–20 samples when I go so [I am] concerned a lot of people, especially students, aren't doing it. I'm aware of outbreaks on campus but we're not officially informed of that – I think we should be.'*<br><br>*'If everyone on campus was required to enrol in TestEd to work/study on campus, I would feel safer. Voluntary enrolment is not good enough to ensure safety.'*<br><br>*'I'd feel more assured if it was compulsory for all who use campus. Some of my students think they are immune and are less risk-averse as a result.'*

Accuracy

The vast majority of survey participants (92%) agreed that the results they received from TestEd were accurate. Among those who completed both the pilot and the main survey, 93% indicated in both surveys that they believed their results from TestEd were accurate.

For peer review only - http://bmjopen.bmj.com/site/about/guidelines.xhtml

In open-ended survey responses and interviews, participants emphasised their trust in the scientists involved in developing the TestEd programme as a basis for their belief in the accuracy of the test results. Participants also described how they had more faith in the PCR testing used for TestEd compared to LFDs. Some also reported that they felt that the saliva-based tests were likely to be more accurate, as the sample collection process was less prone to user error compared with self-administered swabs.

While participating in TestEd, many people were also using other testing methods, most commonly LFDs that were freely available in a variety of venues, including on campus. In the case of a positive TestEd result, all participants interviewed carried out a confirmatory PCR test through the NHS so that a positive test picked up in the study could be formally reported, allowing for contact tracing by the NHS. Testing positive via this confirmatory NHS test also confirmed for many that TestEd's methods were accurate. Interviewees also reported using LFDs either to confirm a positive TestEd result, or to check the accuracy of LFDs compared to PCR.

For the small proportion of participants who were unsure about the accuracy of TestEd results, open-ended responses indicated that more information regarding the effectiveness of saliva-based testing could provide reassurance. Some of this concern over accuracy was linked to the novel nature of the approach, with several participants stating that they felt there was a lack of knowledge regarding the effectiveness of saliva-based testing, or that the programme was an experimental study to trial this type of testing methodology (see Table 5).

Table 5: Accuracy

Facilitators	Barriers
'Because I trust the science behind it and I don't believe that it would have been rolled out university-wide if the university and the people behind TestEd were not confident that it would work.'<br><br>*'I understand TestEd used a PCR test which the NHS says is more accurate than a lateral flow test.'*<br><br>*'The quality of the sample provided is independently verified by the TestEd research team. Providing a saliva sample is also more straightforward and likely more error-free.'*<br><br>*'I mean the PCR test from the NHS was positive as well so I'm pretty sure it [TestEd] was [accurate]. With not having any symptoms, and then I got a positive, I might have been, "Oh, I'm not 100% sure." But having both tests positive, I'm pretty sure it has been accurate.'*	*'I am unsure about the effectiveness of the saliva as compared to the nasal swab, and have not seen data to show that. I also don't know if there are therefore not a lot of false negatives.'*<br><br>*'Haven't heard of a positive result yet, I haven't seen any information of a direct comparison of this test and the [nasal] swab test so I would trust a swab test more.'*<br><br>*'PCR tests are the most reliable – although the saliva samples are obviously part of a trial so a bit of an unknown, but still feel confident it will pick up most positives, and probably more accurately than a lateral flow.'*

Compliance with public health guidelines

For peer review only - http://bmjopen.bmj.com/site/about/guidelines.xhtml

Respondents were asked whether they had changed their approach to the public health guidelines that were in place at the time of the study (i.e., social distancing and face coverings) at work or study since they joined TestEd. The majority (93%) indicated that they had not changed their approach. Only 5% reported that they had, and 2% did not know. Responses to this question were similar between the pilot and main survey.

Among the small number of participants who indicated that they had changed their behaviour, some participants reported feeling more relaxed with regard to guidelines. In some cases this made them less adherent and in other cases it made them more confident to mix with others within the guidelines. Others who reported changing their behaviour following participation in TestEd explained that the testing programme had resulted in them following guidelines more stringently, for example with reference to wearing face masks:

'I was careful before as I wore FFP2 masks when in enclosed spaces. I am more reluctant to visit crowded public spaces as I worry that I could then test positive.'

'Am less worried about interacting with friends and family given negative tests, so I see more people if I've been regularly testing.'

'I confess I am a little less strict than before in following the guidelines. I sometimes forgot I do not wear a mask. This may be due to the fact that I feel less worried about catching it.'

In interviews, all participants who had tested positive reported having booked a confirmatory test through the NHS, to have informed their workplace, and to have fully complied with self-isolation guidelines. However, some also indicated challenges, including the effects on others of their decision to self-isolate, financial consequences, impacts on personal wellbeing, and a reliance on their own social networks for emotional support and provisions during the isolation period. Some interviewees, particularly students, highlighted issues such as taking out the rubbish, accessing meals, and negotiating spaces with other members of a household who had not tested positive:

'I was kind of really bored in my room, because in my flat there's one other person so I tried my best not to go in the kitchen or the living room. The only true place I can go is my bedroom and the bathroom. So it was quite difficult because I felt like I was also inconveniencing her; if I wanted water or food or something she had to bring it to my door. Although I am more than capable of making myself a cup of tea, I didn't want to go into the kitchen and accidentally contaminate things.'

'I didn't want to trouble other people to carry all my groceries for me. And it's not enough to stack up to the minimum delivery. So I just ended up trying to make do, asking people if they could just buy one or two things for me and stuff. So that's one very big inconvenience.'

Discussion

This study adds to evidence from previous research that routine asymptomatic testing for SARS-CoV-2 can be introduced on university campuses in a way that is accessible and acceptable to staff and students. Although TestEd was used by a minority of students and university employees during the study period, the programme was introduced at a time when working from home guidance was in place and footfall on campus was low.[2] For those that regularly participated, enthusiasm for the availability of free asymptomatic testing was maintained over time.

Reasons for taking part included participants wanting to know their own Covid status and avoiding passing the virus onto others, which confirms findings on attitudes to Covid-19

For peer review only - http://bmjopen.bmj.com/site/about/guidelines.xhtml

testing from studies in multiple countries.[17] Despite TestEd being a workplace programme, concern for others was not necessarily limited to colleagues and instead also related to protecting vulnerable friends and relatives off-campus. Early in the pandemic it was suggested that highly interconnected social networks inside and outside university make it a high-risk environment.[18] Our findings suggest that university staff and students are aware of these risks and are willing to take active measures to reduce them.

Previous research has shown that concerns about physical discomfort and the capacity to perform nasopharyngeal swab-based sample collection are barriers to participation in testing.[19,20] TestEd involved a novel saliva-based sampling method for PCR testing, avoiding nasal pharyngeal swabs. Participants reported that this was a more comfortable form of testing. However, there were some concerns about producing enough saliva and around privacy while spitting into a cup at testing sites. Other university-based studies have found similar concerns among participants about their ability to perform saliva-based testing.[21,22] One study that compared saliva- and swab-based testing methods found no consensus among participants on the preferred method.[6] While saliva-based testing has some advantages over swab testing in terms of physical comfort, our findings show that it can also introduce new challenges and concerns for participants.

The convenience of testing was something participants valued, confirming findings from other studies that have found convenience to be a key facilitator for Covid-19 testing uptake.[8,9,22,23] Aspects of the TestEd programme that were found to be convenient included the sample collection method and the quantity and accessibility of sample collection points across campus. However, in some instances negative experiences of IT systems used to sign up, submit samples and access results negatively affected perceptions of convenience. Having to wait for results (compared with the quick turnaround time for LFDs) was also a disadvantage. Our findings show that the perceived convenience of a particular testing method varied in relation to the context for and purpose of testing. Because TestEd was in place when other forms of free testing were available (via the NHS for those with symptoms and LFDs for asymptomatic testing in wider society) it is unsurprising that participants combined different kinds of tests according to which was deemed most convenient at a particular moment.

Participation in TestEd was reported as being reassuring for participants, consistent with previous research on Covid-19 testing in education settings[6,8,22] and workplaces.[24,25] Our results found that this reassurance was, however, mediated by perceptions about levels of participation in the testing programme by others. Participants were sensitive to the question of whether they were part of a larger testing community, in part because they understood that the effectiveness of the programme as a public health screening tool depended on others also taking part.

Previous studies have found that concerns about the accuracy of LFDs can be a barrier to participation in testing.[7,9,11] We found that survey and interview participants were aware of differences in the sensitivity of PCR compared to LFDs, and perceived PCR to be a more accurate testing method. Saliva-based self-testing was also perceived to be more accurate than self-testing with a nasopharyngeal swab. Participating in a programme developed by university scientists provided some reassurance that testing results were likely to be accurate.

For peer review only - http://bmjopen.bmj.com/site/about/guidelines.xhtml

There was limited evidence that testing resulted in changes in behaviour among those who participated, for example leading to increased confidence to socialise, both within and outside existing guidelines. We also did not find evidence that the availability of on campus testing made participants more cautious or aware of Covid-19 guidelines, but it is likely that those engaging with TestEd were already aware of and trying to follow these guidelines. Similar findings have been reported for other university-based studies[6,8] and for workplace studies of antibody testing.[25] In line with findings from previous studies[6,26,27] participants experienced daily challenges during self-isolation, such as when isolating from other members of the household,[28] but this did not affect self-reported compliance with guidelines.

We collected information about participant characteristics but did not identify any significant differences in survey responses between groups, although our samples may have been too small to examine relevant characteristics (such as disabilities or ethnicity) in detail.

An important limitation of this study is we could not assess what proportion of eligible students and staff accessed TestEd, because registration was intended for those that were coming onto campus, something that was not routinely monitored particularly as 'working from home' guidance varied at different stages of the pandemic and the study period. Many registered students and some staff worked entirely from home (including in other parts of the UK and overseas) throughout the period when the study was taking place.

In addition, the study did not include the views of staff or students who did not participate in TestEd, despite visiting campus during the study period. There were varying public health regulations and guidance in place over the period of the research[2] and limited available information about footfall on campus, given the size and complexity of a large university. It is therefore difficult to assess how many staff and students would have used the programme if everyone eligible to do so had signed up. In order to begin to understand reasons for non-participation in TestEd we have recently engaged with the University of Edinburgh student panel, a group of 250 students designed to be representative of the student population. While almost all of those who responded to our brief online questionnaire to the panel (n=76, 30% response rate) had heard of TestEd, most chose not to participate because they didn't get round to registering, preferred not to know if they had Covid, or used LFDs instead.

Engagement with TestEd is voluntary, meaning that the participant population may differ from the student and staff population as a whole. We could not explore further differences between the TestEd population and the university population due to a lack of available data. Survey participants may also differ from the wider population of TestEd programme participants. The survey response rates were reasonably high (72% for the pilot survey and 66% for the main survey). However, when comparing the characteristics of the survey respondents to all TestEd participants we noted some differences, for example that there were more women amongst the survey respondents. There may therefore be biases in the survey responses due to the nature of the survey sample.

Conclusion

For peer review only - http://bmjopen.bmj.com/site/about/guidelines.xhtml

Despite alternative testing options being available in the community at the time of the research, our results indicate that an asymptomatic SARS-CoV-2 testing programme designed specifically for university staff and students was acceptable and was positively received by those who took part. Provision of multiple testing sites across campus and the ease of saliva sampling compared to swabs were facilitators to participation, as were perceptions about the accuracy of results from PCR testing compared with LFDs. Potential barriers to participation included concerns about privacy when providing a sample; difficulty in accessing and using IT systems; time to receiving results; and concerns about the extent to which the testing would reduce the risk of outbreaks on campus in the case of low levels of participation in the programme. Perceptions of convenience shaped facilitators and barriers to participation at every stage of the testing process. Availability of testing did not appear to undermine protective behaviours among participants to follow Covid-19 guidelines. These findings suggest that saliva-based PCR asymptomatic testing offers an acceptable and alternative and/or complement to LFD asymptomatic testing on university campuses. Future studies should explore reasons for non-participation in testing programmes in similar workplace or educational settings.

Summary

Section 1: What is already known on this topic

- Pilot studies have found high levels of acceptability of asymptomatic Covid-19 testing programmes among UK university staff and students but have not established whether or not acceptability can be sustained over longer time-periods.
- User concerns about the accuracy and discomfort of self-testing with LFDs are known barriers to participation in Covid-19 testing programmes.
- There is limited evidence as to whether regular asymptomatic testing encourages behavioural change in relation to Covid-19 protective behaviours and compliance with guidelines.

Section 2: What this study adds

- Regular asymptomatic saliva-based PCR testing is acceptable to university staff and students.
- The acceptability of voluntary asymptomatic testing programmes depends on participant perceptions of test accuracy and overall participation levels, and on experiences of testing convenience and comfort.
- We did not find evidence in our survey or interview data to suggest that participation in asymptomatic testing leads to non-compliance with public health guidelines for protective behaviours.

Ethics Statements

Patient consent for publication
Not applicable.

For peer review only - http://bmjopen.bmj.com/site/about/guidelines.xhtml

Ethics approval

This study involves human participants and approval was obtained from the University of Edinburgh's Medical School Research Ethics Committee (Ref: 20-EMREC-023_SA03). Participants gave informed consent to participate in the study before taking part.

Acknowledgements

We would like to thank Professors Neil Turok and Nick Gilbert (TestEd co-investigators) for their support for the survey and qualitative research elements. In addition, Professor Jonathan Seckl, Senior Vice Principal at the University of Edinburgh, who facilitated University pump priming funding for setting up the programme and assisted with liaising with key University staff and student networks. Kathryn Carruthers was TestEd project manager when the study took place and her assistance was invaluable. Finally, we thank the University of Edinburgh staff and students who participated in the research.

Contributor Statement

LB, AS, HS, RC and TA contributed to the design of this study. LB and TA initiated the project. LS and TM project managed the research. Statistical analysis was done by YM and MB supported by RC and HS. Qualitative analysis was conducted by IB and SC supported by AS. All authors contributed to the manuscript and read and approved the final manuscript. They have all read and approved the final manuscript.

Competing Interests

LB is Chief Social Policy Adviser to the Scottish government (part-time secondment) and chairs the Universities and Colleges Advisory Group, a subgroup of the Chief Medical Officer of Scotland's Advisory Group on Covid-19.

HS has received payment from the Scottish Parliament for advising the COVID and COVID recovery committees.

AS receives funding from the European Research Council (Grant Number 715450) for Investigating the Design and Use of Diagnostic Devices in Global Health, and holds positions on the Royal Anthropological Institute Medical Committee (unpaid), and the Wellcome Trust Career Development Committee (paid).

TA receives internal support from the University of Edinburgh. As the founder and director of BioCaptiva (a liquid biopsy company unrelated to the present study), he receives consulting fees. Additionally, he has received travel expenses for the Biomarkers UK Congress, Oxford Global, November 2021, and Liquid Biopsies, Global Engage conference, December 2021. TA is the Regional Champion for Scotland for the Academy of Medical Sciences, and sits on the Genomics England Scientific Advisory Committee, European Research Council advanced grant panel for genetics. He is also a Fellow of the Royal College of Physicians of London and Edinburgh, and trustee and director of the PHG Foundation.

Funding statement

For peer review only - http://bmjopen.bmj.com/site/about/guidelines.xhtml

This work was supported by UKRI Research Grant MR/W006243/1 and the University of Edinburgh. Additional support for manuscript preparation was from the DiaDev project funded by the European Research Council (ERC) European Union's Horizon 2020 Research and Innovation Programme, grant agreement 71540.

Data sharing statement

Survey data relevant to the study is accessible from the University of Edinburgh's open access DataShare: https://doi.org/10.7488/ds/3802

References

1 SAGE. SAGE Paper on Further and Higher Education. *Sage* 2020;**46**. https://assets.publishing.service.gov.uk/government/uploads/system/uploads/attachment_data/file/903321/S0599_SAGE_Paper_on_Further_and_Higher_Education_ORIGINAL.pdf (accessed 26 Apr 2022).

2 Scottish Government. Coronavirus (COVID-19): universities, colleges and community learning and development providers. 2022. https://www.gov.scot/publications/coronavirus-covid-19-universities-colleges-and-community-learning-and-development-providers/ (accessed 26 Apr 2022).

3 Yamey G, Walensky RP. Covid-19: Re-opening universities is high risk. *BMJ* 2020;**370**.https://doi.org/10.1136/BMJ.M3365.

4 Independent SAGE. Independent SAGE Statement on Universities in the Context of SARS-CoV-2. The Independent SAGE Report 11. Published Online First: 2020. www.independentSAGE.org (accessed 30 Mar 2022).

5 SAGE. Multidisciplinary Task and Finish Group on Mass Testing: Consensus Statement for SAGE. *Sage* 2020;**53**. https://assets.publishing.service.gov.uk/government/uploads/system/uploads/attachment_data/file/914931/s0712-tfms-consensus-statement-sage.pdf.

6 Blake H, Corner J, Cirelli C, *et al.* Perceptions and Experiences of the University of Nottingham Pilot SARS-CoV-2 Asymptomatic Testing Service: A Mixed-Methods Study. *Int J Environ Res Public Health* 2021;**18**:1–26. https://doi.org/10.3390/ijerph18010188.

7 Gillam TB, Cole J, Gharbi K, *et al.* Norwich COVID-19 testing initiative pilot: evaluating the feasibility of asymptomatic testing on a university campus. *J Public Health (Oxf)* 2021;**43**:82–8. https://doi.org/10.1093/pubmed/fdaa194.

8 Wanat M, Logan M, Hirst JA, *et al.* Perceptions on undertaking regular asymptomatic self-testing for COVID-19 using lateral flow tests: A qualitative study of university students and staff. *BMJ Open* 2021;**11**. https://doi.org/10.1136/bmjopen-2021-053850.

For peer review only - http://bmjopen.bmj.com/site/about/guidelines.xhtml

9 Hirst J, Logan M, Fanshawe TR, *et al.* Feasibility and Acceptability of Community COVID-19 Testing Strategies (FACTS) in a University Setting. *SSRN Electronic Journal* 2021;1–8. https://doi.org/10.2139/ssrn.3840101.

10 Department for Education. Christmas guidance set out for university students. 2020. https://www.gov.uk/government/news/christmasguidance-set-out-for-university-students.

11 French CE, Denford S, Brooks-Pollock E, *et al.* Low uptake of COVID-19 lateral flow testing among university students: a mixed methods evaluation. *Public Health* 2022;**204**:54–62. https://doi.org/10.1016/j.puhe.2022.01.002.

12 Jones LF, Batteux E, Bonfield S, *et al.* Durham University students' experiences of asymptomatic COVID-19 testing: a qualitative study. *BMJ Open* 2021;**11**:e055644. https://doi.org/10.1136/bmjopen-2021-055644.

13 Raffle AE, Pollock AM, Harding-Edgar L. Covid-19 mass testing programmes Should be modelled on successful screening programmes. *BMJ* 2020;**370**:10–1. https://doi.org/10.1136/bmj.m3262.

14 Wise J. Covid-19: Lateral flow tests miss over half of cases, Liverpool pilot data show. *BMJ* Published Online First: 2020. https://doi.org/10.1136/bmj.m4744.

15 Mahase E. Covid-19: Universities roll out pooled testing of students in bid to keep campuses open. *BMJ* 2020;**370**:m3789. https://doi.org/10.1136/bmj.m3789.

16 Reijns MAM, Thompson L, Acosta JC, *et al.* A sensitive and affordable multiplex RT-qPCR assay for SARS-CoV-2 detection. *PLoS Biol* 2020;**18**:1–20. https://doi.org/10.1371/journal.pbio.3001030.

17 Bevan I, Stage Baxter M, Stagg HR, *et al.* Knowledge, attitudes, and behavior related to covid-19 testing: A rapid scoping review. *Diagnostics (Basel)*. 2021;**11**. https://doi.org/10.3390/diagnostics11091685.

18 Nixon E, Trickey A, Christensen H, *et al.* Contacts and behaviours of university students during the COVID-19 pandemic at the start of the 2020/2021 academic year. *Sci Rep* 2021;**11**:1–13. https://doi.org/10.1038/s41598-021-91156-9.

19 Zimba R, Kulkarni S, Berry A, *et al.* SARS-CoV-2 testing service preferences of adults in the United States: Discrete choice experiment. *JMIR Public Health Surveill* 2020;**6**:1–7. https://doi.org/10.2196/25546.

20 Kernberg A, Kelly J, Nazeer S, *et al.* Universal Severe Acute Respiratory Syndrome Coronavirus 2 (SARS-COV-2) Testing Uptake in the Labor and Delivery Unit: Implications for Health Equity. *Obstet Gynecol* 2020;**136**:1103–8. https://doi.org/10.1097/AOG.0000000000004127.

21 Ehrenberg AJ, Moehle EA, Brook CE, *et al.* Launching a saliva-based SARS-CoV-2 surveillance testing program on a university campus. *PLoS ONE* 2021;**16**:1–20. https://doi.org/10.1371/journal.pone.0251296.

For peer review only - http://bmjopen.bmj.com/site/about/guidelines.xhtml

22 Watson D, Baralle NL, Alagil J, *et al.* How do we engage people in testing for COVID-19? A rapid qualitative evaluation of a testing programme in schools, GP surgeries and a university. *BMC Public Health* 2022;**22**:1–11. https://doi.org/10.1186/s12889-022-12657-4.

23 Blake H, Knight H, Jia R, *et al.* Students' views towards Sars-Cov-2 mass asymptomatic testing, social distancing and self-isolation in a university setting during the COVID-19 pandemic: A qualitative study. *Int J Enviro Res Public Health* 2021;**18**. https://doi.org/10.3390/ijerph18084182.

24 de Camargo C. 'It's tough shit, basically, that you're all gonna get it': UK virus testing and police officer anxieties of contracting COVID-19.' *Policing Soc* 2021;**0**:1–17. https://doi.org/10.1080/10439463.2021.1883609.

25 Ljubić T, Banovac A, Buljan I, *et al.* Effect of SARS-CoV-2 antibody screening on participants' attitudes and behaviour: a study of industry workers in Split, Croatia. *Public Health* 2021;**191**:11–6. https://doi.org/10.1016/j.puhe.2020.12.001.

26 Missel M, Bernild C, Dagyaran I, *et al.* A stoic and altruistic orientation towards their work: a qualitative study of healthcare professionals' experiences of awaiting a COVID-19 test result. *BMC Health Serv Res* 2020;**20**:1–9. https://doi.org/10.1186/s12913-020-05904-0.

27 Allen WE, Altae-Tran H, Briggs J, *et al.* Population-scale longitudinal mapping of COVID-19 symptoms, behaviour and testing. *Nat Hum Behav* 2020;**4**:972–82. https://doi.org/10.1038/s41562-020-00944-2.

28 Wallis G, Siracusa F, Blank M, *et al.* Experience of a novel community testing programme for COVID-19 in London: Lessons learnt. *Clin Med (Lond)* 2020;**20**:E165–9. https://doi.org/10.7861/CLINMED.2020-0436.

[dataset] [29] Bauld, L, Aitman, T, Stagg, H, Connelly, R, Stage Baxter, M, Mortlet Corti, Y, Christianson, S, Street, A. Data from: TestEd Survey of Staff and Student Experiences and Perceptions of Novel Covid-19 Testing Platform, [dataset]. University of Edinburgh DataShare. February 1st 2023. https://doi.org/10.7488/ds/3802.

For peer review only - http://bmjopen.bmj.com/site/about/guidelines.xhtml

Supplementary File 1: TestEd Participant Pilot Survey

Notes for entry online:

BLUE = The question/variable name.

RED = Skip, display, or loop logic.

GREEN = New Block

PURPLE = Forced response

INTRO BLOCK

INTRODUCTION PAGE

I This survey is about student and staff experiences of asymptomatic COVID-19 testing at the University, delivered via the TestEd programme. Before agreeing to take part and proceeding to answer the survey questions, we'd like to remind you of what the survey involves and how the responses you provide will be used.

This information can also be found in the Participant Information Sheet for TestEd, which is available at:

https://www.ed.ac.uk/files/atoms/files/participant_information_sheet_v3.0_28_january_2021_clean.pdf

The survey will ask about your experiences of participating in TestEd. Your responses will help improve the programme as it is rolled out. Participation is voluntary and the survey should take about 10-15 minutes to complete.

Your anonymous survey data will be imported into quantitative data analysis software for analysis by the research team. The survey data will be retained on our server for a minimum of 5 years after the end of the study.

The anonymised results of this survey may be quoted in reports and academic publications produced by the study team. Your name will never be used in any of these reports or publications and they will not include any personal identifiable information about you.

At the end of this survey we ask if you would be willing to be re-contacted to participate in a follow-up interview with a researcher if you receive a positive result for Covid-19 from the TestEd programme. This interview is voluntary.

TestEd Participant Pilot Survey. 15th of April 2021

For peer review only

CONSENT

In agreeing to participate in this survey, you confirm the following:

1. I confirm that I have read and understood the Participant Information Sheet for TestEd.

2. I understand that my participation is voluntary and that I can ask to withdraw at any time without giving a reason and without my legal rights being affected.

3. I understand that once the survey form is submitted it will not be possible to withdraw from the survey. This is because no identifiable information will be stored with the survey data.

4. I confirm that I am happy for my survey responses to be linked to anonymised demographic data (age, gender, ethnicity, staff or student, whether living in University owned accommodation or elsewhere) provided by me when I registered to participate in the TestEd programme

5. I confirm that I am happy for anonymised data from this survey to be published for research purposes.

6. I understand that my anonymised data will be stored for a minimum of 5 years and may be used in future ethically approved research.

Should you have any further questions about this survey or any element of TestEd please contact us via TestEd@ed.ac.uk

By ticking this box, I agree to the above consent points and to take part in the above study

For peer review only

For peer review only - http://bmjopen.bmj.com/site/about/guidelines.xhtml

Q8

How much time did you take out of your day to provide a TestEd sample (not including travel time, i.e. collecting a sample pack, providing the sample and dropping off your sample)?

a. 1-2 minutes
b. 2-5 minutes
c. 5-10 minutes
d. More than 10 minutes

- -

TEST TRUST BLOCK

Q15

Do you believe that the result you received from Test Ed was accurate?

a. Yes
b. No
c. Unsure

Q16

Why did you believe the result was accurate/inaccurate?

[free text box]

Q17

Does the availability of the TestEd programme make you feel reassured about working/studying on campus?

a. Yes
b. No
c. Unsure [skip to Q19]

Q18

Could you explain a bit more about why you felt reassured or not?

[free text box]

- -

POST-TEST ATTITUDES AND BEHAVIOUR BLOCK

For peer review only

Q20

Have you changed your approach to public health guidelines (i.e. social distancing, face coverings, hygiene)

since you joined TestEd?

a. Yes
b. No [skip to Q24]
c. I don't know [skip to Q24]

Q21

Can you tell us about how your approach to public health guidelines has changed since your joined TestEd?

[free text box]

Q27

Overall, how would you rate your experience of the TestEd programme?

a. Excellent
b. Good
c. Fair
d. Poor
e. Very poor

Q28

Is there anything else you would like to tell us about your testing experience?
[free text box]

PAGE BREAK

For peer review only

For peer review only - http://bmjopen.bmj.com/site/about/guidelines.xhtml

Supplementary File 2: TestEd Participant Main Survey

Notes for entry online:

BLUE = The question/variable name.
RED = Skip, display, or loop logic.
GREEN = New Block
PURPLE = Forced response

- -

INTRO BLOCK

INTRODUCTION PAGE

Thank you very much for participating in TestEd!

Any feedback you are able to provide will help us to improve the system for you and for other users.

This survey is about student and staff experiences of the University of Edinburgh's COVID-19 testing project for people that are not showing any symptoms, TestEd. Before asking you to agree to take part, we'd like to remind you of what the survey involves and how the responses you provide will be used. Participation in this survey is entirely voluntary and the survey will take you about 10-15 minutes to complete.

This information can also be found in the Participant Information Sheet for TestEd, which is available at:

https://www.ed.ac.uk/files/atoms/files/participant_information_sheet_v5.0_01_september_2021.pdf

What will happen to my data?

The demographic data (age, gender, disability status, ethnicity, whether you are a student / staff, whether you live in university owned accommodation, and your department), that you provided when you joined TestEd and the dates when you provided a TestEd saliva sample will be linked to your survey responses using your TestEd barcode. Your anonymous survey data will then be imported into quantitative data analysis software for analysis by the research team. The survey data will be retained on our Sharepoint server for a minimum of 5 years after the end of the study.

The anonymised results of this survey may be quoted in reports and academic publications produced by the study team, which will help others to learn from TestEd's experience.Your name will never be used in any of these reports or publications and they will not include any personal identifiable information about you.

At the end of this survey we ask if you would be willing to be re-contacted to participate in a follow-up interview with a researcher from the TestEd programme. This interview is also entirely voluntary. If you tell us that you are interested in taking part in an interview, your demographic data will be shared with the TestEd interviewer.

PAGE BREAK

CONSENT

Thank you to those who completed a TestEd survey in Apr-21. We really want to hear from you again. This new survey contains some of the same questions that we asked you the last time. We really appreciate you taking the time to answer these again.

In agreeing to participate in this survey, you confirm the following:

1. I confirm that I have read and understood the Participant Information Sheet for TestEd V5.0 01 September 2021.

2. I understand that my participation is voluntary and that I can ask to withdraw at any time without giving a reason and without my legal rights being affected.

3. I understand that my rights to access, change or move my information are limited once the survey form is submitted and that you will keep the information provided even if I decide to withdraw from the survey or the TestEd study at a later date.

4. I confirm that I am happy for my survey responses to be linked to my anonymised demographic data (age, gender, disability status, ethnicity, whether you are a student / staff, whether you live in university owned accommodation, and your department) and dates when I provided TestEd saliva samples collected as part of the TestEd programme.

5. I confirm that I am happy for anonymised data from this survey to be published for research purposes.

6. I understand that my anonymised data will be stored for a minimum of 5 years and may be used in future ethically approved research.

7. I agree to take part in this TestEd survey.

Should you have any further questions about this survey or any element of TestEd please

For peer review only - http://bmjopen.bmj.com/site/about/guidelines.xhtml

contact us via TestEd@ed.ac.uk .

By ticking this box, I agree to the above consent points.

Q12

Please rank from most to least important what you believe are the benefits of taking part in TestEd (you may drag and drop from most to least important)

_______ To know own Covid-19 status in the absence of symptoms;

_______ To prevent from passing on infection to other colleagues/students on campus if I am positive;

_______ To prevent from passing on infection to family and friends outside the University if I am positive;

_______ To contribute to scientific research on Covid-19;

_______ Because other people are using TestEd, and I feel I should too.

PAGE BREAK

Q13

Are there any other benefit(s) to taking part in TestEd (optional)

[free text box]

PAGE BREAK

Q19

How much time did you take out of your day to provide a TestEd sample (i.e providing the sample and registering it on the system)?

a. 1-2 minutes
b. 2-5 minutes
c. 5-10 minutes
d. More than 10 minutes

PAGE BREAK

Q20

How convenient do you find it to provide a TestEd sample as part of your work/study schedule?

a. Very convenient [skip to Q22]
b. Convenient [skip to Q22]
c. Neutral [skip to Q23]
d. Inconvenient
e. Very inconvenient

PAGE BREAK

For peer review only

For peer review only - http://bmjopen.bmj.com/site/about/guidelines.xhtml

Q21

You have said that you find it inconvenient to provide a TestEd sample as part of your work/study schedule. Why is this? (optional)

[free text box]

PAGE BREAK

Q22

You have said that you find it convenient to provide a TestEd sample as part of your work/study schedule. Why is this? (optional)

[free text box]

PAGE BREAK

Q25

Prior to joining TestEd, how concerned were you about catching Covid-19 on campus?

a. Very concerned
b. Moderately concerned
c. Somewhat concerned
d. Slightly concerned
e. Not at all concerned [skip to Q27]

Q26

You have said that you had concerns about catching Covid-19 on campus prior to joining TestEd. Please briefly describe what were your main concerns (optional)

[free text box]

PAGE BREAK

Q27

You have said that you did not have concerns about catching Covid-19 on campus prior to joining TestEd why is this? (optional)

[free text box]

PAGE BREAK

Q28

Do you believe that the result(s) you received from Test Ed so far were accurate?

a. Yes
b. No
c. Unsure

For peer review only

PAGE BREAK

Q29

Why did you believe the result(s) were accurate/inaccurate?

[free text box]

PAGE BREAK

Q31

Does the availability of the TestEd programme make you feel reassured about working/studying on campus?

a. Yes
b. No
c. Unsure

- -

TEST TRUST BLOCK

Q32

Could you explain a bit more about why you felt reassured or not?

[free text box]

- -

POST-TEST ATTITUDES AND BEHAVIOUR BLOCK

Q33

Have you changed your approach to public health guidelines (i.e. social distancing, face coverings, hygiene) since you joined TestEd?

a. Yes [display 34 to Q35]
b. No [skip to Q35]
c. I don’t know [skip to Q35]

Q34

Can you tell us about how your approach to public health guidelines (i.e face coverings, hygiene) has changed since your joined TestEd?

For peer review only - http://bmjopen.bmj.com/site/about/guidelines.xhtml

[free text box]

PAGE BREAK

Q35

Overall, how would you rate your experience of the TestEd programme?

a. Excellent

b. Good

c. Fair

d. Poor

e. Very poor

For peer review only

Supplementary File 3 - Test-Ed COVID-19 Testing Project Interview Topic Guide

Round 2 Version 1.0 01 September 2021

1. Context – experience of the Covid-19 pandemic

Could you tell me a bit about your current role/work/study at the University of Edinburgh just for context? How long have you worked/studied here?

How has the Covid-19 pandemic affected your work/study at the University of Edinburgh since the beginning of 2020?

Prompt: change in working/study location/ change in routine/ contact with peers/colleagues

Prior to joining TestEd did you have any concerns about Covid-19 on campus? Why/What were these?

What kind of impact has the Covid-19 pandemic had on you personally over the past 20 months? [Follow up on leads from the answer to this question e.g. around travel to see family/friends; concerns about personal health/health of family and friends; personal experience of Covid infection prior to joining TestEd etc.]

And how would you describe the impact the pandemic is having on your life now? Do you feel that your life is back to normal and if not, what is different? Do you worry about Covid-19 in your everyday life right now? If so, in what way?

Before starting the TestEd programme, had you had any reason to get tested for Covid-19? Can you tell us about that experience?

Prompts: Why sought testing, experience of accessing a test, physical experience of undergoing testing, response to results

Before starting the TestEd programme, had you had any reason to isolate (prompt contacted by Test and Protect/ pinged by app)? Can you tell us about that experience?

Prompts: What did they find most difficult about isolating? Where they on their own? How did they get food? Was there any reason they had to leave the house?

And in terms of your personal circumstances, do you live with others at the moment? Prompt: type of housing (i.e. for students if in halls or elsewhere, for all - living with children, older adults etc.); Do you have any caring responsibilities for others (either inside or outside your household)?

For peer review only

Interview Consent Script and Topic Guide Round 2 V1.0 01 September 2021

2. Experience of TestEd

I would now like to ask a few more general questions about your experience of the TestEd programme.

Could you tell me how you heard about the programme (Test-Ed)? When was this?

Could you tell me why you decided to join the testing programme? What did you see as the main benefits of the TestEd programme?

Did you have any hesitation in joining the programme - Why?

Prompt: Any concerns about privacy of data?

Did you know anyone else who was already a part of the TestEd programme when you joined? Did you discuss your decision to join the programme with them (what did you discuss)?

Can you tell us about the process you went through to be tested for the first time?

Prompt: Where did they get tested? Did they understand what was required of them? What was their physical experience of the sample collection process? Did they have concerns about privacy related to the sample booth? How did this experience compare to any other Covid-19 testing experiences you have had (lateral flow/NHS PCR?). How quickly got results? Method for receiving results straightforward?

How have you made use of the TestEd programme since that first test?

Prompt: How often do you provide a sample? How do you fit the testing into your work/study routine? Are there any reasons why you have missed a test?

Prompt: How has your use of TestEd changed over time?

Prompt: Have you ever given a TestEd sample when you had symptoms linked to Covid-19 e.g. a cough or fever?

One concern that is often expressed about asymptomatic testing is that people might not follow public health guidelines (i.e. wearing face coverings, hand and respiratory hygiene etc) if they are being regularly tested. What is your view on that, and did you feel like that at all while you've been taking part in the TestEd programme?

Prompt: If they didn't feel like that, why not. If they did feel like that, how did it affect their behaviour?

For peer review only

Have you been vaccinated?

Could you tell me a bit more about when you had your first and second dose and timing of those?

Has being vaccinated changed how you feel about working/studying on campus? In what ways?

Do you think you have changed your everyday behaviour or routines in any way since you were vaccinated?

Prompt: Do you feel more protected since you have been vaccinated?

Has participation in the TestEd programme made you feel safer on campus? (note for interviewer: for those who report currently working/studying on campus)

Prompt: if not, why? Did you have any specific concerns about safety on campus prior to joining TestEd?

3. Story of Positive Test Result [for participants who have tested positive]

Can you tell me about your experience of testing positive for Covid-19 with TestEd? It would be really helpful if you could take us through your experience chronologically, starting before you were tested.

Prompts: Try to find out a clear timeline of events

Did you notice any changes in how you were feeling before you got tested?

What day of the week did you get tested and what else were you doing that day? What did you do after you gave your sample?

Where were you when you received the test result? Were you with anyone else? How did you feel when you got the result?

Did you have any worries or concerns **for yourself** following your positive test result? If so, could you tell us about them?

Did you have any worries or concerns **for others** following your positive test result? If so, could you tell us about them?

Did you think the test result was accurate at this point? Why? Why not?

Do you think your vaccination status affected your response to the test result?

What was your first response to the message? What did you do next? Did you tell anyone else your result at this point?

What contact did you have with the TestEd team? Did they give you advice on what to do next? What was that advice? Did you have any trouble following it?

Did you have food in the house? Did you have any other reason you needed to leave the house that day?

Did you need to rearrange plans because of the positive test result?

When did you book your NHS test? What did you do while you were waiting for your test? Can you take us through your experience of the NHS test? e.g. where did you go to get tested? How did you get there? What was your physical experience of the test? How did you feel while you were getting the test done? What did you do while waiting for the result? When did you get the result? How did that make you feel?

Did you have lateral flow tests in the house at the point that you received the test result? When and why had you ordered these (if not already addressed in previous questions). Did you or anyone else in your household use a lateral flow test at any point after you received the TestEd result? Can you tell us about your experience of this? Did you think the result was accurate (why/why not)?

Can you tell us about your experience of self-isolation?

Prompt: Did you feel clear about the self-isolation guidelines at the point that you tested positive? Were there any guidelines you found difficult to follow? How did you organise food (can also follow up on whether they used the food voucher they would have been given)? What was your daily routine during self-isolation? Did you have any reason you needed to leave the house during that period? If in shared house how did you manage your contact with other household members? What did you find most challenging about this period? Is there any other support that might have helped you self-isolate? Who from?

Did you experience any symptoms after your positive test? Prompt: talk through any symptoms or not after the TestEd positive result and

while waiting for/after the confirmatory test

To what extent did you trust the test result from TestEd? Why? Why not?

4. Closing questions

For peer review only

Interview Consent Script and Topic Guide Round 2 V1.0 01 September 2021

How could the TestEd testing experience have been improved?

Do you intend to continue participating in the TestEd programme? How do you think you will use it in the future?

Has your vaccination status affected your interest in participating in the programme at all?

Would you encourage others to participate in the TestEd programme. Prompt: if yes, why, if no, why not

Is there anything else you'd like to tell us about your experience of participating in the programme?

Would you be willing to be contacted for a short follow-up interview in the future?

Thank you for taking time to take part in this interview. [ENDS]

For peer review only

Supplementary File 4 – Survey Results (for both Pilot and Main Surveys) Tables

Pilot survey	Q8 Time taken to take test?				Q15 Belief in test result?			Q17 Does TestEd make you feel reassured?			Q20 Change in approach to public health guidelines?		Q27 Experience of TestEd programme?			
Response options / Demographics	1-2 min.	2-5 min.	5-10 min	>10 min	Yes	No	Unsure	Yes	No	Unsure	Yes	No	Excellent	Good	Fair	Poor
**Total**	223(42.7)	235(45.0)	57(10.9)	7(1.3)	472(90.4)	2(0.4)	48(9.2)	470(90.2)	17(3.3)	34(6.5)	16(3.1)	497(96.9)	408(78.2)	108)20.7)	6(1.2)	0(0.0)
**Gender** *n*(%)																
Female	151(45.6)	140(42.3)	37(11.2)	3(0.9)	300(90.6)	1(0.3)	30(9.1)	300(90.9)	8(2.4)	22(6.7)	9(2.8)	317(97.2)	252(76.1)	75(22.7)	4(1.2)	0(0.0)
Male	71(37.4)	95(50.0)	20(10.5)	4(2.1)	171(90.0)	1(0.5)	18(9.5)	168(89.0)	9(4.7)	12(6.3)	7(3.8)	179(96.2)	155(81.6)	33(17.4)	2(1.1)	0(0.0)
Comparison (p-value, ϕ_c)	FET = 0.128, ϕ_c = 0.103				FET = 0.942, ϕ_c = 0.021			χ^2 = 0.337, ϕ_c = 0.065			χ^2 = 0.502, ϕ_c = 0.030		FET = 0.400, ϕ_c = 0.059			
**Age (years)** *n*(%)																
≤19	0(0.0)	0(0.0)	0(0.0)	0(0.0)	0(0.0)	0(0.0)	0(0.0)	0(0.0)	0(0.0)	0(0.0)	0(0.0)	0(0.0)	0(0.0)	0(0.0)	0(0.0)	0(0.0)
20-29	77(44.8)	74(43.0)	19(11.1)	2(1.2)	148(86.1)	0(0.0)	24(14.0)	160(93.0)	4(2.3)	8(4.7)	7(4.1)	161(95.8)	134(77.9)	37(21.5)	1(0.6)	0(0.0)
30-39	58(42.0)	66(47.8)	14(10.1)	0(0.0)	123(89.1)	1(0.7)	14(10.1)	124(89.9)	4(2.9)	10(7.3)	1(0.7)	134(99.3)	106(76.8)	29(21.0)	3(2.2)	0(0.0)
40-49	34(41.5)	37(45.1)	9(11.0)	2(2.4)	76(92.7)	1(1.2)	5(6.1)	73(89.0)	4(4.9)	5(6.1)	2(2.4)	80(97.6)	62(75.6)	18(22.0)	2(2.4)	0(0.0)
50-59	38(46.9)	35(43.2)	7(8.6)	1(1.2)	77(95.1)	0(0.0)	4(4.9)	70(86.4)	3(3.7)	8(9.9)	5(6.3)	74(93.7)	65(80.3)	16(19.8)	0(0.0)	0(0.0)
≥60	16(32.7)	23(47.0)	8(16.3)	2(4.1)	48(98.0)	0(0.0)	1(2.0)	43(89.6)	2(4.2)	3(6.3)	1(2.0)	48(98.0)	31(83.7	8(16.3)	0(0.0)	0(0.0)
Comparison (p-value, ϕ_c)	χ^2 = 0.672, ϕ_c = 0.077				FET = 0.044, ϕ_c = 0.114			FET = 0.789, ϕ_c = 0.063			FET = 0.159, ϕ_c = 0.110		FET = 0.825, ϕ_c = 0.072			
**Ethnicity** *n*(%)																
British/Irish/Other white	203(42.8)	213(44.9)	51(10.8)	7(1.5)	432(91.1)	2(0.4)	40(8.4)	424(89.6)	16(3.4)	33(7.0)	11(2.4)	456(97.6)	369(77.9)	100(21.1)	5(1.1)	0(0.0)
Asian/Indian/Pakistani/Bangladeshi/ Chinese/Other Asian	12(48.0)	10(40.0)	3(12.0)	0(0.0)	21(84.0)	0(0.0)	4(16.0)	23(92.0)	1(4.0)	1(4.0)	2(8.7)	21(91.3)	19(76.0)	5(20.0)	1(4.0)	0(0.0)
Mixed/Other ethnic/Other black/Caribbean African	8(36.36)	11(50.0)	3(13.6)	0(0.0)	18(82.8)	0(0.0)	4(18.2)	22(100.0)	0(0.0)	0(0.0)	3(13.6)	19(86.4)	19(86.4)	3(13.6)	0(0.0)	0(0.0)
Comparison (p-value, ϕ_c)	FET = 0.950, ϕ_c = 0.038				FET = 0.264, ϕ_c = 0.062			FET = 0.760, ϕ_c = 0.053			χ^2 = 0.010, ϕ_c = 0.149		FET = 0.499, ϕ_c = 0.052			
**Role in the university** *n*(%)																
Staff	171(44.1)	173(44.6)	38(9.8)	6(1.6)	361(93.0)	2(0.5)	25(6.4)	344(88.9)	13(3.4)	30(7.8)	11(2.9)	372(97.1)	310(79.9)	73(18.8)	5(1.3)	0(0.0)
Students	52(38.8)	62(46.3)	19(14.2)	1(0.8)	111(82.8)	0(0.0)	23(17.2)	126(94.0)	4(3.0)	4(3.0)	5(3.9)	125(96.2)	98(73.1)	35(26.1)	1(0.8)	0(0.0)
Comparison (p-value, ϕ_c)	FET = 0.424, ϕ_c = 0.075				FET = 0.001, ϕ_c = 0.165			FET = 0.160, ϕ_c = 0.085			FET = 0.566, ϕ_c = -0.024		FET = 0.160, ϕ_c = 0.081			
**Disability** *n*(%)																
Yes	3(37.5)	4(50.0)	1(12.5)	0(0.0)	8(100.0)	0(0.0)	0(0.0)	8(100.0)	0(0.0)	0(0.0)	1(12.5)	7(87.5)	7(87.5)	1(12.5)	0(0.0)	0(0.0)
No	215(42.7)	226(44.9)	56(11.1)	6(1.2)	453(90.3)	2(0.4)	47(9.3)	453(90.2)	17(3.4)	32(6.4)	14(2.8)	480(97.2)	393(78.1)	104(20.7)	6(1.2)	0(0.0)
Comparison (p-value, ϕ_c)	FET = 1.000, ϕ_c = 0.020				FET = 1.000, ϕ_c = 0.041			FET = 1.000, ϕ_c = 0.041			FET = 0.217, ϕ_c = 0.071		FET = 0.100, ϕ_c = 0.029			

FET = Fisher's exact test, χ^2 = Chi Square test, ϕ_c = Cramer's V

For peer review only - http://bmjopen.bmj.com/site/about/guidelines.xhtml

Main survey	Q12 Most important motivations for taking part in TestEd?				
Demographics / Response options	To know Covid-19 status	To prevent infecting other colleagues/students on campus	To prevent infecting friends/family outside campus	To contribute to Covid-19 research	Because other people are using TestEd, so feel I should too
**Total**	753(38.4)	222(11.3)	619(31.5)	358(18.2)	11(0.6)
**Gender *n*(%)**					
Female	442(39.0)	129(11.4)	360(31.8)	196(17.3)	6(0.5)
Male	298(37.5)	87(11.0)	247(31.1)	157(19.8)	5(0.6)
Comparison (p-value, ϕ_c)	FET = 0.719, ϕ_c = 0.033				
**Age (years) *n*(%)**					
≤19	16(39.0)	11(26.8)	9(22.0)	5(12.2)	0(0.0)
20-29	232(40.5)	59(10.3)	181(31.6)	95(16.6)	6(1.1)
30-39	184(40.2)	49(10.7)	157(34.3)	67(14.6)	1(0.2)
40-49	134(35.5)	47(12.5)	124(32.9)	71(18.8)	1(0.3)
50-59	132(35.6)	37(10.0)	117(31.5)	84(22.6)	1(0.3)
≥60	55(38.5)	19(13.3)	31(21.7)	36(25.2)	2(1.4)
Comparison (p-value, ϕ_c)	χ^2 =0.005, ϕ_c = 0.071				
**Ethnicity *n*(%)**					
British/Irish/Other white	678(38.4)	196(11.1)	563(31.8)	324(18.3)	7(0.4)
Asian/Indian/Pakistani/Bangladeshi/ Chinese/Other Asian	46(43.8)	11(10.5)	28(26.7)	17(16.2)	3(2.9)
Mixed/Other ethnic/Other black/Caribbean African	25(32.1)	12(15.4)	24(30.8)	16(20.5)	1(1.3)
Comparison (p-value, ϕ_c)	FET = 0.092, ϕ_c = 0.063				
**Role in the university *n*(%)**					
Staff	538(37.8)	153(10.8)	460(32.4)	264(18.6)	7(0.5)
Students	208(40.0)	67(12.9)	151(29.0)	90(17.3)	4(0.8)
Comparison (p-value, ϕ_c)	FET = 0.361, ϕ_c = 0.046				
**Disability *n*(%)**					
Yes	22(43.1)	5(9.8)	14(27.5)	10(19.6)	0(0.0)
No	710(38.3)	204(11.0)	593(32.0)	334(18.0)	11(0.6)
Comparison (p-value, ϕ_c)	FET = 0.905, ϕ_c = 0.024				
**Staff role *n*(%)**					
Academic	273(37.6)	67(9.2)	243(33.5)	141(19.4)	2(0.3)
Facilities and estates	40(34.2)	17(14.5)	37(31.6)	21(18.0)	2(1.7)
Administration	94(39.8)	26(11.0)	67(28.4)	49(20.8)	0(0.0)
IT services	32(39.5)	11(13.6)	27(33.3)	11(13.6)	0(0.0)
Comparison (p-value, ϕ_c)	χ^2 = 0.231, ϕ_c = 0.066				

FET = Fisher's exact test, χ^2 = Chi Square test, ϕ_c = Cramer's V

For peer review only

Main survey	Q19 Time taken to take test?				Q20 Convenience to provide test?					Q25 Concern about catching Covid prior to joining TestEd?				
Response options / Demographics	1-2 min.	2-5 min.	5-10 min	>10 min	Very convenient	Convenient	Neutral	Inconvenient	Very inconvenient	Not at all concerned	Slightly concerned	Somewhat concerned	Moderately concerned	Very concerned
**Total**	857(41.8)	948(46.2)	208(10.2)	37(1.8)	1389(67.8)	524(25.6)	105(5.1)	31(1.5)	1(0.1)	131(6.4)	345(16.8)	458(22.3)	680(33.2)	436(21.3)
**Gender** *n*(%)														
Female	489(41.1)	561(47.1)	119(10.0)	21(1.8)	818 (68.7)	291(24.5)	60(5.0)	20(1.7)	1(0.1)	57(6.9)	149(18.1)	191(23.2)	270(32.8)	157(19.1)
Male	356(43.2)	369(44.8)	85(10.3)	14(1.7)	558(67.7)	214 (26.0)	43(5.0)	9(1.1)	0(0.0)	71(6.0)	190(16.0)	257(21.6)	402(33.8)	270(22.7)
Comparison (p-value, ϕ_c)	χ^2 = 0.825, ϕ_c = 0.022				FET = 0.732, ϕ_c = 0.035					χ^2 = 0.470, ϕ_c = 0.043				
**Age (years)** *n*(%)														
≤19	23(56.1)	16(39.0)	2(4.9)	0(0.0)	19(46.3)	15(36.6)	7(17.1)	0(0.0)	0(0.0)	4(9.8)	9(22.0)	9(22.0)	14(34.2)	5(12.2)
20-29	269(45.7)	266(45.2)	49(8.3)	5(0.9)	360(61.1)	186(31.6)	31(5.3)	12(2.0)	0(0.0)	30(5.1)	94(16.0)	160(27.2)	202(34.3)	103(17.5)
30-39	191(40.1)	222(46.6)	56(11.8)	7(1.5)	305(64.1)	131(27.5)	30(6.3)	10(2.1)	0(0.0)	27(5.7)	72(15.1)	101(21.2)	165(34.7)	111(23.3)
40-49	152(39.0)	193(49.5)	38(9.7)	7(1.8)	282(72.3)	88(22.6)	16(4.1)	4(1.0)	0(0.0)	340(7.7)	74(19.0)	82(21.0)	110(28.2)	94(24.1)
50-59	166(42.0)	177(44.8)	42(10.6)	10(2.5)	298(75.4)	79(20.0)	14(3.5)	31(0.8)	1(0.3)	28(7.1)	65(16.5)	85(21.5)	132(33.4)	85(21.5)
≥60	56(35.2)	74(46.5)	21(13.2)	8(5.0)	125(78.6)	25(15.7)	7(4.4)	2(1.3)	0(0.0)	12(7.6)	31(19.5)	21(13.2)	57(35.9)	38(23.9)
Comparison (p-value, ϕ_c)	χ^2 = 0.032, ϕ_c = 0.068				χ^2 <0.001, ϕ_c = 0.088					χ^2 = 0.032, ϕ_c = 0.065				
**Ethnicity** *n*(%)														
British/Irish/Other white	774(42.0)	852(46.2)	186(10.1)	33(1.8)	1262(68.4)	462(25.0)	93(5.0)	28(1.5)	1(0.1)	124(6.7)	320(17.3)	407(22.1)	622(33.7)	372(20.2)
Asian/Indian/Pakistani/Bangladeshi/ Chinese/Other Asian	50(45.1)	48(43.2)	12(10.8)	1(0.9)	61(55.0)	39(35.1)	9(8.1)	2(1.8)	0(0.0)	2(1.8)	12(10.8)	31(27.9)	36(32.4)	30(27.0)
Mixed/Other ethnic/Other black/Caribbean African	27(32.9)	44(53.7)	9(11.0)	2(2.4)	62(75.6)	17(20.7)	2(2.4)	1(1.2)	0(0.0)	5(6.1)	13(15.9)	15(18.3)	21(25.6)	28(34.2)
Comparison (p-value, ϕ_c)	FET =0.692, ϕ_c = 0.031				FET =0.093, ϕ_c = 0.055					χ^2 = 0.001, ϕ_c = 0.081				
**Role in the university** *n*(%)														
Staff	599(40.0)	695(46.5)	170(11.4)	31(2.1)	1055(70.6)	348(23.3)	68(4.6)	23(1.5)	1(0.1)	97(6.5)	259(17.3)	310(20.7)	498(33.3)	331(22.1)
Students	242(45.3)	248(46.4)	38(7.1)	6(1.1)	316(59.2)	173(32.4)	37(7.0)	8(1.5)	0(0.0)	31(5.8)	79(14.8)	143(26.8)	179(33.5)	102(19.1)
Comparison (p-value, ϕ_c)	χ^2 = 0.019, ϕ_c = 0.072				FET<0.001, ϕ_c = 0.112					χ^2 = 0.033, ϕ_c = 0.074				
**Disability** *n*(%)														
Yes	19(36.5)	26(50.0)	5(9.6)	2(3.9)	35(67.3)	11(21.2)	5(9.6)	1(2.0)	0(0.0)	0(0.0)	11(21.2)	13(25.0)	14(26.9)	14(26.9)
No	817(42.2)	889(45.9)	196(10.1)	33(1.7)	1318(68.1)	492(25.4)	96(5.0)	28(1.5)	1(0.1)	129(6.7)	327(16.9)	434(22.4)	646(33.4)	399(20.6)
Comparison (p-value, ϕ_c)	FET = 0.409, ϕ_c = 0.037				FET = 0.561, ϕ_c = 0.024					FET = 0.180, ϕ_c = 0.058				
**Staff role** *n*(%)														
Academic	308(40.8)	355(47.0)	77(10.2)	15(2.0)	512(67.8)	194(25.7)	37(4.9)	12(1.6)	0(0.0)	54(7.2)	130(17.2)	171(22.7)	260(34.4)	140(18.5)
Facilities and estates	57(41.1)	65(47.1)	14(10.1)	2(1.5)	112(81.2)	21(15.2)	4(2.9)	0(0.0)	1(0.7)	3(2.2)	21(15.2)	21(15.2)	43(31.2)	50(36.2)
Administration	85(34.3)	119(48.0)	37(14.9)	7(2.8)	184(74.2)	52(21.0)	7(2.8)	5(2.0)	0(0.0)	17(6.9)	51(20.6)	44(17.7)	73(29.4)	63(25.4)
IT services	36(43.9)	35(42.7)	10(12.2)	1(1.2)	56(68.3)	19(23.2)	6(7.3)	1(1.2)	0(0.0)	7(8.5)	13(15.9)	22(26.8)	26(31.7)	14(17.1)
Comparison (p-value, ϕ_c)	χ^2 = 0.586, ϕ_c = 0.046				χ^2 = 0.023, ϕ_c = 0.082					χ^2 = 0.004, ϕ_c = 0.913				

FET = Fisher's exact test, χ^2 = Chi Square test, ϕ_c = Cramer's V

For peer review only

Main survey	Q28 Belief in test result?			Q31 Does TestEd make you feel reassured?			Q33 Change in approach to public health guidelines?			Q35 Experience of TestEd programme?			
Response options / Demographics	Yes	No	Unsure	Yes	No	Unsure	Yes	No	I don't know	Excellent	Good	Fair	Poor
**Total**	1892(92.3)	4(0.2)	154(7.5)	1787(87.2)	99(4.8)	164(8.0)	94(4.6)	1922(93.3)	44(2.2)	1521(74.2)	500(24.4)	28(1.4)	1(0.1)
**Gender** ***n*****(%)**													
Female	1118(92.3)	1(0.1)	71(6.0)	1048(88.1)	46(3.9)	96(3.9)	49(4.1)	1117(93.9)	24(2.0)	889(74.7)	288(24.2)	12(1.0)	1(0.1)
Male	741(89.9)	2(0.2)	81(9.8)	708(85.9)	53(6.4)	63(7.7)	44(5.4)	761(92.5)	18(2.2)	611(74.2)	199(24.2)	14(1.7)	0(0.0)
Comparison (p-value, ϕ_c)	FET = 0.003, ϕ_c = 0.0735			χ^2 = 0.029, ϕ_c = 0.061			χ^2 = 0.244, ϕ_c = 0.038			χ^2 = 0.470, ϕ_c = 0.043			
**Age (years)** ***n*****(%)**													
≤19	39(95.1)	0(0.0)	2(4.9)	36(87.8)	0(0.0)	5(12.2)	2(5.0)	36(90.0)	2(5.0)	22(52.7)	17(41.5)	2(4.9)	0(0.0)
20-29	536(91.0)	2(0.3)	51(8.7)	528(89.6)	22(3.7)	39(6.6)	29(4.9)	539(91.5)	21(3.6)	398(67.6)	179(30.4)	11(1.9)	1(0.2)
30-39	437(91.8)	0(0.0)	39(8.2)	406(85.3)	28(5.9)	42(8.8)	25(5.2)	441(92.7)	10(2.1)	328(68.9)	140(29.4)	8(1.7)	0(0.0)
40-49	362(92.8)	1(0.3)	27(7.0)	340(87.2)	22(5.6)	28(7.2)	15(3.9)	372(95.4)	3(0.8)	308(79.0)	80(20.5)	2(0.5)	0(0.0)
50-59	366(92.7)	1(0.3)	28(7.1)	338(85.6)	19(4.8)	38(9.6)	7(1.8)	383(97.0)	5(1.3)	333(84.3)	61(15.4)	1(0.3)	0(0.0)
≥60	152(95.6)	0(0.0)	7(4.4)	139(87.4)	8(5.0)	12(7.6)	16(10.1)	140(88.1)	3(1.9)	132(83.0)	23(14.5)	4(2.5)	0(0.0)
Comparison (p-value, ϕ_c)	FET = 0.770, ϕ_c = 0.038			χ^2 = 0.326, ϕ_c = 0.054			χ^2 <0.001, ϕ_c = 0.092			χ^2 <0.001, ϕ_c = 0.126			
**Ethnicity** ***n*****(%)**													
British/Irish/Other white	1711(92.7)	3(0.2)	131(7.1)	1604(86.9)	89(4.8)	152(8.2)	80(4.3)	1729(93.8)	35(1.9)	1390(75.3)	429(23.3)	25(1.4)	1(0.1)
Asian/Indian/Pakistani/Bangladeshi/ Chinese/Other Asian	94(84.7)	0(0.0)	17(15.3)	102(92.0)	3(2.7)	6(5.4)	8(7.2)	96(86.5)	7(6.3)	70(63.1)	41(37.0)	0(0.0)	0(0.0)
Mixed/Other ethnic/Other black/Caribbean African	75(91.5)	1(1.2)	6(7.3)	71(86.6)	6(7.3)	5(6.1)	5(6.1)	75(91.5)	2(2.4)	51(62.2)	28(34.2)	3(3.7)	0(0.0)
Comparison (p-value, ϕ_c)	FET = 0.005, ϕ_c = 0.069			FET = 0.296, ϕ_c = 0.037			FET = 0.058, ϕ_c = 0.048			FET =0.001, ϕ_c = 0.070			
**Role in the university** ***n*****(%)**													
Staff	1382(92.4)	2(0.1)	111(7.4)	1293(86.5)	78(5.2)	124(8.3)	67(4.5)	1398(93.5)	30(2.0)	1162(77.7)	320(21.4)	13(0.9)	0(0.0)
Students	491(92.0)	2(0.4)	41(7.7)	474(88.8)	21(3.9)	39(7.3)	27(5.1)	492(92.3)	14(2.6)	341(63.9)	177(33.2)	15(2.8)	1(0.2)
Comparison (p-value, ϕ_c)	FET = 1.000, ϕ_c = 0.006			χ^2 = 0.188, ϕ_c = 0.042			χ^2 = 0.627, ϕ_c = 0.022			χ^2 <0.001, ϕ_c = 0.150			
**Disability** ***n*****(%)**													
Yes	51(98.1)	0(0.0)	1(1.9)	47(90.4)	1(1.9)	4(7.7)	4(7.7)	46(88.5)	2(3.9)	34(65.4)	18(34.6)	0(0.0)	0(0.0)
No	1782(92.1)	4(0.2)	149(7.7)	1690(87.3)	94(4.9)	151(7.8)	86(4.5)	1810(93.6)	38(2.0)	1449(74.9)	460(23.8)	25(1.3)	1(0.1)
Comparison (p-value, ϕ_c)	FET = 0.241, ϕ_c = 0.036			FET = 0.757, ϕ_c = 0.022			FET = 0.175, ϕ_c = 0.038			FET = 0.130, ϕ_c = 0.049			
**Staff role** ***n*****(%)**													
Academic	690(91.4)	1(0.1)	64(8.5)	657(87.0)	41(5.4)	57(7.6)	22(2.9)	721(95.5)	12(1.6)	593(78.5)	157(20.8)	5(0.7)	0.(0.0)
Facilities and estates	132(95.7)	1(0.7)	5(3.6)	121(87.7)	5(3.6)	12(8.7)	19(13.8)	113(81.9)	6(4.4)	111(80.4)	26(18.8)	1(0.7)	0.(0.0)
Administration	230(92.7)	0(0.0)	18(7.3)	215(86.7)	9(3.6)	24(9.7)	11(4.4)	235(94.8)	2(0.8)	193(77.8)	54(21.8)	1(0.4)	0.(0.0)
IT services	76(92.7)	0(0.0)	6(7.3)	63(76.8)	7(8.5)	12(14.6)	1(1.2)	78(95.1)	3(3.7)	61(74.4)	20(24.4)	1(1.2)	0.(0.0)
Comparison (p-value, ϕ_c)	FET = 0.308, ϕ_c = 0.054			FET = 0.105, ϕ_c = 0.067			FET <0.001, ϕ_c = 0.137			FET = 0.712, ϕ_c = 0.034			

FET = Fisher's exact test, χ^2 = Chi Square test, ϕ_c = Cramer's V

For peer review only

For peer review only

STROBE Statement—Checklist of items that should be included in reports of ***cross-sectional studies***

	Item No	Recommendation	Page No
**Title and abstract**	1	(*a*) Indicate the study's design with a commonly used term in the title or the abstract	2
		(*b*) Provide in the abstract an informative and balanced summary of what was done and what was found	2
**Introduction**			
Background/rationale	2	Explain the scientific background and rationale for the investigation being reported	3
Objectives	3	State specific objectives, including any prespecified hypotheses	3
**Methods**			
Study design	4	Present key elements of study design early in the paper	4
Setting	5	Describe the setting, locations, and relevant dates, including periods of recruitment, exposure, follow-up, and data collection	4
Participants	6	(*a*) Give the eligibility criteria, and the sources and methods of selection of participants	4
Variables	7	Clearly define all outcomes, exposures, predictors, potential confounders, and effect modifiers. Give diagnostic criteria, if applicable	4
Data sources/ measurement	8*	For each variable of interest, give sources of data and details of methods of assessment (measurement). Describe comparability of assessment methods if there is more than one group	4
Bias	9	Describe any efforts to address potential sources of bias	4
Study size	10	Explain how the study size was arrived at	4
Quantitative variables	11	Explain how quantitative variables were handled in the analyses. If applicable, describe which groupings were chosen and why	4
Statistical methods	12	(*a*) Describe all statistical methods, including those used to control for confounding	4
		(*b*) Describe any methods used to examine subgroups and interactions	4
		(*c*) Explain how missing data were addressed	4
		(*d*) If applicable, describe analytical methods taking account of sampling strategy	n/a
		(*e*) Describe any sensitivity analyses	n/a
**Results**			
Participants	13*	(a) Report numbers of individuals at each stage of study—eg numbers potentially eligible, examined for eligibility, confirmed eligible, included in the study, completing follow-up, and analysed	5
		(b) Give reasons for non-participation at each stage	5
		(c) Consider use of a flow diagram	n/a
Descriptive data	14*	(a) Give characteristics of study participants (eg demographic, clinical, social) and information on exposures and potential confounders	5-6
		(b) Indicate number of participants with missing data for each variable of interest	5
Outcome data	15*	Report numbers of outcome events or summary measures	n/a
Main results	16	(*a*) Give unadjusted estimates and, if applicable, confounder-adjusted estimates and their precision (eg, 95% confidence interval). Make clear which confounders were adjusted for and why they were included	SF4

For peer review only

		(*b*) Report category boundaries when continuous variables were categorized	n/a
		(*c*) If relevant, consider translating estimates of relative risk into absolute risk for a meaningful time period	n/a
Other analyses	17	Report other analyses done—eg analyses of subgroups and interactions, and sensitivity analyses	n/a
**Discussion**			
Key results	18	Summarise key results with reference to study objectives	5-12
Limitations	19	Discuss limitations of the study, taking into account sources of potential bias or imprecision. Discuss both direction and magnitude of any potential bias	13
Interpretation	20	Give a cautious overall interpretation of results considering objectives, limitations, multiplicity of analyses, results from similar studies, and other relevant evidence	12-14
Generalisability	21	Discuss the generalisability (external validity) of the study results	12-14
**Other information**			
Funding	22	Give the source of funding and the role of the funders for the present study and, if applicable, for the original study on which the present article is based	18

*Give information separately for exposed and unexposed groups.

Note: An Explanation and Elaboration article discusses each checklist item and gives methodological background and published examples of transparent reporting. The STROBE checklist is best used in conjunction with this article (freely available on the Web sites of PLoS Medicine at http://www.plosmedicine.org/, Annals of Internal Medicine at http://www.annals.org/, and Epidemiology at http://www.epidem.com/). Information on the STROBE Initiative is available at www.strobe-statement.org.

For peer review only
